# SUPER: Upcycling Genetic Parts for Precise Gene Expression Control, Leakage Minimization, and Genetic Circuit Stability

**DOI:** 10.1002/advs.202514653

**Published:** 2025-12-15

**Authors:** Taeyang Heo, Dongwon Park, Woosub Shin, Jongmin Kim

**Affiliations:** ^1^ Department of Life Sciences Pohang University of Science and Technology Pohang 37673 South Korea

**Keywords:** biosensor, genetic circuit stability, genetic device, kill switch, leakage minimization, small RNA, synthetic biology

## Abstract

A long‐standing goal of synthetic biology is to reprogram cells by rewiring genetic parts. Despite the expanding library of genetic parts, construction of integrated synthetic circuits with desired specifications remains challenging in part due to intricate dependence on sequence contexts, where unexpected narrow dynamic ranges and leaky expression can plague system performance. To provide an alternative approach to the screening process of iterative design‐build‐test cycles, **SUPER** (**S** ynthetic **U** pcycling **P** latform for **E** ngineering **R** egulators), a modular platform for upcycling genetic devices is introduced. Inspired by antagonistic regulation mechanisms, SUPER employs small RNA as an add‐on controller to modulate gene expression patterns without genetic modification of target regulators. SUPER not only enhances the performance of RNA‐, chemical‐, temperature‐, and protein‐responsive regulators up to 1011%, but also allows to cover an expanded dynamic range up to 22 018.9‐fold. This enhanced control can provide genetic circuit stability, particularly under strong selective pressures, as demonstrated with a Holin‐expressing kill switch integrated with SUPER, maintaining stable functionality for over 30 days. Finally, SUPER combines with an environmental sensor, TlpA36, functioning as a chemical‐ and temperature‐responsive 2‐input kill switch. Featuring straightforward design, minimal cellular burden, and expanded tunability, SUPER provides a systematic upcycling framework for genetic circuit construction in biotechnology.

## Graphical abstract

To precisely modulate natural and synthetic regulatory parts, SUPER is introduced as a sophisticated and tunable controller of genetic devices without requiring sequence modification on the target. SUPER enhances dynamic range of RNA‐, chemical‐, temperature‐, and protein‐responsive biosensors up to 1011%, and converts biosensors to cover an expanded dynamic range up to 22 018.9‐fold. This stringent control with SUPER results in long‐term genetic stability of synthetic kill switches up to 30 days in a microbial co‐culture setting, suggesting a broad range of potential applications in bioengineering and biotherapeutics.

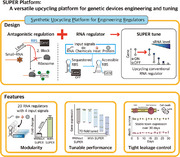

## 1 Introduction

The goal of synthetic biology is to program cellular behavior by constructing genetic circuits in much the same way as we program electronic circuits.^[^ ^1^ ^]^ By harnessing reprogrammed cells, synthetic biology offers sustainable solutions across health,^[^ ^2^, ^3^, ^4^ ^]^ industry,^[^ ^5^, ^6^ ^]^ agriculture,^[^ ^7^, ^8^ ^]^ and environment.^[^ ^9^, ^10^, ^11^ ^]^ Engineered cells equipped with synthetic circuits could enable environmental sensing and remediation through whole‐cell biosensors that detect and capture pollutants,^[^ ^9^, ^11^, ^12^, ^13^, ^14^ ^]^ degrade and recycle plastics,^[^ ^15^, ^16^, ^17^, ^18^ ^]^ as well as carbon capture and utilization.^[^ ^19^, ^20^, ^21^, ^22^ ^]^ Synthetic biology is also emerging as a powerful alternative for disease detection and treatment including biomarker diagnostics,^[^ ^23^, ^24^, ^25^ ^]^ cell‐based therapies,^[^ ^4^, ^26^, ^27^, ^28^ ^]^ and microbiome engineering.^[^ ^29^, ^30^, ^31^, ^32^, ^33^ ^]^

To implement sophisticated genetic circuits, a large library of well‐characterized and modular biological parts is one of the basic requirements.^[^ ^34^, ^35^ ^]^ Early efforts focused on assembling simple genetic constructs using standardized parts such as promoters, ribosome binding sites (RBS), and repressors,^[^ ^36^, ^37^, ^38^, ^39^, ^40^ ^]^ enabling the construction of toggle switches,^[^ ^41^, ^42^, ^43^ ^]^ oscillators,^[^ ^44^, ^45^, ^46^ ^]^ and logic gates.^[^ ^47^, ^48^, ^49^, ^50^ ^]^ To support the construction of increasingly complex genetic circuits, there is a pressing need for more diverse and tunable genetic elements.^[^ ^51^, ^52^, ^53^, ^54^, ^55^ ^]^ Toward this, RNA‐based regulatory elements, or riboregulators, provide promising building blocks due to their design flexibility,^[^ ^56^, ^57^, ^58^, ^59^ ^]^ modularity,^[^ ^60^ ^]^ rapid response,^[^ ^60^, ^61^, ^62^, ^63^ ^]^ and low cellular burden.^[^ ^64^, ^65^, ^66^ ^]^ Natural and synthetic riboregulators have been repurposed for synthetic biological circuitry including riboswitches,^[^ ^67^, ^68^, ^69^, ^70^, ^71^ ^]^ ribozymes,^[^ ^72^, ^73^, ^74^, ^75^ ^]^ microRNAs,^[^ ^76^, ^77^, ^78^ ^]^ small RNAs,^[^ ^66^, ^79^, ^80^, ^81^, ^82^, ^83^ ^]^ and RNA‐responsive CRISPR‐Cas systems,^[^ ^84^, ^85^, ^86^, ^87^, ^88^ ^]^ with applications in viral detection,^[^ ^89^, ^90^ ^]^ genotyping,^[^ ^91^, ^92^, ^93^ ^]^ metabolite monitoring,^[^ ^94^, ^95^, ^96^ ^]^ metabolic flux regulation,^[^ ^66^, ^79^, ^83^, ^97^ ^]^ and complex cellular logic computation.^[^ ^86^, ^98^, ^99^, ^100^, ^101^ ^]^

Despite efforts in developing context‐independent, modular, and orthogonal parts, ensuring compatibility and reliable performance when assembling heterogeneous parts remains a key challenge.^[^ ^99^, ^102^, ^103^, ^104^ ^]^ In particular, low dynamic ranges, defined as the ratio of the output in the ON state to the output in the OFF state, and leakage from regulatory parts often hamper the construction of genetic circuits by disrupting signal transmission, increasing noise, and limiting tunability.^[^ ^98^, ^105^, ^106^, ^107^ ^]^ To achieve desired operational outputs, a screening process on different subparts such as promoters, RBS, transcription factors, and coding sequences are often required.^[^ ^108^, ^109^, ^110^, ^111^ ^]^ These issues are exacerbated under strong selection pressure, as exemplified by synthetic kill switch circuits for biocontainment. Since these synthetic kill switch circuits harbor toxins as the outputs, any leaky expression greatly reduces the viability of the engineered host, leading to inactivation of the synthetic circuits by mutation.^[^ ^107^, ^112^, ^113^, ^114^ ^]^ While conventional iterative approaches, comprising repetitive cycles of design, build, and test, are effective for circuit optimization, there is a tradeoff that these processes are often labor‐intensive and time‐consuming.^[^ ^115^, ^116^, ^117^, ^118^, ^119^, ^120^ ^]^ To mitigate these issues and provide more reliable genetic components, large scale screening efforts together with parts mining from diverse hosts, high‐throughput characterization, and computer‐aided designs could be explored.^[^ ^98^, ^99^, ^121^, ^122^, ^123^ ^]^ Alternatively, precisely modulating existing genetic parts may offer an effective strategy to minimize the need for component redesign and streamline synthetic circuit development.

In a similar vein, diverse optimization strategies have been explored to achieve predictable and tunable performance for riboregulators. Rational design, aided by thermodynamic modeling or RNA structure predictions, provides a systematic framework but also requires detailed mechanistic understanding.^[^ ^124^, ^125^, ^126^ ^]^ Library‐based screening^[^ ^127^, ^128^, ^129^, ^130^ ^]^ and directed evolution^[^ ^131^, ^132^, ^133^, ^134^ ^]^ can help find functional parts and expand sequence space, with the challenge of limited scalability and heavy resource burden. Data‐driven approaches including machine learning‐guided optimization show promise, yet are still in the early stages.^[^ ^122^, ^123^, ^135^, ^136^ ^]^ These efforts illustrate the great potential for multiple optimization strategies, yet at the same time, underscore the limits in principled and easy‐to‐adopt design paradigm, highlighting the need for complementary approaches to modulate and engineer existing regulatory parts without iterative design cycles.

Recently, to achieve precise and tunable gene expression control, bioengineers have employed molecular antagonists such as anti‐CRISPR proteins,^[^ ^137^, ^138^ ^]^ transcription factor sponge DNAs,^[^ ^139^ ^]^ and miRNA sponge RNAs.^[^ ^140^ ^]^ Because these synthetic antagonistic strategies function as add‐on controllers, they can be implemented without directly modifying existing regulatory devices. Inspired by these efforts, we aimed to develop a genetic parts upcycling platform by employing an antagonistic mechanism against protein translation activation, using trans‐acting small RNAs (sRNAs). sRNAs are post‐transcriptional repressors that bind to complementary sequences near the RBS of their target mRNAs, thereby inhibiting translational initiation.^[^ ^141^, ^142^, ^143^, ^144^ ^]^ Due to their minimal sequence constraints, sRNAs can be easily designed to target diverse sequences,^[^ ^58^, ^66^, ^80^, ^81^, ^82^ ^]^ enabling our upcycling platform to function in a modular manner with a broad range of existing genetic devices.

Here, we introduce a novel strategy called Synthetic Upcycling Platform for Engineering Regulators (SUPER), where synthetic sRNAs are exploited as add‐on controllers at the post‐transcriptional layer to improve dynamic range, ensure genetic stability, and reduce leaky expression of genetic devices. As a starting point, we validated the performance, robustness, and specificity of sRNA‐mediated repression on synthetic genetic devices. Next, we enhanced the dynamic ranges of genetic devices by applying SUPER together with RNA switches,^[^ ^59^, ^100^, ^145^ ^]^ riboswitches,^[^ ^71^, ^146^ ^]^ and RNA‐based thermosensors.^[^ ^147^ ^]^ Furthermore, a Holin‐expressing kill switch for biocontainment was constructed and tested together with leakage control by SUPER, thereby ensuring long‐term genetic stability and improved performance in a co‐culture setting. This enhanced kill switch performance was maintained when combined with a temperature‐responsive transcription factor, TlpA36,^[^ ^148^ ^]^ for a chemical‐ and temperature‐responsive 2‐input kill switch. Together, SUPER, as a sophisticated and tunable controller of genetic devices, demonstrated robust enhancement of dynamic ranges across multiple devices, ensured long‐term genetic stability with environmental sensing capability, providing a novel genetic parts upcycling platform as versatile add‐on controllers for synthetic biology, bioengineering, and biomedical applications.

## 2 Results

### 2.1 Post‐Transcriptional Repression Using Synthetic sRNAs

**Figure 1:**
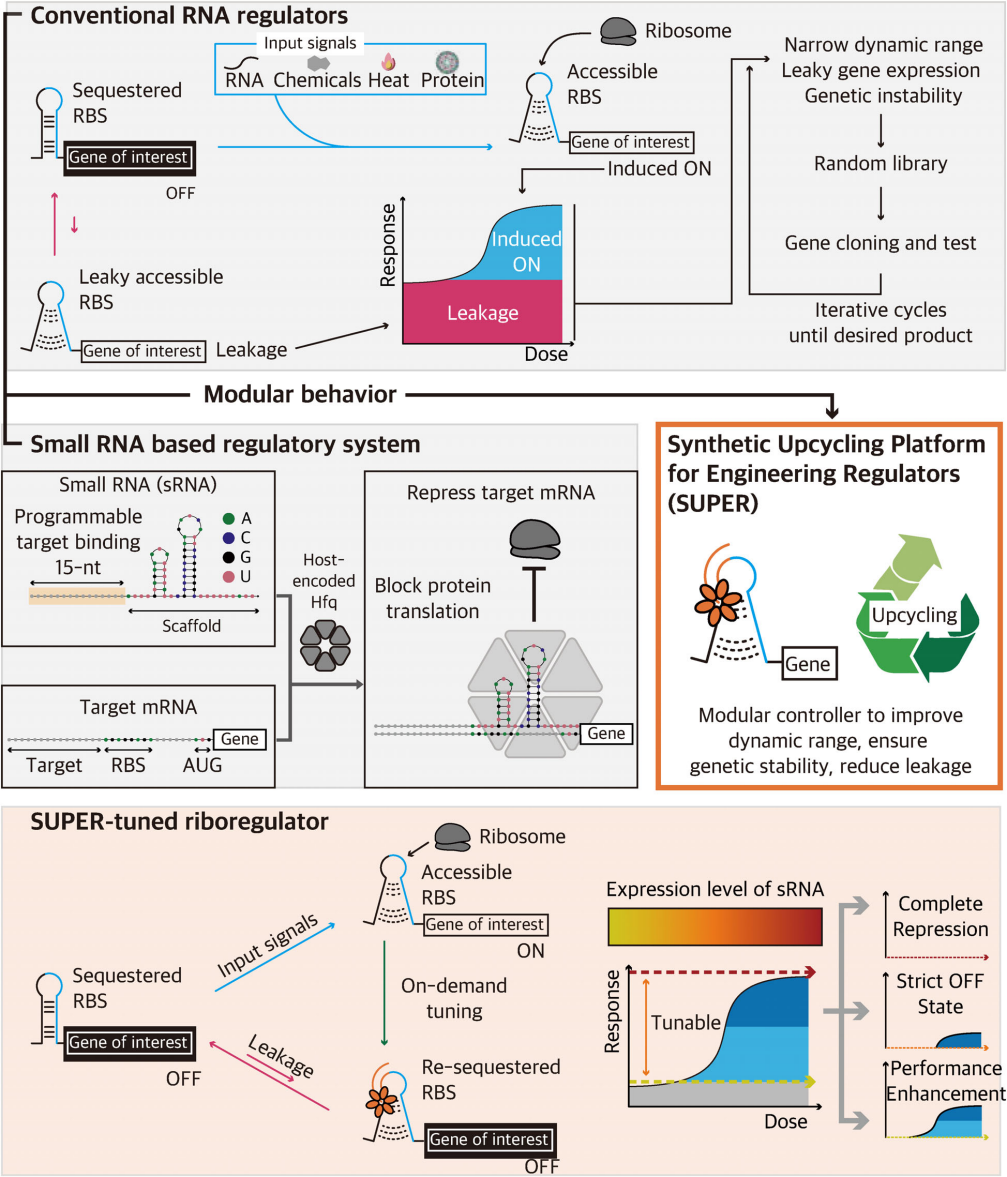
Schematic overview of SUPER for upcycling the existing suite of regulators. (Top panel) Translational RNA regulators control gene expression by modulating mRNA conformation in response to environmental inputs such as RNAs, chemicals, temperature, or proteins. Although the closed conformation is thermodynamically favored, RNA regulator molecules can undergo spontaneous and reversible unwinding, transiently exposing the RBS and leading to leaky gene expression. The leaky expression from regulators reduces the dynamic range and could also lead to genetic instability. To achieve desired circuit performance, iterative cycles of design, build, and test to achieve an optimized module with desired traits are often required. (Middle left panel) Schematics for the sequence, structure, and mechanism of synthetic sRNAs that repress the translation initiation of target mRNAs. (Middle right panel) Upcycling genetic parts via SUPER. By using synthetic sRNAs to control RNA regulators, SUPER acts as a modular platform to improve dynamic ranges, ensure genetic stability, and reduce system leakage. (Bottom panel) SUPER utilizes rational sRNA designs for controlling regulator performance. By adjusting the sRNA expression levels, SUPER‐tuned RNA regulators can exhibit multiple expression profiles, including complete repression, leakage minimization, and enhanced dynamic range.

To enable precise and tunable control of natural and synthetic RNA devices (**Figure** **1**, top panel), we aimed to utilize sRNA as the medium for synthetic antagonist for protein translation. sRNAs are non‐coding RNAs that regulate messenger RNAs (mRNAs) at the post‐transcriptional level (Figure 1, middle left panel). In nature, sRNAs rapidly repress cognate mRNAs, facilitating adaptation to sudden environmental stress.^[^ ^141^, ^149^, ^150^ ^]^ There are four key features that position sRNAs as a suitable candidate for sophisticated and tunable modular controller for riboregulators. First, sRNA has been widely used for both endogenous and synthetic systems, demonstrating functional robustness.^[^ ^66^, ^79^, ^141^, ^149^, ^150^ ^]^ Second, as a trans‐acting post‐transcriptional repressor, synthetic sRNA can be rationally designed to selectively bind to target mRNAs through sequence complementarity.^[^ ^66^ ^]^ Third, the efficacy of sRNA‐mediated repression is correlated with the expression level,^[^ ^81^, ^83^, ^151^ ^]^ thereby providing a straightforward way to continuously tune expression patterns. Lastly, sRNA imposes minimal cellular burden to help ensure the long‐term stability of engineered systems.^[^ ^64^, ^79^ ^]^

To leverage synthetic sRNA as a versatile post‐transcriptional antagonist to precisely tune the riboregulators of choice (Figure 1, middle right and bottom panel), we initially set out to evaluate the performance of synthetic sRNAs in *Escherichia coli* (*E. coli*), following standard design and experimental procedures. All synthetic sRNAs were designed to consist of a 15‐nucleotide (nt) target binding site and an Hfq binding scaffold, adapted from Spot42 (Figure 1, middle left panel; Figure S1a–c, Supporting Information), to enhance interaction stability and efficiency.^[^ ^83^, ^152^ ^]^ Following this design rule with NUPACK‐guided sequence selection, the synthetic sRNA repressed GFP expression by over 6000‐fold, and the strength of sRNA‐mediated repression was correlated with the sRNA abundance (Figure S1d, Supporting Information).^[^ ^153^, ^154^ ^]^

To test the applicability of sRNA control on riboregulators, the toehold switch (THS) was selected as a target synthetic RNA device due to the characteristic features of THS such as detailed mechanistic understanding, well‐established design rules, and a large library size with wide dynamic ranges.^[^ ^59^, ^89^, ^90^, ^122^, ^123^, ^155^ ^]^ A THS contains a stable hairpin structure that sequesters the RBS and start codon, preventing ribosome access and translation initiation. When a cognate trigger RNA is expressed, the cognate trigger RNA initially binds to the toehold domain of THS, and continues the branch migration process to unfold the hairpin structure, thereby exposing the RBS and start codon for translation initiation of the downstream gene.^[^ ^59^ ^]^ Notably, THS can accommodate loop modifications and extensions, thereby providing ample room for sRNA target domain engineering.^[^ ^156^, ^157^, ^158^ ^]^

**Figure 2:**
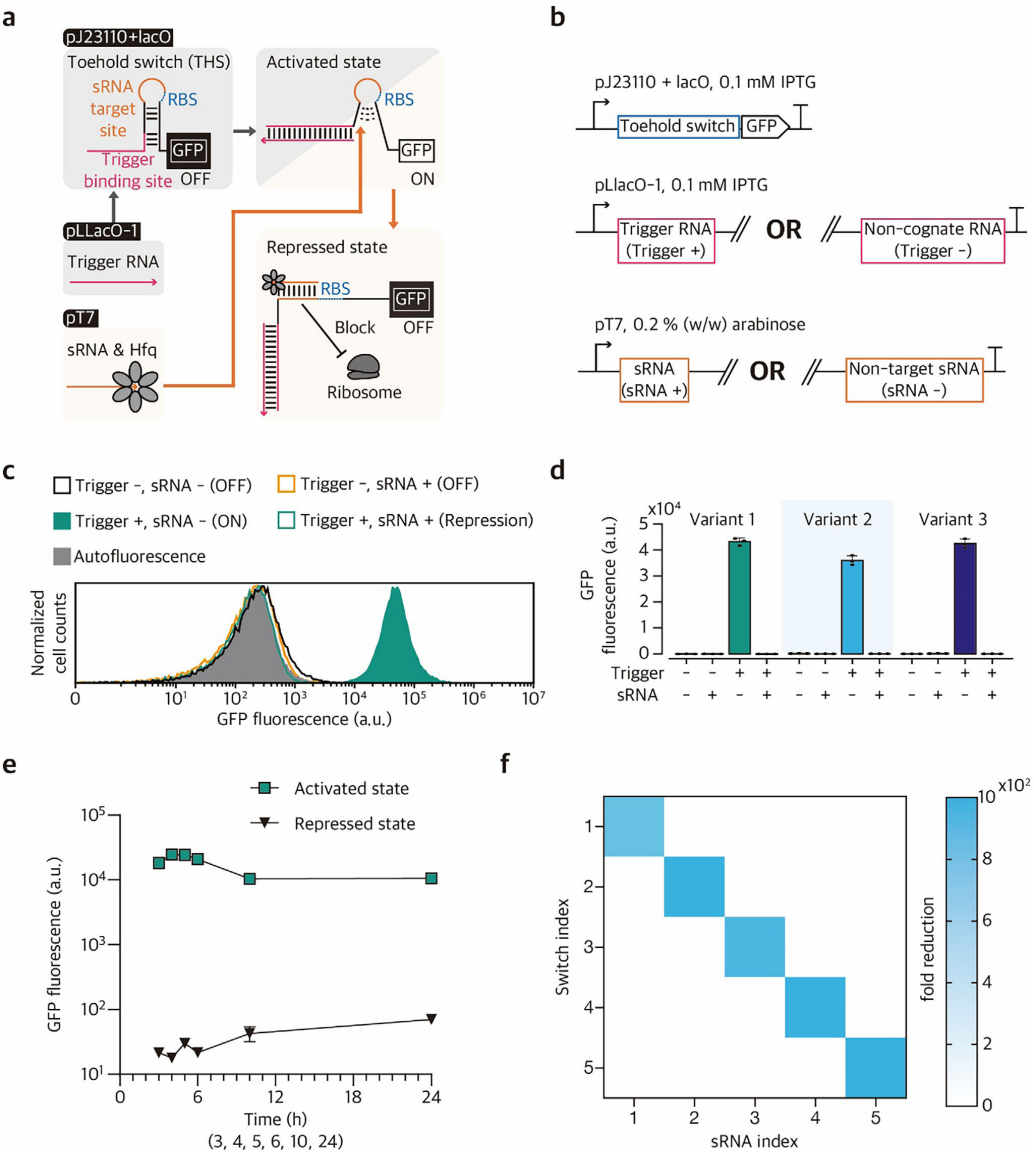
Schematic of sRNA‐mediated post‐transcriptional repression and characterization in vivo. a) Post‐transcriptional repression of an activated toehold switch (THS) using synthetic sRNA. THS restricts translation initiation by sequestering the ribosome binding site (RBS) and start codon within a hairpin. In the presence of a cognate trigger, the hairpin structure can be unwound through strand displacement, exposing the RBS and start codon, thereby allowing active translation. The binding of sRNA with host‐encoded Hfq to the RBS of activated THS can block the ribosome entry. b) Expression cassettes of switch RNA, trigger RNA, and sRNA. Switch RNA is expressed from a strong constitutive promoter pJ23110 with lacO for isopropyl β‐D‐1‐thiogalactopyranoside (IPTG)‐inducible expression, and cognate trigger is expressed from the IPTG‐inducible promoter pLlacO‐1. sRNA is under the control of T7 promoter, which in turn is induced by L‐arabinose (ara) in *E. coli* BL21‐AI that harbors a genomic copy of T7 RNA polymerase under the pBAD promoter. Trigger (+) indicates the presence of the cognate trigger, whereas trigger (–) indicates the presence of a non‐cognate trigger RNA. At the same time, sRNA (+) denotes the presence of cognate sRNA, while sRNA (–) refers to the presence of non‐targeting sRNA without the target‐binding sequence. c) Flow cytometry analysis of THS in the presence and absence of its cognate trigger and sRNA. d) THS repression with sRNA variants. The 15‐nt target binding domain in sRNA was modified to be complementary to the corresponding target sequence within the THS hairpin. e) Time‐course measurement of sRNA‐mediated repression. f) Orthogonality matrix for five sRNAs and their target switches. Except for the time‐course measurement (e), fluorescence of cells was measured via flow cytometry 4 h 30 min after induction. The number of biological replicates is three, and the fold reduction was calculated as the average across all 9 combinations of 3 activated samples (Trigger +, sRNA –) and 3 repressed samples (Trigger +, sRNA +). Error bars are the standard deviation of three biological replicate measurements.

To enable efficient sRNA‐mediated suppression of an activated THS, we placed the 15‐nt sRNA target site immediately upstream of the RBS (**Figure** **2**a; Figure S2, Supporting Information). During the sRNA design process, the sequence and structure of sRNA and its target domain were optimized while unintended sRNA target site interaction with other domains was minimized using in‐silico design tool NUPACK.^[^ ^153^, ^154^ ^]^ While sRNA can be designed to target a wide range of potential regions within mRNA, recent studies showed that targeting the immediate upstream region of the RBS with sRNA generally yields good performance, robustness, and sequence specificity.^[^ ^66^, ^82^, ^83^, ^159^ ^]^ To test the OFF‐state expression level of THS, a decoy RNA with a strong secondary structure was used as a control. Similarly, non‐target sRNAs where 15‐nt target binding sites were deleted were used to test conditions without sRNA control (Tables S1–S4, Supporting Information).

This SUPER design was then implemented to target the engineered THS that harbor the sRNA target site within the hairpin loop domain to test whether the sRNA expression could effectively suppress the GFP expression downstream of an activated THS. Strong GFP expression was observed for the activated THS in the presence of the cognate trigger RNA, whereas the GFP expression downstream of THS was almost indistinguishable from an OFF‐state THS when the cognate trigger RNA and synthetic sRNA were co‐expressed (Figure 2b,c). For sRNAs with modified sequences, the strong repression on their cognate THS was maintained (Figure 2d; Figure S3, Tables S5 and S6, Supporting Information). The sRNA‐mediated repression remained stable for up to 24 h, achieving at least 151.3‐fold repression when compared to the ON‐state THS (Figure 2e). Quantitative PCR analysis on mRNA levels showed only a slight change in mRNA level depending on the sRNA expression status, indicating that sRNA‐mediated repression could be largely explained by translational inhibition rather than mRNA decay (Figure S4, Supporting Information). Further, sRNA‐mediated repression remained effective even when the sRNA was expressed with 2 h 30 min delay after THS activation (Figure S5, Supporting Information).

To verify the sequence specificity of synthetic sRNAs for potential extension of this design strategy for general applications, we designed a 5×5 orthogonal sRNA pool by optimizing all the combinatorial pairwise sRNA‐target interactions in silico using NUPACK (Tables S4 and S5, Supporting Information). The GFP fluorescence measurements for all THS target and sRNA combinations showed that the sRNA pool exhibited high sequence specificity with little crosstalk. On‐target repression achieved an average of 1035.1‐fold and a minimum of 766.7‐fold, while off‐target repression showed an average of 2.3‐fold and a maximum of 4.2‐fold (Figure 2f; Figure S6, Supporting Information). In addition, SUPER design strategy was effectively applied to multiple other riboregulator designs, including STAR^[^ ^145^, ^160^ ^]^ and 3WJ repressor ^[^ ^100^ ^]^ (Figures S7 and S8, and Table S7, Supporting Information).

**Figure 3:**
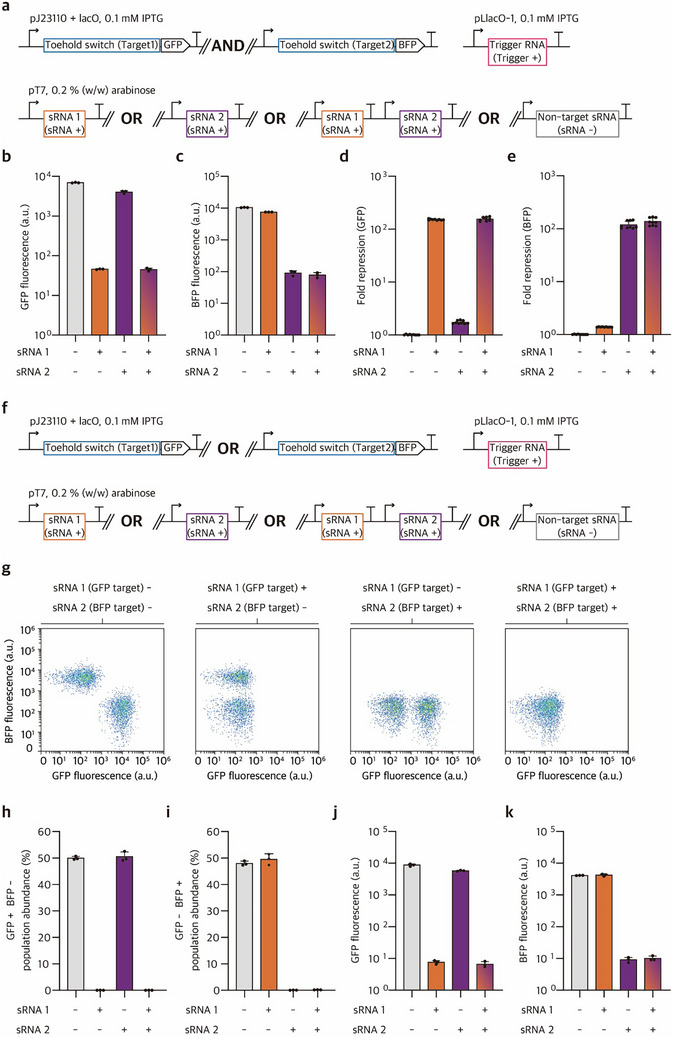
Characterization of orthogonal sRNA‐mediated regulation in dual‐gene expression and microbial co‐culture systems. a) Schematic of dual toehold switch (THS) design for independent regulation of two genes. Two THS (Target1‐GFP and Target2‐BFP) are expressed from a strong constitutive promoter pJ23110 with lacO for IPTG‐inducible expression, while their cognate triggers are expressed from the IPTG‐inducible promoter pLlacO‐1. The dual‐THS system was implemented in a single *E. coli* BL21‐AI cell, and the expression of THS and trigger RNAs was induced with 0.1 mм IPTG. The sRNAs are controlled by the T7 promoter, which is activated by 0.2% (w/w) L‐arabinose (ara) in *E. coli* BL21‐AI, a strain harboring a genomic copy of T7 RNA polymerase under the pBAD promoter. b‐e) Characterization of orthogonal sRNA‐mediated regulation of dual‐gene expression in single cells. GFP fluorescence (b), BFP fluorescence (c), and fold repression for GFP (d) and BFP (e) outputs in the presence and absence of sRNA 1 and sRNA 2. f) Schematic of co‐culture setting for two cell populations, each harboring THS (Target1‐GFP) or THS (Target2‐BFP). Trigger RNA is expressed from pLlacO‐1 and induced with 0.1 mм IPTG. g) Flow cytometry scatter plots showing GFP and BFP fluorescence distributions under co‐culture conditions across four scenarios: both sRNAs absent, sRNA 1 only (GFP target), sRNA 2 only (BFP target), and both sRNAs present. h‐i) Quantification of single‐positive population abundance in co‐culture: GFP+/BFP– (h) and GFP–/BFP+ (i). j‐k) GFP (j) and BFP (k) fluorescence intensity measurements in co‐culture across all four sRNA conditions. Fluorescence was measured by flow cytometry 4 h 30 min after induction. sRNA (+) indicates the presence of a targeting sRNA, while sRNA (–) indicates the absence of a targeting sRNA, with double negatives (–/–) indicating non‐targeting sRNAs only as a negative control. Biological replicates are three. Error bars represent the standard deviation of three biological replicate measurements.

To further explore the modularity and scalability of SUPER, we implemented multiple orthogonal sRNA‐THS modules both within a single cell and across distinct cell populations. First, two THS constructs regulating GFP and BFP were co‐expressed in the same cell, each harboring an sRNA target site specific to sRNA 1 or sRNA 2, respectively (**Figure** **3**a; Figure S9a–c, Supporting Information). Upon sRNA 1 induction, GFP expression was repressed by 151.6‐fold, with a slight 1.4‐fold repression of BFP, whereas expression of sRNA 2 led to a 118.5‐fold repression of BFP, with a modest 1.7‐fold repression of GFP (Figure 3b‐e). Simultaneous expression of both sRNAs could suppress both fluorescence signal outputs, demonstrating the operation of multiple SUPER modules with minimal crosstalk in the same cell. Second, to test orthogonal regulation by SUPER in a mixed population, we prepared two distinct cell populations, one expressing THS‐GFP and the other expressing THS‐BFP (Figure 3f; Figure S9d–g, Supporting Information). Flow cytometry analysis showed that sRNA 1 selectively repressed the gene expression for the targeted GFP‐expressing population, sRNA 2 for the BFP‐expressing population, and co‐expression of both sRNAs could suppress gene expression for both subpopulations (Figure 3g‐k; Figure S9h,i, Supporting Information). These results highlight the orthogonality and scalability of SUPER framework, demonstrating that multiple sRNA‐based regulatory modules can function independently within a single cell or across cell populations.

Taken together, the SUPER design approach was successfully applied to a number of synthetic RNA devices with good performance, robustness, and specificity. Moreover, the strength of sRNA‐mediated repression was correlated with sRNA expression levels (Figure S1d, Supporting Information), consistent with previous reports,^[^ ^81^, ^83^, ^151^ ^]^ indicating that the dynamic range of target riboregulators could be further modulated by adjusting the SUPER system.

### 2.2 SUPER Enables Leakage Reduction and Performance Enhancement

**Figure 4:**
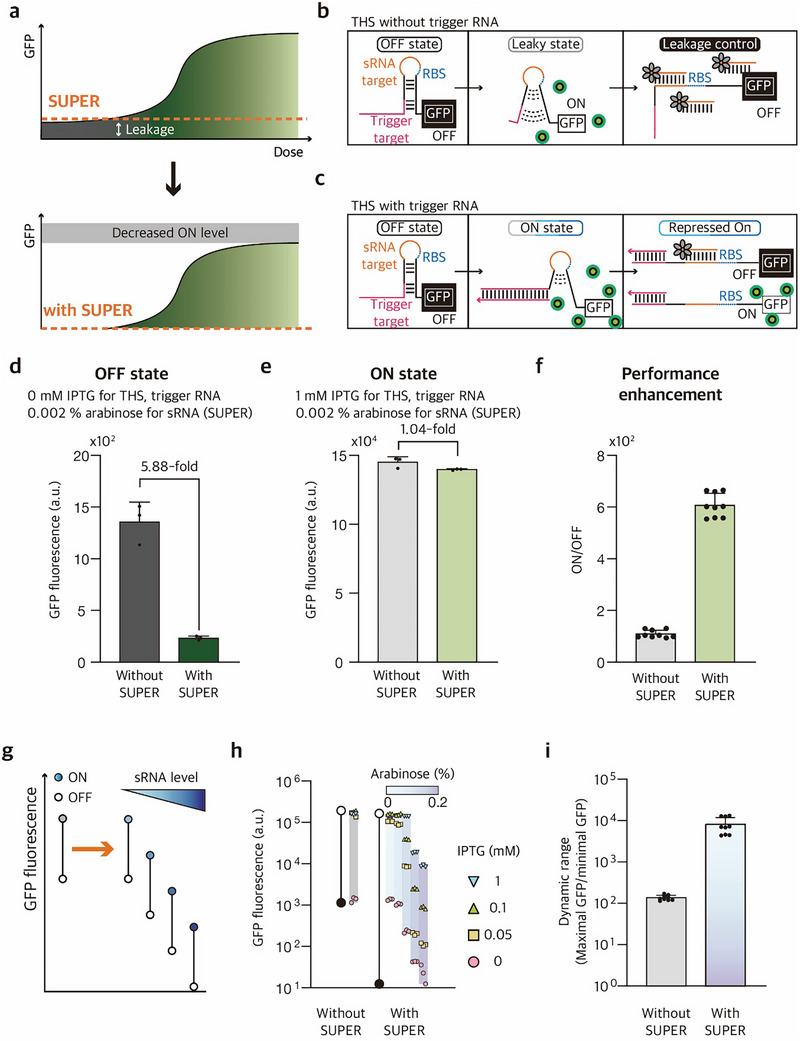
Schematic of SUPER and performance enhancement of THS with SUPER. a) Schematic of leakage reduction and performance enhancement using the SUPER system. b) The OFF‐state THS with SUPER. Even in the absence of cognate trigger, a small fraction of switch RNAs may spontaneously unwind their hairpin structure, leading to leaky expression of downstream genes, which can be effectively sequestered by sRNA with host‐encoded Hfq. c) The ON‐state THS with SUPER. In the presence of cognate trigger, switch RNAs undergo structural changes upon trigger RNA binding to expose RBS, where some fraction of THS with open RBS could be repressed by sRNA. The ON‐state THS without sRNA binding can still express downstream genes. d‐f) Characterization of THS in the presence and absence of SUPER. In the presence of SUPER, a strong leakage reduction (d), a slight reduction in the ON‐state expression (e), and overall enhancement of the ON/OFF fold change (f) were observed. Switch RNA is expressed from a strong constitutive promoter pJ23110, and cognate trigger is expressed from the IPTG‐inducible promoter pLlacO‐1. sRNA is under the control of the T7 promoter, which in turn is induced by L‐arabinose (ara) in *E. coli* BL21‐AI strain with a genomic copy of T7 RNA polymerase under a pBAD promoter. The OFF‐state THS corresponds to no IPTG induction, and the ON‐state THS corresponds to induction with 1 mм IPTG. The condition marked “with SUPER” indicates sRNA expression induced with 0.002% ara. g) Schematic of expression range modulation using the SUPER system. h) Tunable control of the expression ranges for THS via SUPER. The operating range for THS is adjusted in response to the sRNA expression levels. IPTG upregulates the expression of cognate trigger RNA, and ara upregulates the expression of sRNA. The increased sRNA expression could modulate both the maximum and minimum output expression levels from THS. The highest overall GFP fluorescence is marked with a white circle, and the lowest with a black circle. i) The dynamic range for THS with and without SUPER is defined as the ratio of maximal to minimal GFP expression values measured across all tested combinatorial conditions. Fluorescence of cells was measured via flow cytometry 4 h 30 min after induction. The number of biological replicates for the GFP fluorescence measurements is three, and the fold reduction is calculated as the average across all 9 combinations of 3 activated samples (Trigger +, sRNA –) and 3 repressed samples (Trigger +, sRNA +). Error bars are the standard deviation of three biological replicate measurements (d, e), and 9 possible fold reductions (f, i).

When maximally expressed, the SUPER can effectively suppress the target THS expression by more than 1000‐fold (Figure 2), indicating a large room for control when the expression level of SUPER is finely tuned. As a general mechanism to suppress gene expression, the expression of sRNA would necessarily reduce both the ON‐state and OFF‐state expressions by functioning as a synthetic threshold for gene expression (**Figure** **4**a). While sRNA incorporation would likely reduce both ON‐ and OFF‐state expressions, the ON‐state may be suppressed even more (in terms of concentration) due to the presumed increased accessibility of the open loop regions (Figure 4b,c). Nevertheless, there may be a window of opportunity to tune the sRNA expression level to achieve a limited reduction in the ON‐state expression while effectively suppressing the OFF‐state leakage. To experimentally test this approach, we gradually induced sRNA expression levels and modulated THS activity by adjusting inducer concentrations – L‐arabinose for sRNA and isopropyl β‐D‐1‐thiogalactopyranoside (IPTG) for trigger RNA, respectively (Table S8, Supporting Information).

In the absence of SUPER, the tested THS exhibited 108.8‐fold activation with an OFF state leakage (Figure 4d,e). With the SUPER design in place, the OFF‐state leakage gradually decreased with increasing sRNA levels as expected. However, excess sRNA levels also reduced fold activation by strongly suppressing ON‐state expression (Figure S10, Supporting Information). Under suitable expression conditions for optimal dynamic range with SUPER, the OFF‐state leakage level was reduced by 5.88‐fold compared to THS only (Figure 4d), while the ON‐state showed a modest 1.04‐fold reduction (Figure 4e). Consequently, the fold change was improved from 108.8‐fold without SUPER to 606.6‐fold with SUPER (Figure 4f).

In addition, the tunable range of expression levels for a given riboregulator can be greatly expanded by the introduction of SUPER (Figure 4g). By simultaneously controlling the expression levels of SUPER and THS, the dual control system could achieve the GFP expression as low as autofluorescence level, when minimal expression of THS and trigger was combined with maximal expression of sRNA. Conversely, the maximum ON‐state level was achieved when maximal expression of THS and trigger was combined with minimal expression of sRNA (Figure 4h). Together, the expression level of the combined system can be tuned to cover a dynamic range of up to 8115.6‐fold (Figure 4i).

### 2.3 SUPER Enhances Multi‐Layer Cascade Circuits By Suppressing Leakage Propagation

To further evaluate whether SUPER can function in more complex contexts, such as regulatory cascades, we constructed a three‐layer cascade integrating LuxR and ECF11 σ factor. Cascade architectures inherently amplify upstream fluctuations, where even minor leakage in early layers propagates and magnifies downstream, compromising circuit fidelity.^[^ ^161^, ^162^ ^]^ Thus, regulatory cascades serve as a stringent testbed to assess the robustness of SUPER in multi‐layered circuit contexts.

**Figure 5:**
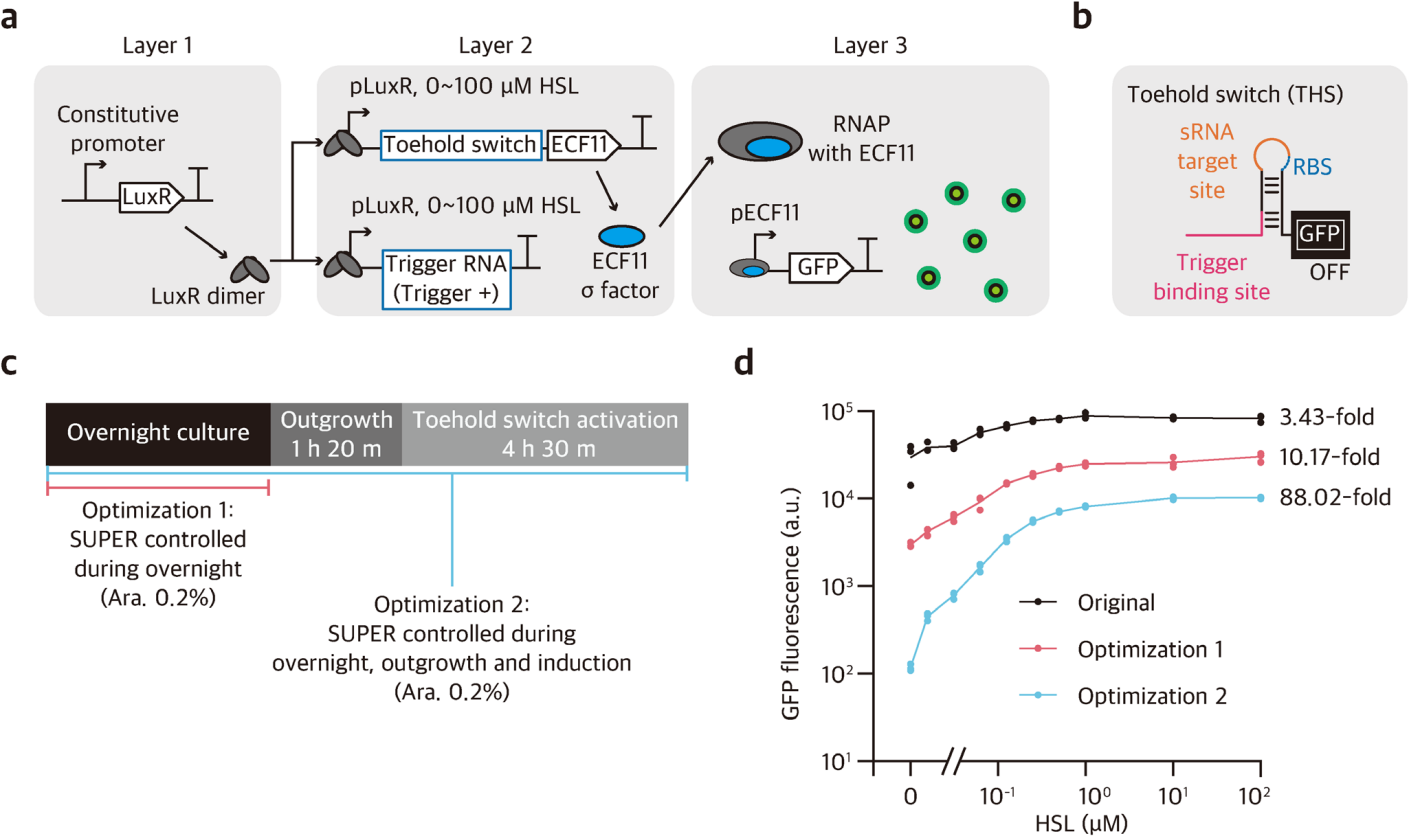
Optimization of multi‐layer cascade circuit with SUPER. a) Schematic of three‐layer gene circuit design. Layer 1: Constitutive expression of LuxR transcription factor. Layer 2: HSL‐inducible expression of toehold switch (THS) and trigger RNA (Trigger +) via pLuxR promoter to control production of ECF11 σ factor. Layer 3: ECF11 σ factor‐dependent GFP expression from pECF11 promoter. b) Structure of THS with sRNA target site (orange) and trigger binding site (pink). c) SUPER optimization strategies for overnight and experimental culture conditions. sRNA is under the control of the T7 promoter, which in turn is induced by L‐arabinose (ara) in *E. coli* BL21‐AI strain with a genomic copy of T7 RNA polymerase under a pBAD promoter. Optimization 1: sRNA induced with 0.2% (w/w) ara during overnight culture. Optimization 2: sRNA induced with 0.2% (w/w) ara during overnight culture, outgrowth (1 h 20 min), and THS activation (4 h 30 min). d) Dose‐response curves of GFP fluorescence across HSL concentrations (0–100 µM) for cascade circuits with and without SUPER control. Fluorescence of cells was measured via flow cytometry 4 h 30 min after HSL induction. The number of biological replicates is three. Error bars represent the standard deviation of three biological replicate measurements.

In this three‐layer cascade, LuxR was constitutively expressed at the top layer (Layer 1), with the second layer (Layer 2) consisting of a pLux‐driven module encoding both the THS–ECF11 mRNA and its cognate trigger RNA, where the ECF11 signal is further relayed to activate expression of GFP reporter in the final layer (Layer 3) (**Figure** **5**a). Upon induction by HSL input, LuxR activates pLux to drive transcription of THS–ECF11 and the trigger RNA, thereby leading to expression ECF11 σ factor. The expressed ECF11 σ factor, in turn, activates the GFP reporter under the pECF11 promoter as the final cascade output. Of note, the THS module in the second layer was designed to be compatible with SUPER‐mediated control to minimize leakage (Figure 5b). In the absence of SUPER‐mediated control, the cascade exhibited substantial GFP reporter expression without HSL input, yielding a modest ON/OFF ratio of 3.43‐fold, presumably due to leaky ECF11 expression in the second layer. To mitigate this, we implemented two different optimization strategies: (i) SUPER expression was induced only during overnight culture to suppress basal ECF11 accumulation prior to experiment (optimization 1), and (ii) SUPER expression was maintained throughout the overnight culture as well as during experimental culture conditions (optimization 2) (Figure 5c). The ON/OFF ratios from regulatory cascades integrated with SUPER improved to 10.17‐fold (optimization 1) and 88.02‐fold (optimization 2), corresponding to a maximum 25‐fold enhancement compared to the cascade without SUPER (Figure 5d). This result indicates that SUPER can be applied to intermediate layers within regulatory cascades, thereby effectively preventing signal leakage to enhance function within complex circuit architecture. Together, these results demonstrate that SUPER can be used to enhance multiple riboregulators and achieve robust control for multi‐layer cascade circuits.

### 2.4 SUPER Enables Performance Enhancement of Riboswitches and RNA Thermometer Without Sequence Alteration

Encouraged by the performance enhancement in multiple riboregulators and multi‐layered circuits, we next examined whether SUPER design could be generalized to other RNA‐based devices. To this end, we applied SUPER design to a number of riboregulators with distinct activation mechanisms, including chemical‐, temperature‐, and protein‐responsive systems.

**Figure 6:**
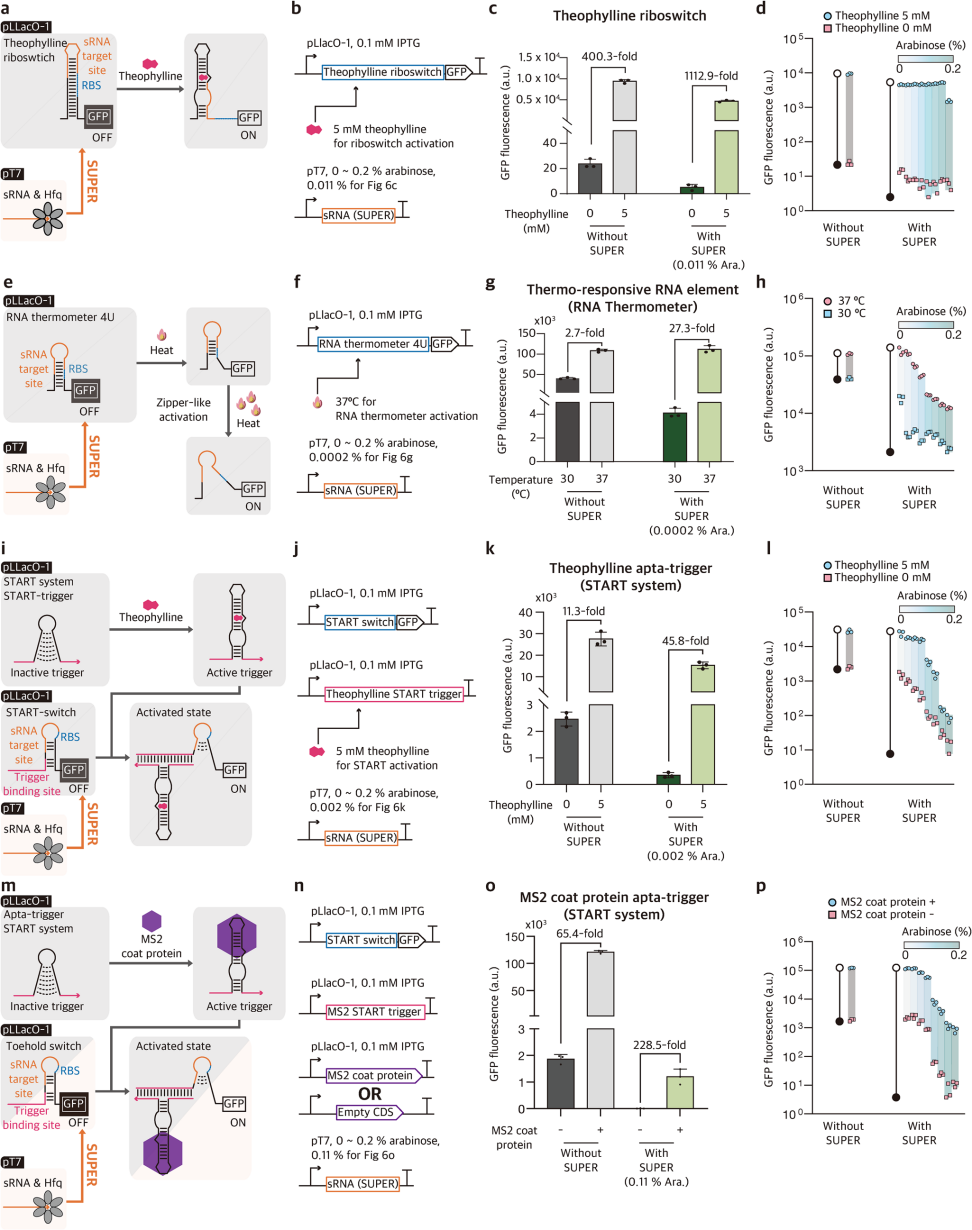
Performance enhancement of riboregulators with SUPER. a) Schematic of theophylline riboswitch. b) Expression cassettes for theophylline riboswitch and cognate sRNA. c) Characterization of GFP expression from theophylline riboswitch in the presence and absence of SUPER. d) Tunable expression range of theophylline riboswitch output in the presence and absence of SUPER. e) Schematic of *Salmonella* 4U RNA thermometer. f) Expression cassettes for *Salmonella* 4U RNA thermometer and cognate sRNA. g) Characterization of GFP expression from 4U RNA thermometer in the presence and absence of SUPER. h) Tunable expression range of 4U RNA thermometer output in the presence and absence of SUPER. i) Schematic of theophylline START system. j) Expression cassettes for theophylline START and cognate sRNA. k) Characterization of GFP expression from theophylline START in the presence and absence of SUPER. l) Tunable expression range of theophylline START output in the presence and absence of SUPER. m) Schematic of MS2 coat protein START system. n) Expression cassettes for the MS2 coat protein START and cognate sRNA. o) Characterization of GFP expression from MS2 coat protein START in the presence and absence of SUPER. p) Tunable expression range of MS2 coat protein START output in the presence and absence of SUPER. Theophylline riboswitch, 4U RNA thermometer, and START switch RNA and trigger RNA are expressed from the IPTG‐inducible promoter pLlacO‐1 and induced with 0.1 mм IPTG, using the original reported sequences without modification.^[^ ^71^, ^146^, ^147^ ^]^ The ON‐ and OFF‐states are regulated by the input signals, theophylline or temperature. MS2 coat protein is expressed from pLlacO‐1, and the absence of MS2 input is implemented using an empty vector, denoted as “–”. sRNA is under the control of T7 promoter, which in turn is induced by L‐arabinose (ara) in *E. coli* BL21‐AI that harbors a genomic copy of T7 RNA polymerase under the pBAD promoter. To control the expression levels of sRNA, ara concentration was adjusted to 0, 0.0002, 0.0011, 0.002, 0.011, 0.02, 0.11, or 0.2% (w/w). For each device, the overall maximum GFP fluorescence expression is marked with a white circle, and the overall minimum output with a black circle in (d, h, l, p). Fluorescence of cells was measured via flow cytometry 4 h 30 min after induction. The number of biological replicates for the GFP fluorescence measurements is three, and the fold change is calculated as the average across all possible combinations between groups, resulting in nine cases. Error bars are standard deviation of three biological replicate measurements.

First, we applied SUPER to a well‐characterized theophylline riboswitch.^[^ ^146^ ^]^ The theophylline riboswitch controls translation initiation by altering mRNA conformation in response to its target ligand, theophylline (**Figure** **6**a). The SUPER design was used to target the theophylline riboswitch, where the sRNA was designed to target regions immediately upstream of RBS within the reported riboswitch, and the theophylline riboswitch was used without modification (Figure 6b). When tested alone, the theophylline riboswitch exhibited 400.3‐fold activation upon the introduction of theophylline. When SUPER design was applied together with theophylline riboswitch, reduced leakage expression and increased fold change were observed (Figure S11, Supporting Information). With an optimized sRNA expression level, the fold change was increased, reaching up to 1112.9‐fold (Figure 6c). In addition, the expression levels of the theophylline riboswitch could be further modulated by adjusting SUPER control (Figure 6d).

Second, the *Salmonella* 4U RNA thermometer was chosen as a target for SUPER design.^[^ ^147^ ^]^ The RNA thermometer possesses a stable secondary structure that blocks ribosome access to RBS, which is relieved when the temperature increases due to the lower stability of secondary structure at elevated temperature (Figure 6e). Analogously, sRNA was designed to target the domain immediately upstream of RBS in the 4U RNA thermometer (Figure 6f). The 4U RNA thermometer, when tested alone, showed a modest fold change of 2.7‐fold upon temperature increase from 30 to 37 °C. With an optimized sRNA level, however, the temperature‐dependent activation was enhanced to 27.3‐fold (Figure 6g; Figure S12a–c, Supporting Information). The resulting enhanced dynamic range of the RNA thermometer with SUPER was comparable to temperature‐dependent transcription factor‐based systems (Figure S12d–g, Supporting Information).^[^ ^148^ ^]^ Further, the tunable range of the 4U RNA thermometer outputs could be expanded by SUPER (Figure 6h).

Third, SUPER design was used to target a recently reported START (Synthetic Trans‐Acting Riboswitch with Triggering RNA) system, a potential alternative to conventional cis‐acting riboswitches.^[^ ^71^ ^]^ By separating the riboswitch function into two RNA species, i.e., START trigger responsible for ligand sensing and START switch that controls gene expression, START system aimed to achieve improved design flexibility for riboswitches. In the same vein, we applied SUPER designs to the theophylline‐responsive (Figure 6i,j) and MS2‐responsive (Figure 6m,n) START systems. The results showed that the application of SUPER designs to START systems improved fold changes much like other riboswitch systems. The fold change was improved from 11.3‐fold to 45.8‐fold for the theophylline‐responsive START (Figure 6k; Figure S13, Supporting Information), and from 65.4‐fold to 228.5‐fold for the MS2‐responsive START (Figure 6o; Figure S14 and Table S10, Supporting Information). Similarly, expanded tunable output ranges were observed as the SUPER expression levels were tuned (Figure 6l,p).

Together, these results suggest that SUPER can serve as an effective strategy for natural and synthetic RNA devices, even without any target sequence modifications in certain cases. Despite a number of differences in the sequence contexts and predicted structures of RNA devices tested, SUPER could enhance the dynamic ranges and tune the expression levels for the target riboregulators. This indicates that the SUPER design may be potentially applicable to a wide variety of RNA‐based genetic circuit architectures such as biocontainment of synthetic biological devices for live biotherapeutics and environmental monitoring.^[^ ^163^, ^164^, ^165^, ^166^ ^]^ The ability to precisely tune the expression levels and minimize leaky expression could be explored under stringent conditions required for biocontainment where it is of critical importance to maintain genetic stability under a strong selection pressure.^[^ ^107^, ^112^, ^113^, ^114^ ^]^

### 2.5 SUPER Enables Sophisticated Regulation of a Holin‐Based Kill Switch For Long‐Term Genetic Stability

**Figure 7:**
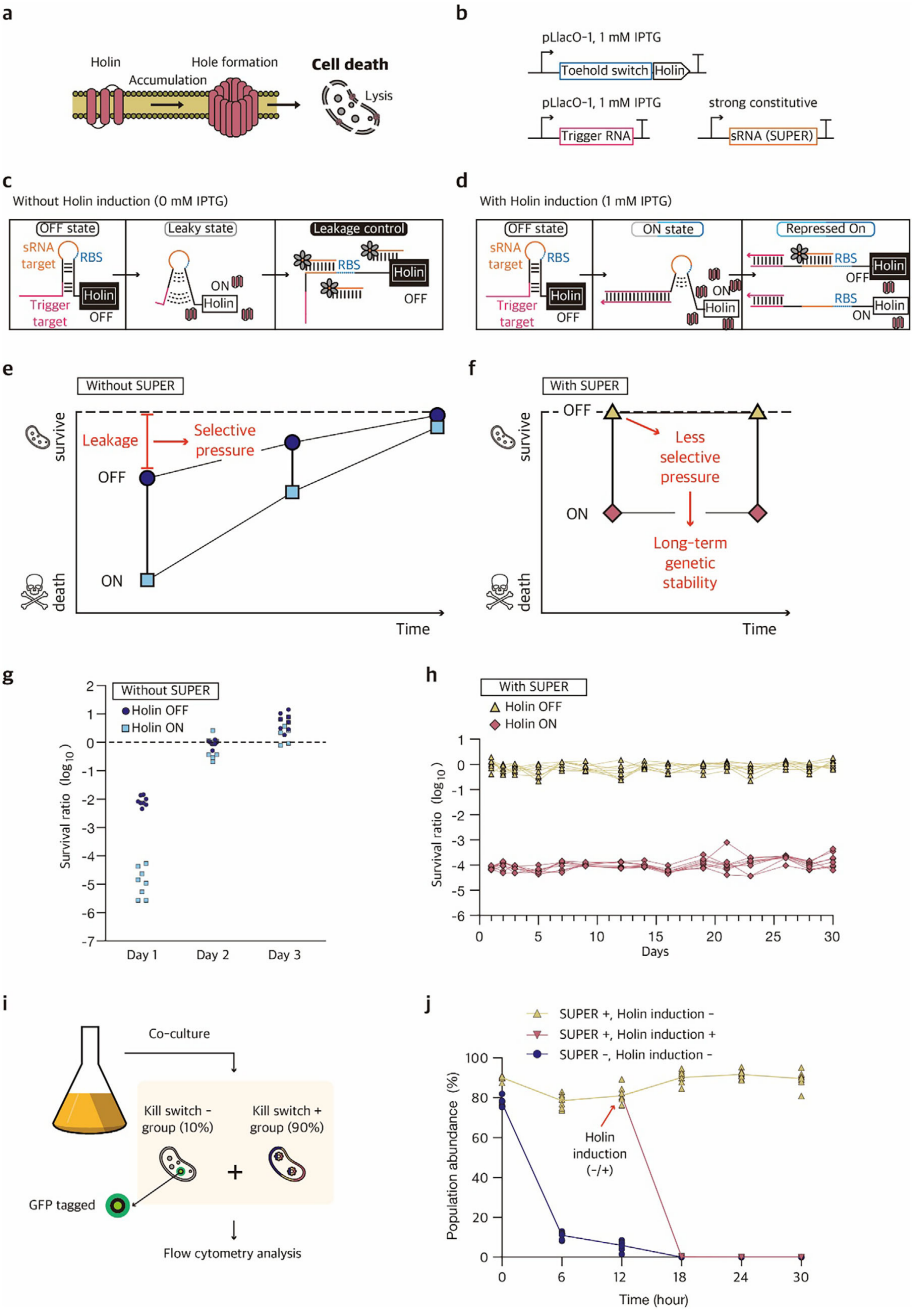
Long‐term genetic stability of a kill switch integrated with SUPER. a) Schematic of Holin‐based lysis circuit. The Holin circuit, derived from the lysis mechanism of bacteriophage λ, consists of three components: S105, R, and Rz. b) Expression cassettes for the Holin‐based kill switch with SUPER. Holin‐encoding THS and trigger RNAs are expressed from the IPTG‐inducible promoter pLlacO‐1, and sRNA is transcribed from a strong constitutive promoter in *E. coli* DH5α strain. c) Schematic of leakage control of Holin‐based kill switch circuit with SUPER. d) Schematic of activation of Holin‐based kill switch circuit with SUPER. e) The impact of leaky expression of toxin in the kill switch circuit. Leaky expression of Holin in the OFF‐state kill switch can compromise cell viability, which in turn imposes a selection pressure that could lead to the emergence of escapees carrying non‐functional kill switch circuits. f) Precise control of toxic output via SUPER for long‐term genetic stability. The reduction of leaky expression of toxin via SUPER reduces the selective pressures on cells, supporting the long‐term stability of synthetic kill switch circuits. g) Colony forming unit (CFU) assay for kill switch‐harboring cells without SUPER during a three‐day subculture. After each overnight culture, cells were induced with 0 mм (Holin OFF) or 1 mм (Holin ON) IPTG for 4 h before CFU measurements. h) CFU assay for kill switch‐harboring cells with SUPER during a thirty‐day subculture. After each overnight culture, cells were induced with 0 mм (Holin OFF) or 1 mм (Holin ON) IPTG for 4 h before CFU measurements. The number of biological replicates was eight for (g, h). i) Schematic of the experimental design for a kill switch‐harboring strain in bacterial co‐culture conditions. j) Flow cytometry analysis to measure relative population abundance over time. The kill switch‐harboring strain (kill switch +) and a healthy control without a kill switch circuit (kill switch –) were mixed at a 9:1 ratio initially. Sampling was conducted every 6 h up to 30 h. At 12 h, IPTG was introduced to one of the subculture conditions to test the functional response of the kill switch circuit. The number of biological replicates was eight for (j).

For biocontainment of engineered hosts, synthetic kill switch designs have been explored that harbor artificial gene circuits designed to induce cell death under specific conditions.^[^ ^107^, ^112^, ^113^, ^114^, ^167^, ^168^ ^]^ Since these kill switches control the survival of engineered hosts, those that escape the control could acquire growth advantages. Thereby, any growth defect in the hosts due to leaky expression could result in compromised stability of synthetic gene circuits.^[^ ^107^, ^112^, ^113^, ^114^ ^]^ Taking advantage of the stringent control of leaky expression by SUPER, we aimed to combine the SUPER with synthetic kill switches to improve the performance and genetic stability. We applied the SUPER design together with synthetic kill switch containing Holin, a lysis‐inducing toxin, under the control of THS (**Figure** **7**a).^[^ ^169^, ^170^ ^]^ In this design, IPTG signal induces the expression of Holin‐coding sequence under THS and trigger RNA, whereas the sRNA was transcribed from a strong constitutive promoter (Figure 7b–d; Table S11, Supporting Information). The functionality of synthetic kill switch could be evaluated by measuring growth suppression in the presence and absence of IPTG signal over an extended period of time. Any growth suppression in the absence of IPTG signal reflects system leakage, and lack of growth suppression in the presence of IPTG signal indicates the loss of functional gene circuits (Figure 7e). On the other hand, a stringent leakage control may allow reproducible response for an extended period of time, where the population harboring kill switch does not show growth reduction in the absence of kill signal, whereas a strong reduction in the population could be observed in the presence of kill signal after many generations (Figure 7f). To minimize recombination‐driven instability, we employed *E. coli* DH5α, a recA‐deficient strain that provides enhanced genetic stability for synthetic kill switch circuits.^[^ ^112^, ^171^ ^]^

Preliminary tests indicated that the population with kill switch quickly lost the ability to respond to IPTG signal due to system leakage and low genetic stability, whereas the kill switch remained responsive to IPTG signal when combined with SUPER control (Figure S15a,b, Supporting Information). The functionality of synthetic kill switches was evaluated for an extended period of time where the population was subcultured on a daily basis for up to 25 days, where the cell growth was monitored by OD600, the absorbance at 600 nm that serves as a proxy for culture density. The population with kill switch in the absence of SUPER lost the response to IPTG signal overnight, whereas the population with kill switch and SUPER control maintained functional response up to 25 days with the exception of a sole escapee observed on day 19 (Figure S15c,d, Supporting Information). The analysis of the Holin‐coding region revealed that all escapees carried genetic mutations, including transposable element insertions. Among these, 19 out of 24 mutations in the SUPER negative group and 1 out of 1 mutation in the SUPER positive group were transposable element insertions (Figures S16 and S17, Supporting Information).^[^ ^112^ ^]^

To better understand the sources of leakage in the kill switch design, we constructed an alternative design using a decoy RNA in place of the cognate trigger RNA. This alternative construct could help distinguish transcriptional leakage (from pLlacO promoter) from translational leakage (through THS). The results revealed that both regulatory layers contribute substantially to kill switch activation, where transcriptional leakage from the alternative architecture led to intermediate cell survival (Figure S18, Supporting Information). Without SUPER, severe toxicity was observed across all conditions (survival ratios of 10^−2^–10^−6^), demonstrating uncontrolled leakage at both transcriptional and translational levels. While SUPER could improve survival to the healthy control level under fully repressed conditions, substantial cell death still occurred in case transcription of THS‐Holin was activated. This observation underscores that achieving robust biocontainment requires stringent control at multiple regulatory layers, as leakage at even a single step can compromise system stability.

Encouraged by this, we aimed to quantitatively evaluate the kill switch functionality for an extended period of time via colony forming unit (CFU) assays. For the population with kill switch in the absence of SUPER control, the cell survival ratio (the CFU of kill switch‐harboring cells divided by the CFU of cells without Holin‐coding region) diminished by 127.1‐fold even under permissive condition without IPTG signal, indicating system leakage. This population lost the ability to respond to IPTG signal by day 2 (Figure 7g; Figures S18 and S19, Supporting Information). In contrast, the population with kill switch integrated with SUPER control exhibited an average 1.45‐fold reduction in survival ratio during the continuous daily subculture for a 30‐day period, under permissive conditions without IPTG signal. Yet, the population maintained the functional response to IPTG signal up to 30 days, where a portion of population was subject to IPTG signal to test functionality of synthetic kill switches. This population responded with an average 9490.3‐fold reduction in the presence of IPTG (Figure 7h; Figure S19, Supporting Information).

The SUPER control provides a promising solution to address one of the key challenges in synthetic kill switch designs by providing stringent leakage suppression, allowing for long‐term genetic stability. Still, the performance of synthetic controllers can be critically influenced by the presence of competing populations, where a slight leakage‐driven fitness cost can lead to rapid loss of synthetic circuit functionality under competition with faster‐growing cells without the burden. This cellular population dynamics becomes particularly important in scenarios where engineered strains may encounter wild‐type or mutant competitors, whether through intentional co‐culture applications in metabolic engineering or through unintended contamination or evolutionary escape in biocontainment contexts. Therefore, we next aimed to evaluate the performance of the SUPER‐based kill switch in a co‐culture setting.

To investigate whether synthetic kill switch under SUPER control could maintain functionality without growth disadvantages in microbial co‐culture conditions, we prepared a mixed culture where the populations harboring synthetic kill switches and healthy controls are differentially labeled. As a preliminary step, we assessed the effect of GFP labeling on population dynamics by co‐culturing control strains with and without GFP at a 9:1 ratio. Over 48 h, the population ratio remained stable (Figure S20, Supporting Information). After confirming that GFP labels do not cause substantial burden on cells, the cell population that harbors kill switch circuits without GFP labels (GFP–) and the control population without Holin‐coding region that contain GFP labels (GFP+) were co‐cultured at a 9:1 ratio, and the population levels were tracked via flow cytometry (Figure 7i). The population abundance of cells that harbor kill switch circuits sharply decayed to 11.0% by 6 h in the absence of integrated SUPER (Figure 7j, navy line), whereas the abundance of kill switch population remained stable for up to 30 h with SUPER control (Figure 7j, yellow line). The functional response of the kill switch circuit was confirmed upon IPTG induction at 12 h, with population abundance decreasing to an average of 0.2% within 6 h post‐induction (Figure 7j, red line). The population dynamics remained consistent even when the initial seeding ratio of kill switch population and healthy control was adjusted (Figure S21, Supporting Information). Together, the tight leakage control by SUPER allows the stable maintenance of kill switch circuits in a microbial co‐culture setting.

### 2.6 A 2‐Input Kill Switch That Combines Chemical and Environmental Signals

**Figure 8:**
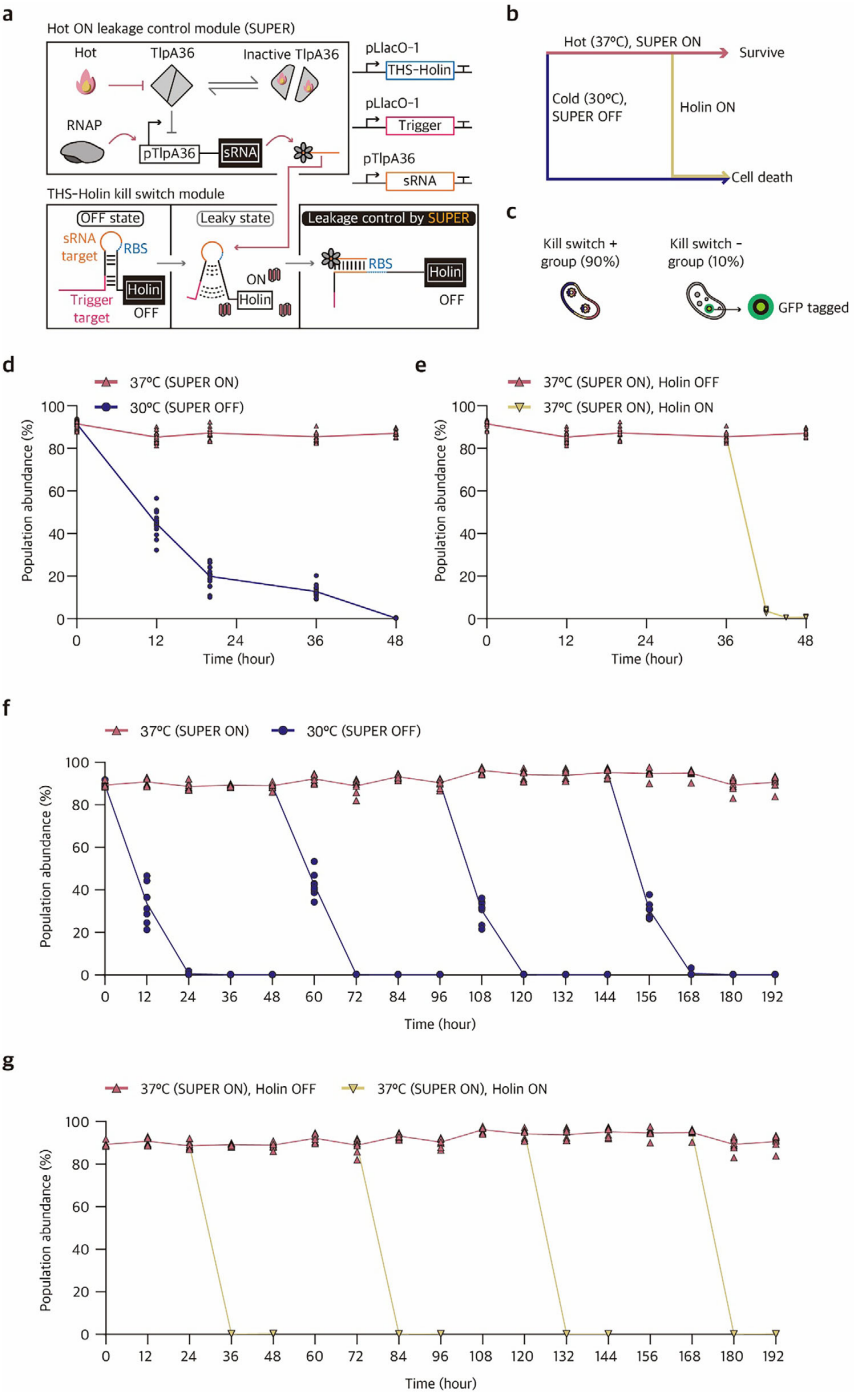
A 2‐input kill switch using temperature‐responsive SUPER design. a) Schematic of chemical‐ and temperature‐responsive 2‐input kill switch. At 37 °C, SUPER is activated via temperature‐sensitive TlpA36‐driven promoter and reduces the leaky expression from the kill switch circuit. At 30 °C, however, SUPER is deactivated, and the leaky expression of Holin from the OFF‐state kill switch could lead to reduced cell viability. b) Diagram for the expected response of 2‐input kill switch for different input conditions. c) Differential labeling of cells with kill switch circuit (GFP–) and healthy controls without kill switch circuit (GFP+). d) Changes in the relative population abundance of cells with a 2‐input kill switch in response to temperature. Initially, the kill switch‐harboring group and the healthy control were mixed at a 9:1 ratio. At 37 °C, SUPER can be active and the kill switch population abundance remains stable for 48 h. At 30 °C, however, SUPER is not activated, resulting in the reduction of kill switch‐harboring cells to an average of 0.2% at 48 h. e) Changes in the relative population abundance of cells with a 2‐input kill switch in response to a kill signal, 1 mм IPTG. Initially, the kill switch‐harboring group and the healthy control were mixed at a 9:1 ratio. At 36 h, IPTG, the kill switch activating signal, was introduced to a subculture, resulting in the functional response of the kill switch‐harboring cells where the population abundance decreased to an average of 0.7% at 48 h. The number of biological replicates was sixteen for (d, e). f‐g) Long‐term stability of the 2‐input kill switch under temperature modulation (f) and IPTG induction (g). The number of biological replicates was eight for (f, g).

To construct a temperature‐responsive SUPER system, we first tested the sRNA expression under the control of a temperature‐responsive transcription factor, TlpA36 (Table S12, Supporting Information).^[^ ^148^ ^]^ TlpA36 can form a stable dimer at low temperature (≤30 °C) to block transcription downstream of pTlpA promoter. At high temperature (≥37 °C), however, the TlpA36 dimer dissociates, allowing the expression of downstream sRNA signal (**Figure** **8**a). To validate this temperature‐responsive SUPER design, we first constructed a reporter system where TlpA36‐controlled sRNA regulates GFP expression through THS (Figure S22a, b, Supporting Information). This circuit demonstrated consistent temperature‐dependent gene regulation in multiple strains, achieving 3879.67, 175.40, and 81.37‐fold changes in GFP expression in *E. coli* BL21‐AI, *E. coli* K‐12 MG1655, and *E. coli* Nissle 1917, respectively (Figure S22c–e, Supporting Information). Building upon the enhanced genetic stability and functionality of the kill switch combined with SUPER control, we replaced a constitutive promoter upstream of sRNA in the previous design with the pTlpA promoter for the temperature‐responsive THS‐Holin kill switch. In this design, sRNA downstream of pTlpA promoter can be expressed at 37 °C for tight leakage control of Holin, but the sRNA expression is halted at 30 °C, allowing the leaky expression of Holin. As before, the THS‐Holin and the cognate trigger RNA were under the control of IPTG‐inducible promoters, such that IPTG signal could effectively eliminate the population (Figure 8a,b).

For a stringent test on the genetic stability and functionality of SUPER‐integrated 2‐input kill switch, we performed a microbial co‐culture experiment. In the co‐culture setting, the healthy control was labeled with GFP fluorescence (GFP+) and the population harboring kill switches did not have fluorescence label (GFP−) for population tracking via flow cytometry (Figure 8c). The kill switch population (GFP−) and the healthy control population (GFP+) were co‐cultured at a 9:1 ratio initially, and the populations were subject to SUPER control with low leakage at 37 °C and a non‐permissive condition with some leakage without SUPER control at 30 °C. The abundance of population containing kill switches remained stable for up to 48 h when cultured at 37 °C, whereas it decreased to an average of 0.2% by 48 h in cultures maintained at 30 °C (Figure 8d). To test the functionality of kill switch circuits, IPTG signal was introduced at 36 h for the population maintained under the permissive condition (37 °C), where the population abundance decreased sharply from 85.4% at 36 h to 0.7% at 48 h (Figure 8e).

To further assess the long‐term stability and evolutionary robustness of the 2‐input kill switch, we monitored the composition of cell population and circuit functionality for 8 days under permissive conditions (37 °C, without IPTG). During this period, the population harboring the kill switch remained stable while responding to kill signals via IPTG induction and exposure to 30 °C (Figure 8f, g). Sequencing analysis of samples collected after 8 days showed no detectable genetic mutations, confirming that the 2‐input kill switch maintained both functional integrity and genetic stability during prolonged cultivation (Figure S23, Supporting Information).

Although the 2‐input kill switch demonstrated high stability under permissive conditions, it could still be susceptible to temperature fluctuations, especially for extended period of storage. To test the feasibility of long‐term storage, overnight cultured cells with the 2‐input kill switch were mixed 1:1 with a formulation buffer and snap‐frozen in liquid nitrogen (Figure S24a, Supporting Information).^[^ ^172^ ^]^ The 2‐input kill switch retained circuit functionality after 30 days of storage at −80 °C, with no detectable mutations by sequencing analysis (Figure S24b‐f, Supporting Information, see Supporting Information for detailed methods).

In summary, a 2‐input kill switch that integrates chemical and environmental signals was demonstrated by combining SUPER system with a temperature‐responsive transcription factor, TlpA36. This 2‐input kill switch could be activated by either a chemical signal (IPTG) or a low temperature (30 °C) much like an OR‐logic gate. The 2‐input kill switch integrated with SUPER maintained circuit functionality and genetic stability for at least 8 days under permissive conditions, without any detectable mutations in circuit components. In addition, the engineered cells could be stably preserved at −80 °C for up to 30 days without loss of functionality. Together, these results demonstrate that the SUPER‐integrated 2‐input kill switch could provide a reliable and durable framework for multi‐input biocontainment applications.

## 3 Discussion

In this study, we developed SUPER, a novel genetic parts upcycling platform that leverages synthetic sRNA as a post‐transcriptional antagonist for protein translation. As a starting point, we characterized the performance and properties of sRNA, demonstrating its strong, stable, robust, and target‐specific control over riboregulators. Next, by applying SUPER, we enhanced the dynamic range of RNA‐, theophylline‐, temperature‐, and protein‐responsive RNA devices. To minimize leakage, we integrated SUPER into a Holin‐expressing kill switch, successfully maintaining kill switch activity for up to 30 days. Furthermore, SUPER enabled the sustained viability of kill switch‐harboring bacteria under microbial co‐culture conditions. Finally, by combining sRNA with the temperature‐responsive transcription factor TlpA36, we constructed a chemical‐ and temperature‐responsive 2‐input kill switch.

SUPER has several advantageous features as a framework for upcycling genetic devices. First, SUPER provides a straightforward design process. As demonstrated herein, sRNAs can effectively modulate riboregulators by targeting 15‐nt regions immediately upstream of the RBS, without altering the native 5′ UTR.^[^ ^83^ ^]^ By simplifying the engineering workflow, this unique feature allows its application to a wide variety of RNA‐based control systems.^[^ ^58^, ^82^, ^173^, ^174^, ^175^, ^176^ ^]^ Second, SUPER imposes minimal cellular burden. As an RNA‐based regulatory system, the metabolic load on host cells can be reduced, thereby enhancing compatibility with multiple RNA and protein regulators together with long‐term circuit stability without resorting to additional maintenance systems such as toxin‐antitoxin modules.^[^ ^113^, ^148^, ^177^, ^178^, ^179^, ^180^, ^181^, ^182^ ^]^ Finally, SUPER offers extended tunability, enabling the programmable adjustment of response curves of conventional riboregulators by modulating sRNA expression strength (Figures 4 and 6). This finely tunable architecture could enable an on‐demand optimization of synthetic circuit dynamics, reducing the need for time‐consuming iterative redesign cycles.^[^ ^173^, ^174^, ^176^ ^]^ Collectively, these characteristics make SUPER a valuable tool for enhancing the functionality and control of RNA‐based regulatory systems.

To further develop the SUPER strategy into a versatile platform for complex signal processing, several improvements would be desired. To compensate for the reduction in maximal output ranges due to sRNA‐mediated signal repression, additional signal processing steps would be required. This could potentially be addressed by introducing amplifier circuits to improve the dynamic range and robustness of gene expression.^[^ ^45^, ^65^, ^183^, ^184^, ^185^, ^186^, ^187^, ^188^ ^]^ Another challenge for SUPER as a general regulatory platform is the ability to maintain consistent performance across diverse riboregulator contexts.^[^ ^58^, ^189^, ^190^ ^]^ While our current work employed thermodynamic modeling to predict riboregulator dynamics,^[^ ^80^, ^82^, ^83^, ^153^, ^154^, ^191^ ^]^ adopting kinetic modeling may provide more accurate and reliable predictions, thereby improving design precision.^[^ ^192^, ^193^, ^194^, ^195^, ^196^, ^197^, ^198^ ^]^ Additionally, as the nucleolytic pathways and nucleolytic motifs of sRNAs have been characterized in detail,^[^ ^199^, ^200^, ^201^, ^202^, ^203^ ^]^ incorporating such motifs into SUPER design may further enhance riboregulator functionality and turnover. While not explored further in the present study, capitalizing on the high specificity of sRNA regulators, the 5×5 orthogonal sRNA pool (Figures 2f and 3; Figure S6, Supporting Information) could be expanded to enable concurrent regulation of multiple riboregulators with diverse functionalities.^[^ ^83^, ^204^, ^205^, ^206^ ^]^ This capability would contribute to the creation of a sophisticated signal processing platform capable of integrating diverse inputs for advanced biological computation.^[^ ^58^, ^65^, ^207^ ^]^ While SUPER can be used to modulate complex circuits, the limits on cellular burden and system complexity may need to be carefully balanced. When designing a two‐input kill switch system (Figure 8), we also explored an alternative multi‐layered circuit architecture using the SUPER‐enhanced 4U RNA thermometer to drive sRNA expression via an intermediate transcriptional activator. However, this design exhibited only modest temperature‐dependent regulation compared to the TlpA36‐based system, suggesting that simpler circuit architecture could aid in achieving reliable performance with SUPER.

Extending SUPER to diverse bacterial strains would be a crucial consideration to use SUPER system as broadly deployable platform. To examine this potential, we introduced a temperature‐responsive SUPER circuit into wild‐type strains *E. coli* Nissle 1917 and *E. coli* MG1655 (Figure S22c‐e, Supporting Information). These circuits functioned consistently across strains without any optimization although some differences in dynamic ranges were observed. Recent studies demonstrated functionality of synthetic sRNAs in various bacterial species through alternative Hfq scaffolds and compatible Hfq proteins.^[^ ^79^, ^208^, ^209^, ^210^ ^]^ Taken together, approaches such as developing broad‐host‐range Hfq variants,^[^ ^144^, ^211^, ^212^, ^213^ ^]^ co‐expressing compatible Hfq proteins,^[^ ^79^, ^210^, ^214^ ^]^ and mapping strain‐specific sRNA–mRNA interactions^[^ ^215^, ^216^ ^]^ may enable consistent transfer of SUPER into diverse hosts for metabolic engineering, medical biotechnology, and industrial bioproduction.^[^ ^217^, ^218^, ^219^ ^]^

The integration of SUPER with standardized genetic parts assembly systems could establish a universal framework for genetic part upcycling. Widely adopted assembly platforms such as MoClo, SEVA, and 3G (Golden Gate–Gibson) assembly support hierarchical and combinatorial construction of multigene circuits through modular part libraries.^[^ ^35^, ^220^, ^221^, ^222^, ^223^, ^224^, ^225^ ^]^ SUPER maintains consistent regulatory performance across heterogeneous promoter contexts, as demonstrated by robust repression achieved with the same synthetic sRNA without requiring sequence optimization (Figure S25, Supporting Information). This promoter‐independent functionality supports SUPER's compatibility with diverse transcriptional inputs. In principle, SUPER's components, including sRNA expression cassettes and targeted 5′ UTR elements, could be designed as Level 0 MoClo modules to facilitate combinatorial assembly using existing pipelines, thereby promoting rapid prototyping, community sharing, and automation in high‐throughput screening of sRNA‐based regulatory systems.^[^ ^205^, ^226^ ^]^

In conclusion, we developed SUPER, a systematic engineering framework that could upcycle and modulate a wide range of genetic devices. While RNA‐based regulatory devices offer certain advantages over conventional protein‐based circuits—including compact genetic encoding space, high scalability, and versatile sensing capabilities—limited tunability in dynamic and operational ranges due to the intricate interdependence of performance on sequence contexts has often hampered their integration into complex synthetic circuits. SUPER introduces a robust framework for addressing these key challenges by expanding the dynamic ranges of the current suite of regulators and providing a novel methodology to control leaky expressions, thereby enhancing genetic circuit stability and mitigating escape mechanisms that could compromise in vivo applications. Furthermore, SUPER introduces a systematic approach to fine‐tune the response curves of RNA devices, enabling the tunable adjustment of diverse response profiles within the same set of regulator libraries. This tunability could potentially be used to generate a high‐quality data set for machine learning‐based approaches to synthetic regulator design and optimization. Given that sRNA‐based regulatory systems are prevalent across phylogenetically diverse bacterial species,^[^ ^79^, ^210^, ^227^, ^228^ ^]^ we anticipate that this work could aid in advancing both biotechnology and biological computing through improvements in circuit performance, operational stability, and a rational design framework. Taken together, the SUPER platform could upcycle the existing regulatory devices and facilitate the rational design of controllable RNA‐based circuits, thereby providing support for future advancements in synthetic biology.

## 4 Experimental Section

### Materials, Strains, and Growth Conditions

DNA primers were purchased from Bionics (Seoul, Korea). Reagents used for *E. coli* cultivation were purchased from Formedium (Hunstanton, England, UK). *E. coli* strains DH5α (*endA1 recA1 gyrA96 thi‐1 glnV44 relA1 hsdR17* (r_K_^−^ m_K_^+^) λ^−^) and BL21‐AI (F^−^ *omp*T *hsd*S_B_ (r_B_^−^ m_B_^−^) *gal dcm ara*B::T7RNAP‐*tet*A) were obtained from Enzynomics (Daejeon, Korea) and Invitrogen (Waltham, MA, USA). All strains were grown in Luria‐Bertani (LB) medium at 37 °C with appropriate antibiotics: Kanamycin (30 µg/ml), Ampicillin (50 µg/ml), Spectinomycin (25 µg/ml), and Chloramphenicol (17 µg/ml). Unless otherwise noted, all experiments evaluating riboregulator performance using GFP fluorescence were conducted in *E. coli* BL21‐AI. All plasmid construction and kill switch experiments were performed in *E. coli* DH5α.

### Plasmid Construction

The backbones of the plasmids used in this study were derived from the commercial vectors pET15b (ampicillin resistance, ColE1 origin), pCDFDuet (spectinomycin resistance, CloDF13 origin), pCOLADuet (kanamycin resistance, ColA1 origin), and pACYCDuet (chloramphenicol resistance, p15A origin) from EMD Millipore (Burlington, MA, USA). All sRNA expression cassettes were constructed in pET15b backbone plasmids. All riboregulator expression cassettes were constructed in pCOLADuet. Trigger RNAs and MS2 coat protein expression cassettes were constructed in pCDFDuet and pACYCDuet, respectively. All constructs were cloned by Circular Polymerase Extension Cloning (CPEC)^[^ ^229^ ^]^ and round‐the‐horn site‐directed mutagenesis.^[^ ^230^ ^]^ Plasmid architecture and specific part sequences are listed in Tables S1–12 (Supporting Information). Plasmids were constructed in *E. coli* DH5α and purified using the EZ‐Pure Plasmid Prep Kit. Ver. 2 (Enzynomics, Daejeon, Korea). Plasmid sequences were confirmed by DNA sequencing. Plasmids were transformed in *E. coli* by chemical transformation.

### Cell Culture for GFP Expression

For in vivo GFP expression experiments, we used *E. coli* BL21‐AI with chromosomally integrated T7 RNA polymerase under the control of the L‐arabinose‐inducible pBAD promoter. For the experiments that use *E. coli* BL21‐AI strain, chemically transformed cells were cultured on 1.5% LB agar plates (Formedium, Hunstanton, England, UK) with appropriate antibiotics. Single colonies were grown overnight (∼16 h) in 96‐deep well plates (Cat# 503 102, NEST, Wuxi, Jiangsu, China) with shaking at 800 rpm, 37 °C. Overnight cultures were diluted 1:100 in fresh LB media with appropriate antibiotics and returned to an orbital shaker (800 rpm, 37 °C; Cat# SHLDMP03DG, OHAUS, Parsippany, NJ, USA). After 80 min, cell cultures were induced with 0.1 mм IPTG (Promega, Madison, WI, USA) and/or 0.2% (w/w) L‐arabinose (Gold Biotechnology, St. Louis, MO, USA). Cell cultures were returned to the shaker (800 rpm, 37 °C) until fluorescence measurement after 4 h 30 min. LB media were prepared the day before and stored at 4 °C until the experiment. If passaging was required during the experiment, pre‐warmed LB adjusted to the experimental temperature was used. Detailed induction and culture conditions for each experiment are provided in Table S8 (Supporting Information).

### Flow Cytometry Measurement

GFP fluorescence was measured by flow cytometry (CytoFLEX S, Beckman Coulter, Brea, CA, USA) after fixation at the Microbiome Core Research Support Center of Korea Basic Science Institute (KBSI). The cell pellet was resuspended with 2% (w/v) para‐formaldehyde solution and fixed for 15 min at room temperature. After fixation, samples were washed twice with 1× phosphate buffered saline (PBS), and stored at 4 °C until the flow cytometry analysis. Fixed cells were diluted 1:10 in 1× PBS. Cells were detected using a forward scatter (FSC) trigger, and a minimum of 50 000 events were recorded for each measurement. The cell population was gated according to the FSC and side scatter (SSC) distributions. To evaluate the riboregulator performance, the fluorescence of GFPmut3b‐ASV was measured on a fluorescein isothiocyanate (FITC) channel, excited with a 488‐nm laser and detected with a 525/40‐nm bandpass filter. GFP fluorescence histograms yielded unimodal population distributions, and the geometric mean was employed for calculating population average. For the GFP fluorescence measurements, cell autofluorescence measured by *E. coli* BL21‐AI strain without GFP coding sequence was subtracted. For all experiments evaluating riboregulator performance using GFP fluorescence, the number of biological replicates was three.

### Cell Viability Assay

The cell viability for the kill switch experiments were evaluated by colony forming unit (CFU) measurements. To construct a kill switch, phage lysis associated genes (S, R, and Rz) were cloned under a toehold switch. Kill switch circuits with or without sRNA were chemically transformed in *E. coli* DH5α, a strain with recA‐deficient genomic background, to reduce recombination events.^[^ ^112^, ^171^ ^]^ All strains were grown in LB agar plate with appropriate antibiotics and 0.2% (w/v) glucose (Cat# G8270‐1KG, Sigma Aldrich, St. Louis, MO, USA) at 37 °C. Cells were then transferred to 1 mL of LB with appropriate antibiotics and 0.2% (w/v) glucose in a 96 deep‐well plate and grown in an orbital shaker at 37 °C and 800 rpm overnight. Overnight cultures were diluted 1:100 in 1 mL of LB with appropriate antibiotics and returned to the orbital shaker. After 80 min, all samples were treated with 1 mм IPTG. Samples were collected after 4 h induction and were washed twice with 1× PBS. Samples were serially diluted in 1× PBS over a 7‐log range. Then, 5 µL of samples were spotted onto LB agar plates with appropriate antibiotics. The plates were incubated at 37 °C overnight before CFU counting. Cell viability (CFU/mL) was calculated by the following formula:

(1) $$\mathrm{CFU}/\mathrm{mL}=\frac{\left(\mathrm{number}\;\mathrm{of}\;\mathrm{colonies}\right)\;\times \;\left(\mathrm{dilution}\;\mathrm{factor}\right)}{0.005\;\mathrm{mL}}$$

To evaluate the stability of a kill switch, permissive groups that did not induce the kill switch were passaged to the next day. Samples were then diluted 1:100 in 1 mL of pre‐warmed LB with appropriate antibiotics and grown in an orbital shaker at 37 °C and 800 rpm. The following day, cell viability assay was conducted based on the overnight culture. The subculturing process was repeated for 30 days. The number of biological replicates for CFU measurements was eight.

### Kill Switch Stability Under Microbial Co‐Culture

To investigate the stability of kill switch under microbial co‐culture, we mixed *E. coli* DH5α containing kill switch circuit and the control strain without the kill switch circuit. Single colonies were grown overnight (∼16 h) in 96‐deep well plates (Cat# 503 102, NEST, Wuxi, Jiangsu, China) with shaking at 800 rpm, 37 °C. On the next day, the kill switch strain without GFP label (GFP‐) and the control strain with GFP label (GFP+) were mixed at ratio of 9:1. The mixtures were then diluted 1:100 in fresh 1 mL LB with appropriate antibiotics. Cells were grown in an orbital shaker at 37 °C and 800 rpm. Sampling was conducted at 6 h intervals, and the samples were immediately fixed for subsequent analysis by flow cytometry. While implementing the microbial co‐culture condition, we passaged the culture into pre‐warmed LB to maintain the exponential phase. At 12 h, the cultures were diluted 1:100 in pre‐warmed fresh 1 mL LB with or without 1 mм IPTG. Sampling continued at 6 h intervals. The ratios of kill switch and control populations at different time points were determined by flow cytometry analysis.

For the 2‐input kill switch experiment, the co‐culture condition was prepared in the same manner where the 2‐input kill switch strain without GFP label (GFP‐) and the control strain with GFP label (GFP+) were mixed at ratio of 9:1. The 1:100 dilution was performed into two separate 96‐deep well plates, designated for 37 and 30 °C conditions, respectively. After dilution, each plate was returned to 37 or 30 °C orbital shaker for continued incubation. Sampling was performed at 12, 20, and 36 h. At 36 h, samples were diluted 1:100 in pre‐warmed 1 mL of LB with or without 1 mм IPTG. The sampling continued at 42, 45, and 48 h. The ratios of 2‐input kill switch and control populations at different time points were determined by flow cytometry analysis.

### Long‐Term Stability Test of the 2‐Input Kill Switch

To examine the long‐term stability and evolutionary robustness of the 2‐input kill switch, the microbial co‐culture condition was maintained under survival conditions (37 °C, without IPTG) for 8 consecutive days. During the experiment, we passaged the cultures into pre‐warmed LB medium with appropriate antibiotics to maintain exponential growth. Sampling was conducted every 12 h, and the population ratios were analyzed by flow cytometry as described above.

To evaluate the functional response over time, samples collected on days 1, 3, 5, and 7 were diluted 1:100 into pre‐warmed 1 mL LB and incubated at 30 °C to assess temperature‐dependent population removal. Likewise, samples collected on days 2, 4, 6, and 8 were diluted 1:100 into pre‐warmed 1 mL LB supplemented with 1 mм IPTG to test response to chemical induction. Population dynamics were monitored by flow cytometry, and the ratios of kill switch and control populations were determined at each time point.

In addition, low‐depth next‐generation sequencing was performed on the day 8 samples using the BTseq sequencing analysis (Bionics, Seoul, Korea) to assess potential genetic changes within the kill switch circuit components. Sequencing analysis that covered the promoter, untranslated region (UTR), and coding sequence (CDS) regions of the circuit, revealed no detectable mutations. All other experimental conditions and analytical procedures followed the microbial co‐culture stability assay described above.

### Statistical Analysis

Statistical analysis and visualization were performed using GraphPad Prism (version 10.6.1). GFP fluorescence was measured by flow cytometry (CytoFLEX S, Beckman Coulter, Brea, CA, USA). The cell population was gated based on FSC and SSC distributions. GFP values were calculated using the geometric mean, and no additional post‐processing was applied to the GFP data. Fold‐change values were calculated as the average across all pairwise combinations of ON and OFF conditions. All experimental results were obtained from at least three biological replicates, with the specific number of replicates indicated in each figure legend. All values are reported as mean ± standard deviation (SD). For Figures S11–S14 (Supporting Information), statistical significance was assessed using Welch's *t*‐tests (**P < 0.01, ****P < 0.0001).

## Conflict of Interest

The authors declare no conflict of interest.

## Supporting information



Supporting Information

## Data Availability

The data that support the findings of this study are available in the supplementary material of this article.

## References

[1] R. L. Rawls , Chem. Eng. News 2000, 78, 49.

[2] W. C. Ruder , T. Lu , J. J. Collins , Science 2011, 333, 1248. 21885773 10.1126/science.1206843

[3] R. Fears , V. ter Meulen , Nat. Rev. Microbiol. 2011, 9, 222. 10.1038/nrmicro2498-c1 21326277

[4] X. Yan , X. Liu , C. Zhao , G. Q. Chen , Signal Transduct. Target. Ther. 2023, 8, 199. 37169742 10.1038/s41392-023-01440-5 PMC10173249

[5] L. Clarke , R. Kitney , Biochem. Soc. Trans. 2020, 48, 113. 32077472 10.1042/BST20190349 PMC7054743

[6] L. Katz , Y. Y. Chen , R. Gonzalez , T. C. Peterson , H. Zhao , R. H. Baltz , J. Ind. Microbiol. Biotechnol. 2018, 45, 449. 29915997 10.1007/s10295-018-2056-y

[7] E. T. Wurtzel , C. E. Vickers , A. D. Hanson , A. H Millar , M. Cooper , K. P. Voss‐Fels , P. I. Nikel , T. J. Erb , Nat. Plants 2019, 5, 1207. 31740769 10.1038/s41477-019-0539-0

[8] M. S. Roell , M. D. Zurbriggen , Curr. Opin. Biotechnol. 2020, 61, 102. 31812911 10.1016/j.copbio.2019.10.004

[9] D. K. Karig , Curr. Opin. Biotechnol. 2017, 45, 69. 28226291 10.1016/j.copbio.2017.01.010

[10] P. R. Yaashikaa , M. K. Devi , P. S. Kumar , Environ. Res. 2022, 214, 113868. 35835162 10.1016/j.envres.2022.113868

[11] J. Aminian‐Dehkordi , S. Rahimi , M. Golzar‐Ahmadi , A. Singh , J. Lopez , R. Ledesma‐Amaro , I. Mijakovic , Biotechnol. Adv. 2023, 68, 108239. 37619824 10.1016/j.biotechadv.2023.108239

[12] E. L. Rylott , N. C. Bruce , Curr. Opin. Chem. Biol. 2020, 58, 86. 32805454 10.1016/j.cbpa.2020.07.004

[13] A. Ahmed , J. V. Rushworth , N. A. Hirst , P. A. Millner , Clin. Microbiol. Rev. 2014, 27, 631. 24982325 10.1128/CMR.00120-13 PMC4135896

[14] D. M. Rawson , A. J. Willmer , A. P. Turner , Biosensors 1989, 4, 299. 2775317 10.1016/0265-928x(89)80011-2

[15] H. P. Austin , M. D. Allen , B. S. Donohoe , et al., Proc. Natl. Acad. Sci. U.S.A. 2018, 115, E4350. 29666242 10.1073/pnas.1718804115 PMC5948967

[16] S. Y. Choi , Y. Lee , H. E. Yu , I. J. Cho , M. Kang , S. Y. Lee , Nat. Microbiol. 2023, 8, 2253. 38030909 10.1038/s41564-023-01529-1

[17] N. Mohanan , Z. Montazer , P. K. Sharma , D. B. Levin , Front. Microbiol. 2020, 11, 580709. 33324366 10.3389/fmicb.2020.580709 PMC7726165

[18] S. J. Yeom , T. K. Le , C. H. Yun , Trends Biotechnol. 2022, 40, 166. 34243985 10.1016/j.tibtech.2021.06.003

[19] J. M. Francois , C. Lachaux , N. Morin , Front. Bioeng. Biotechnol. 2019, 7, 446. 31998710 10.3389/fbioe.2019.00446 PMC6966089

[20] W. Batista‐Silva , P. da Fonseca‐Pereira , A. O. Martins , A. Zsögön , A. Nunes‐Nesi , W. L. Araújo , Plant Commun 2020, 1, 100032. 33367233 10.1016/j.xplc.2020.100032 PMC7747996

[21] C. DeLisi , A. Patrinos , M. MacCracken , D. Drell , G. Annas , A. Arkin , G. Church , R. Cook‐Deegan , H. Jacoby , M. Lidstrom , J. Melillo , R. Milo , K. Paustian , J. Reilly , R. J. Roberts , D. Segrè , S. Solomon , D. Woolf , S. D. Wullschleger , X. Yang , Biodes. Res. 2020, 2020, 1016207. 37849905 10.34133/2020/1016207 PMC10521736

[22] F. Gong , Z. Cai , Y. Li , Sci. China. Life Sci. 2016, 59, 1106. 27787752 10.1007/s11427-016-0304-2

[23] S. Slomovic , K. Pardee , J. J. Collins , Proc. Natl. Acad. Sci. U.S.A. 2015, 112, 14429. 26598662 10.1073/pnas.1508521112 PMC4664311

[24] T. Y. Wei , C. M. Cheng , Cell Chem. Biol. 2016, 23, 1056. 27662252 10.1016/j.chembiol.2016.07.016

[25] X. Tan , J. H. Letendre , J. J. Collins , W. W. Wong , Cell 2021, 184, 881. 33571426 10.1016/j.cell.2021.01.017 PMC7897318

[26] W. Weber , M. Fussenegger , Nat. Rev. Genet. 2011, 13, 21. 22124480 10.1038/nrg3094 PMC7097403

[27] A. Cubillos‐Ruiz , T. Guo , A. Sokolovska , P. F. Miller , J. J. Collins , T. K. Lu , J. M. Lora , Nat. Rev. Drug. Discov. 2021, 20, 941. 34616030 10.1038/s41573-021-00285-3

[28] M. P. McNerney , K. E. Doiron , T. L. Ng , T. Z. Chang , P. A. Silver , Nat. Rev. Genet. 2021, 22, 730. 34234299 10.1038/s41576-021-00383-3 PMC8261392

[29] Z. J. Mays , N. U. Nair , Curr. Opin. Biotechnol. 2018, 53, 224. 29550614 10.1016/j.copbio.2018.01.028 PMC6139064

[30] J. Dou , M. R. Bennett , Biotechnol. J. 2018, 13, 1700159. 10.1002/biot.201700159 PMC5882594 28976641

[31] K. J. Chua , W. C. Kwok , N. Aggarwal , T. Sun , M. W. Chang , Curr. Opin. Chem. Biol. 2017, 40, 8. 28478369 10.1016/j.cbpa.2017.04.011

[32] N. Aggarwal , A. M. E. Breedon , C. M. Davis , I. Y. Hwang , M. W. Chang , Curr. Opin. Biotechnol. 2020, 65, 171. 32304955 10.1016/j.copbio.2020.02.016

[33] J. R. Bober , C. L. Beisel , N. U. Nair , Annu. Rev. Biomed. Eng. 2018, 20, 277. 29528686 10.1146/annurev-bioeng-062117-121019 PMC6100750

[34] D. Endy , Nature 2005, 438, 449. 16306983 10.1038/nature04342

[35] S. A. Benner , A. M. Sismour , Nat. Rev. Genet. 2005, 6, 533. 15995697 10.1038/nrg1637 PMC7097405

[36] N. S. Benabdallah , W. A. Bickmore , Cold Spring Harb. Symp. Quant. Biol. 2015, 80, 45. 26590168 10.1101/sqb.2015.80.027268

[37] T. Ideker , V. Thorsson , J. A. Ranish , R. Christmas , J. Buhler , J. K. Eng , R. Bumgarner , D. R. Goodlett , R. Aebersold , L. Hood , Science 2001, 292, 929. 11340206 10.1126/science.292.5518.929

[38] H. V. Westerhoff , B. O. Palsson , Nat. Biotechnol. 2004, 22, 1249. 15470464 10.1038/nbt1020

[39] H. Jeong , B. Tombor , R. Albert , Z. N. Oltvai , A. L. Barabasi , Nature 2000, 407, 651. 11034217 10.1038/35036627

[40] J. Hasty , D. McMillen , F. Isaacs , J. J. Collins , Nat. Rev. Genet. 2001, 2, 268. 11283699 10.1038/35066056

[41] A. L. Slusarczyk , A. Lin , R. Weiss , Nat. Rev. Genet. 2012, 13, 406. 22596318 10.1038/nrg3227

[42] T. S. Gardner , C. R. Cantor , J. J. Collins , Nature 2000, 403, 339. 10659857 10.1038/35002131

[43] M. B. Elowitz , S. Leibler , Nature 2000, 403, 335. 10659856 10.1038/35002125

[44] M. R. Atkinson , M. A. Savageau , J. T. Myers , A. J. Ninfa , Cell 2003, 113, 597. 12787501 10.1016/s0092-8674(03)00346-5

[45] J. Stricker , S. Cookson , M. R. Bennett , W. H. Mather , L. S. Tsimring , J. Hasty , Nature 2008, 456, 516. 18971928 10.1038/nature07389 PMC6791529

[46] T. Danino , O. Mondragon‐Palomino , L. Tsimring , J. Hasty , Nature 2010, 463, 326. 20090747 10.1038/nature08753 PMC2838179

[47] P. Siuti , J. Yazbek , T. K. Lu , Nat. Biotechnol. 2013, 31, 448. 23396014 10.1038/nbt.2510

[48] J. Bonnet , P. Yin , M. E. Ortiz , P. Subsoontorn , D. Endy , Science 2013, 340, 599. 23539178 10.1126/science.1232758

[49] A. Tamsir , J. J. Tabor , C. A. Voigt , Nature 2011, 469, 212. 21150903 10.1038/nature09565 PMC3904220

[50] T. S. Moon , C. Lou , A. Tamsir , B. C. Stanton , C. A. Voigt , Nature 2012, 491, 249. 23041931 10.1038/nature11516 PMC3904217

[51] M. N. Win , C. D. Smolke , Science 2008, 322, 456. 18927397 10.1126/science.1160311 PMC2805114

[52] J. M. Carothers , J. A. Goler , D. Juminaga , J. D. Keasling , Science 2011, 334, 1716. 22194579 10.1126/science.1212209

[53] B. Wiedenheft , S. H. Sternberg , J. A. Doudna , Nature 2012, 482, 331. 22337052 10.1038/nature10886

[54] L. S. Qi , M. H. Larson , L. A. Gilbert , J. A. Doudna , J. S. Weissman , A. P. Arkin , W. A. Lim , Cell 2013, 152, 1173. 23452860 10.1016/j.cell.2013.02.022 PMC3664290

[55] A. A. K. Nielsen , B. S. Der , J. Shin , P. Vaidyanathan , V. Paralanov , E. A. Strychalski , D. Ross , D. Densmore , C. A. Voigt , Science 2016, 352, aac7341. 27034378 10.1126/science.aac7341

[56] F. J. Isaacs , D. J. Dwyer , C. Ding , D. D. Pervouchine , C. R. Cantor , J. J. Collins , Nat. Biotechnol. 2004, 22, 841. 15208640 10.1038/nbt986

[57] J. Lee , W. Kladwang , M. Lee , D. Cantu , M. Azizyan , H. Kim , A. Limpaecher , S. Gaikwad , S. Yoon , A. Treuille , R. Das , Proc. Natl. Acad. Sci. U.S.A. 2014, 111, 2122. 24469816 10.1073/pnas.1313039111 PMC3926058

[58] G. Rodrigo , T. E. Landrain , A. Jaramillo , Proc. Natl. Acad. Sci. U.S.A. 2012, 109, 15271. 22949707 10.1073/pnas.1203831109 PMC3458397

[59] A. A. Green , P. A. Silver , J. J. Collins , P. Yin , Cell 2014, 159, 925. 25417166 10.1016/j.cell.2014.10.002 PMC4265554

[60] J. M. Callura , D. J. Dwyer , F. J. Isaacs , C. R. Cantor , J. J. Collins , Proc. Natl. Acad. Sci. U.S.A. 2010, 107, 15898. 20713708 10.1073/pnas.1009747107 PMC2936621

[61] Y. Shimoni , G. Friedlander , G. Hetzroni , G. Niv , S. Altuvia , O. Biham , H. Margalit , Mol. Syst. Biol. 2007, 3, 138. 17893699 10.1038/msb4100181 PMC2013925

[62] R. Hussein , H. N. Lim , Nucleic Acids Res. 2012, 40, 7269. 22618873 10.1093/nar/gks439 PMC3424570

[63] P. Mehta , S. Goyal , N. S. Wingreen , Mol. Syst. Biol. 2008, 4, 221. 18854820 10.1038/msb.2008.58 PMC2583084

[64] S. L. Svenningsen , C. M. Waters , B. L. Bassler , Genes Dev. 2008, 22, 226. 18198339 10.1101/gad.1629908 PMC2192756

[65] C. L. Kelly , A. W. K. Harris , H. Steel , E. J. Hancock , J. T. Heap , A. Papachristodoulou , Nucleic Acids Res. 2018, 46, 9875. 30212900 10.1093/nar/gky828 PMC6182179

[66] D. Na , S. M. Yoo , H. Chung , H. Park , J. H. Park , S. Y. Lee , Nat. Biotechnol. 2013, 31, 170. 23334451 10.1038/nbt.2461

[67] J. Mulhbacher , D. A. Lafontaine , Nucleic Acids Res. 2007, 35, 5568. 17704135 10.1093/nar/gkm572 PMC2018637

[68] R. R. Breaker , Mol. Cell 2011, 43, 867. 21925376 10.1016/j.molcel.2011.08.024 PMC4140403

[69] P. J. McCown , K. A. Corbino , S. Stav , M. E. Sherlock , R. R. Breaker , RNA 2017, 23, 995. 28396576 10.1261/rna.061234.117 PMC5473149

[70] E. Nudler , A. S. Mironov , Trends Biochem. Sci. 2004, 29, 11. 14729327 10.1016/j.tibs.2003.11.004

[71] J. Kim , M. Seo , Y. Lim , J. Kim , Adv. Sci. 2024, 11, 2402029. 10.1002/advs.202402029 PMC11423158 39075726

[72] E. A. Doherty , J. A. Doudna , Annu. Rev. Biophys. Biomol. Struct. 2001, 30, 457. 11441810 10.1146/annurev.biophys.30.1.457

[73] A. Wochner , J. Attwater , A. Coulson , P. Holliger , Science 2011, 332, 209. 21474753 10.1126/science.1200752

[74] M. J. Fedor , J. Mol. Biol. 2000, 297, 269. 10715200 10.1006/jmbi.2000.3560

[75] C. Lou , B. Stanton , Y. J. Chen , B. Munsky , C. A. Voigt , Nat. Biotechnol. 2012, 30, 1137. 23034349 10.1038/nbt.2401 PMC3914141

[76] Z. Xie , L. Wroblewska , L. Prochazka , R. Weiss , Y. Benenson , Science 2011, 333, 1307. 21885784 10.1126/science.1205527

[77] T. Quarton , K. Ehrhardt , J. Lee , S. Kannan , Y. Li , L. Ma , L. Bleris , npj Syst. Biol. Appl. 2018, 4, 6. 29354284 10.1038/s41540-017-0043-y PMC5765153

[78] S. Matsuura , H. Ono , S. Kawasaki , Y. Kuang , Y. Fujita , H. Saito , Nat. Commun. 2018, 9, 4847. 30451868 10.1038/s41467-018-07181-2 PMC6242901

[79] J. S. Cho , D. Yang , C. P. S. Prabowo , M. R. Ghiffary , T. Han , K. R. Choi , C. W. Moon , H. Zhou , J. Y. Ryu , H. U. Kim , S. Y. Lee , Nat. Commun. 2023, 14, 2359. 37095132 10.1038/s41467-023-38119-y PMC10126203

[80] S. M. Yoo , D. Na , S. Y. Lee , Nat. Protoc. 2013, 8, 1694. 23928502 10.1038/nprot.2013.105

[81] M. Noh , S. M. Yoo , W. J. Kim , S. Y. Lee , Cell Syst 2017, 5, 418. 28964700 10.1016/j.cels.2017.08.016

[82] J. Ren , N. T. Nong , P. N. Lam Vo , H. M. Lee , D. Na , ACS Synth. Biol. 2024, 13, 3256. 39294875 10.1021/acssynbio.4c00323

[83] A. Ghodasara , C. A. Voigt , Nucleic Acids Res. 2017, 45, 8116. 28609783 10.1093/nar/gkx530 PMC5737548

[84] J. Lin , Y. Liu , P. Lai , H. Ye , L. Xu , Nucleic Acids Res. 2020, 48, 11773. 33068434 10.1093/nar/gkaa842 PMC7672423

[85] M. H. Hanewich‐Hollatz , Z. Chen , L. M. Hochrein , J. Huang , N. A. Pierce , ACS Cent. Sci. 2019, 5, 1241. 31403072 10.1021/acscentsci.9b00340 PMC6661866

[86] H. Kang , D. Park , J. Kim , Nucleic Acids Res. 2024, 52, 8595. 38943344 10.1093/nar/gkae549 PMC11317168

[87] M. Patchsung , K. Jantarug , A. Pattama , K. Aphicho , S. Suraritdechachai , P. Meesawat , K. Sappakhaw , N. Leelahakorn , T. Ruenkam , T. Wongsatit , N. Athipanyasilp , B. Eiamthong , B. Lakkanasirorat , T. Phoodokmai , N. Niljianskul , D. Pakotiprapha , S. Chanarat , A. Homchan , R. Tinikul , P. Kamutira , K. Phiwkaow , S. Soithongcharoen , C. Kantiwiriyawanitch , V. Pongsupasa , D. Trisrivirat , J. Jaroensuk , T. Wongnate , S. Maenpuen , P. Chaiyen , S. Kamnerdnakta , et al., Nat. Biomed. Eng. 2020, 4, 1140. 32848209 10.1038/s41551-020-00603-x

[88] M. J. Kellner , J. G. Koob , J. S. Gootenberg , O. O. Abudayyeh , F. Zhang , Nat. Protoc. 2019, 14, 2986. 31548639 10.1038/s41596-019-0210-2 PMC6956564

[89] K. Pardee , A. A. Green , M. K. Takahashi , D. Braff , G. Lambert , J. W. Lee , T. Ferrante , D. Ma , N. Donghia , M. Fan , N. M. Daringer , I. Bosch , D. M. Dudley , D. H. O'Connor , L. Gehrke , J. J. Collins , Cell 2016, 165, 1255. 27160350 10.1016/j.cell.2016.04.059

[90] S. Park , J. W. Lee , Int. J. Mol. Sci. 2021, 22.

[91] F. Hong , D. Ma , K. Wu , L. A. Mina , R. C. Luiten , Y. Liu , H. Yan , A. A. Green , Cell 2020, 180, 1018. 32109416 10.1016/j.cell.2020.02.011 PMC7063572

[92] W. Choi , E. Park , S. Bae , K.‐H. Choi , S. Han , K.‐H. Son , D. Y. Lee , I.‐J. Cho , H. Seong , K. S. Hwang , J.‐M. Nam , J. Choi , H. Lee , N. Choi , Small 2022, 18, 2105538. 10.1002/smll.202105538 34923738

[93] Z. F. Gao , Y. Ling , L. Lu , N. Y. Chen , H. Q. Luo , N. B. Li , Anal. Chem. 2014, 86, 2543. 24527790 10.1021/ac500362z

[94] T. M. Henkin , Genes Dev. 2008, 22, 3383. 19141470 10.1101/gad.1747308 PMC3959987

[95] M. You , J. L. Litke , S. R. Jaffrey , Proc. Natl. Acad. Sci. U.S.A. 112, 2015, E2756. 25964329 10.1073/pnas.1504354112 PMC4450428

[96] S. Jang , S. Jang , Y. Xiu , T. J. Kang , S.‐H. Lee , M. A. G. Koffas , G. Y. Jung , ACS Synth. Biol. 2017, 6, 2077. 28749656 10.1021/acssynbio.7b00128

[97] P. Noll , C. Treinen , S. Müller , L. Lilge , R. Hausmann , M. Henkel , Sci. Rep. 2021, 11, 14802. 34285304 10.1038/s41598-021-94400-4 PMC8292423

[98] J. A. Brophy , C. A. Voigt , Nat. Methods 2014, 11, 508. 24781324 10.1038/nmeth.2926 PMC4230274

[99] T. S. Jones , S. M. D. Oliveira , C. J. Myers , C. A. Voigt , D. Densmore , Nat. Protoc. 2022, 17, 1097. 35197606 10.1038/s41596-021-00675-2

[100] J. Kim , Y. Zhou , P. D. Carlson , M. Teichmann , S. Chaudhary , F. C. Simmel , P. A. Silver , J. J. Collins , J. B. Lucks , P. Yin , A. A. Green , Nat. Chem. Biol. 2019, 15, 1173. 31686032 10.1038/s41589-019-0388-1 PMC6864284

[101] A. A. Green , J. Kim , D. Ma , P. A. Silver , J. J. Collins , P. Yin , Nature 2017, 548, 117. 28746304 10.1038/nature23271 PMC6078203

[102] P. E. Purnick , R. Weiss , Nat. Rev. Mol. Cell Biol. 2009, 10, 410. 19461664 10.1038/nrm2698

[103] R. S. Cox 3rd , M. G. Surette , M. B. Elowitz , Mol. Syst. Biol. 2007, 3, 145. 18004278 10.1038/msb4100187 PMC2132448

[104] Y. Chen , S. Zhang , E. M. Young , T. S. Jones , D. Densmore , C. A. Voigt , Nat. Microbiol. 2020, 5, 1349. 32747797 10.1038/s41564-020-0757-2

[105] D. A. Oyarzun , G. B. Stan , J. R. Soc. Interface 2013, 10, 20120671. 23054953 10.1098/rsif.2012.0671 PMC3565798

[106] G. De Carluccio , V. Fusco , D. di Bernardo , Nat. Commun. 2024, 15, 3311. 38632224 10.1038/s41467-024-47592-y PMC11024104

[107] J. L. Chlebek , S. P. Leonard , C. Kang‐Yun , M. C. Yung , D. P. Ricci , Y. Jiao , D. M. Park , Nucleic Acids Res. 2023, 51, 7094. 37260076 10.1093/nar/gkad484 PMC10359631

[108] S. R. Lindemann , H. C. Bernstein , H.‐S. Song , J. K. Fredrickson , M. W. Fields , W. Shou , D. R. Johnson , A. S. Beliaev , ISME J 2016, 10, 2077. 26967105 10.1038/ismej.2016.26 PMC4989317

[109] A. A. Nielsen , C. A. Voigt , Mol. Syst. Biol. 2014, 10, 763. 25422271 10.15252/msb.20145735 PMC4299604

[110] M. E. Lee , W. C. DeLoache , B. Cervantes , J. E. Dueber , ACS Synth. Biol. 2015, 4, 975. 25871405 10.1021/sb500366v

[111] H. M. Salis , E. A. Mirsky , C. A. Voigt , Nat. Biotechnol. 2009, 27, 946. 19801975 10.1038/nbt.1568 PMC2782888

[112] C. T. Chan , J. W. Lee , D. E. Cameron , C. J. Bashor , J. J. Collins , Nat. Chem. Biol. 2016, 12, 82. 26641934 10.1038/nchembio.1979 PMC4718764

[113] F. Stirling , L. Bitzan , S. O'Keefe , E. Redfield , J. W. K. Oliver , J. Way , P. A. Silver , Mol. Cell 2017, 68, 686. 29149596 10.1016/j.molcel.2017.10.033 PMC5812007

[114] A. G. Rottinghaus , A. Ferreiro , S. R. S. Fishbein , G. Dantas , T. S. Moon , Nat. Commun. 2022, 13, 672. 35115506 10.1038/s41467-022-28163-5 PMC8813983

[115] R. Chao , S. Mishra , T. Si , H. Zhao , Metab. Eng. 2017, 42, 98. 28602523 10.1016/j.ymben.2017.06.003 PMC5544601

[116] D. T. C. Chan , L. Winter , J. Bjerg , S. Krsmanovic , G. S. Baldwin , H. C. Bernstein , ACS Synth. Biol. 2025, 14, 193. 39754601 10.1021/acssynbio.4c00551 PMC11744933

[117] P. Carbonell , A. J. Jervis , C. J. Robinson , C. Yan , M. Dunstan , N. Swainston , M. Vinaixa , K. A. Hollywood , A. Currin , N. J. W. Rattray , S. Taylor , R. Spiess , R. Sung , A. R. Williams , D. Fellows , N. J. Stanford , P. Mulherin , R. Le Feuvre , P. Barran , R. Goodacre , N. J. Turner , C. Goble , G. G. Chen , D. B. Kell , J. Micklefield , R. Breitling , E. Takano , J.‐L. Faulon , N. S. Scrutton , Commun. Biol. 2018, 1, 66. 30271948 10.1038/s42003-018-0076-9 PMC6123781

[118] I. Mariam , U. Rova , P. Christakopoulos , L. Matsakas , A. Patel , npj Syst. Biol. Appl. 2025, 11, 74. 40624030 10.1038/s41540-025-00556-4 PMC12234913

[119] P. van Lent , J. Schmitz , T. Abeel , ACS Synth. Biol. 2023, 12, 2588. 37616156 10.1021/acssynbio.3c00186 PMC10510747

[120] N. Gurdo , D. C. Volke , P. I. Nikel , Trends Biotechnol. 2022, 40, 1148. 35410817 10.1016/j.tibtech.2022.03.004

[121] A.‐C. Groher , S. Jager , C. Schneider , F. Groher , K. Hamacher , B. Suess , ACS Synth. Biol. 2019, 8, 34. 30513199 10.1021/acssynbio.8b00207

[122] N. M. Angenent‐Mari , A. S. Garruss , L. R. Soenksen , G. Church , J. J. Collins , Nat. Commun. 2020, 11, 5057. 33028812 10.1038/s41467-020-18677-1 PMC7541447

[123] J. A. Valeri , K. M. Collins , P. Ramesh , M. A. Alcantar , B. A. Lepe , T. K. Lu , D. M. Camacho , Nat. Commun. 2020, 11, 5058. 33028819 10.1038/s41467-020-18676-2 PMC7541510

[124] M. Wachsmuth , S. Findeiss , N. Weissheimer , P. F. Stadler , M. Morl , Nucleic Acids Res. 2013, 41, 2541. 23275562 10.1093/nar/gks1330 PMC3575828

[125] Y. H. Lin , K. Y. Chang , Nucleic Acids Res. 2016, 44, 9005. 27521370 10.1093/nar/gkw718 PMC5062990

[126] A. Ogawa , RNA 2011, 17, 478. 21224378 10.1261/rna.2433111 PMC3039147

[127] S. A. Lynch , S. K. Desai , H. K. Sajja , J. P. Gallivan , Chem. Biol. 2007, 14, 173. 17317571 10.1016/j.chembiol.2006.12.008 PMC1858662

[128] S. A. Lynch , S. Topp , J. P. Gallivan , Methods Mol. Biol. 2009, 540, 321. 19381570 10.1007/978-1-59745-558-9_23

[129] T. Tabuchi , Y. Yokobayashi , Nucleic Acids Res. 2022, 50, 3535. 35253887 10.1093/nar/gkac152 PMC8989549

[130] B. Strobel , M. Spöring , H. Klein , D. Blazevic , W. Rust , S. Sayols , J. S. Hartig , S. Kreuz , Nat. Commun. 2020, 11, 714. 32024835 10.1038/s41467-020-14491-x PMC7002664

[131] Y. Wang , P. Xue , M. Cao , T. Yu , S. T. Lane , H. Zhao , Chem. Rev. 2021, 121, 12384. 34297541 10.1021/acs.chemrev.1c00260

[132] M. J. Dougherty , F. H. Arnold , Curr. Opin. Biotechnol. 2009, 20, 486. 19720520 10.1016/j.copbio.2009.08.005 PMC2775421

[133] Y. Yokobayashi , R. Weiss , F. H. Arnold , Proc. Natl. Acad. Sci. U.S.A. 2002, 99, 16587. 12451174 10.1073/pnas.252535999 PMC139187

[134] E. L. Haseltine , F. H. Arnold , Annu. Rev. Biophys. Biomol. Struct. 2007, 36, 1. 17243895 10.1146/annurev.biophys.36.040306.132600

[135] M. K. Goshisht , ACS Omega 2024, 9, 9921. 38463314 10.1021/acsomega.3c05913 PMC10918679

[136] M. Zhang , M. B. Holowko , H. Hayman Zumpe , C. S. Ong , ACS Synth. Biol. 2022, 11, 2314. 35704784 10.1021/acssynbio.2c00015 PMC9295160

[137] F. Bubeck , M. D. Hoffmann , Z. Harteveld , S. Aschenbrenner , A. Bietz , M. C. Waldhauer , K. Börner , J. Fakhiri , C. Schmelas , L. Dietz , D. Grimm , B. E. Correia , R. Eils , D. Niopek , Nat. Methods 2018, 15, 924. 30377362 10.1038/s41592-018-0178-9

[138] M. Nakamura , P. Srinivasan , M. Chavez , M. A. Carter , A. A. Dominguez , M. La Russa , M. B. Lau , T. R. Abbott , X. Xu , D. Zhao , Y. Gao , N. H. Kipniss , C. D. Smolke , J. Bondy‐Denomy , L. S. Qi , Nat. Commun. 2019, 10, 194. 30643127 10.1038/s41467-018-08158-x PMC6331597

[139] X. Wan , F. Pinto , L. Yu , B. Wang , Nat. Commun. 2020, 11, 5961. 33235249 10.1038/s41467-020-19552-9 PMC7686491

[140] A. Lavenniah , T. D. A. Luu , Y. P. Li , T. B. Lim , J. Jiang , M. Ackers‐Johnson , R. S.‐Y. Foo , Mol. Ther. 2020, 28, 1506. 32304667 10.1016/j.ymthe.2020.04.006 PMC7264434

[141] S. Gottesman , G. Storz , Cold Spring Harb. Perspect. Biol. 2011, 3, a003798. 20980440 10.1101/cshperspect.a003798 PMC3225950

[142] E. G. H. Wagner , P. Romby , Adv. Genet. 2015, 90, 133. 26296935 10.1016/bs.adgen.2015.05.001

[143] G. Storz , J. Vogel , K. M. Wassarman , Mol. Cell 2011, 43, 880. 21925377 10.1016/j.molcel.2011.08.022 PMC3176440

[144] J. Vogel , B. F. Luisi , Nat. Rev. Microbiol. 2011, 9, 578. 21760622 10.1038/nrmicro2615 PMC4615618

[145] J. Chappell , M. K. Takahashi , J. B. Lucks , Nat. Chem. Biol. 2015, 11, 214. 25643173 10.1038/nchembio.1737

[146] Y. Nakahira , A. Ogawa , H. Asano , T. Oyama , Y. Tozawa , Plant Cell Physiol. 2013, 54, 1724. 23969558 10.1093/pcp/pct115

[147] J. Rinnenthal , B. Klinkert , F. Narberhaus , H. Schwalbe , Nucleic Acids Res. 2011, 39, 8258. 21727085 10.1093/nar/gkr314 PMC3185406

[148] D. I. Piraner , M. H. Abedi , B. A. Moser , A. Lee‐Gosselin , M. G. Shapiro , Nat. Chem. Biol. 2017, 13, 75. 27842069 10.1038/nchembio.2233

[149] S. GOTTESMAN , C. A. McCULLEN , M. GUILLIER , C. K. VANDERPOOL , N. MAJDALANI , J. BENHAMMOU , K. M. THOMPSON , P. C. FitzGERALD , N. A. SOWA , D. J. FitzGERALD , Cold Spring Harb. Symp. Quant. Biol. 2006, 71, 1. 17381274 10.1101/sqb.2006.71.016 PMC3592358

[150] N. Majdalani , C. K. Vanderpool , S. Gottesman , Crit. Rev. Biochem. Mol. Biol. 2005, 40, 93. 15814430 10.1080/10409230590918702

[151] A. Lavi‐Itzkovitz , N. Peterman , D. Jost , E. Levine , Nucleic Acids Res. 2014, 42, 12200. 25294829 10.1093/nar/gku889 PMC4231754

[152] B. G. Sahagan , J. E. Dahlberg , J. Mol. Biol. 1979, 131, 593. 229230 10.1016/0022-2836(79)90009-3

[153] B. R. Wolfe , N. J. Porubsky , J. N. Zadeh , R. M. Dirks , N. A. Pierce , J. Am. Chem. Soc. 2017, 139, 3134. 28191938 10.1021/jacs.6b12693

[154] M. E. Fornace , N. J. Porubsky , N. A. Pierce , ACS Synth. Biol. 2020, 9, 2665. 32910644 10.1021/acssynbio.9b00523

[155] M. K. Takahashi , X. Tan , A. J. Dy , D. Braff , R. T. Akana , Y. Furuta , N. Donghia , A. Ananthakrishnan , J. J. Collins , Nat. Commun. 2018, 9, 3347. 30131493 10.1038/s41467-018-05864-4 PMC6104080

[156] T. Wang , F. C. Simmel , Nucleic Acids Res. 2022, 50, 4784. 35446427 10.1093/nar/gkac275 PMC9071393

[157] D. Ma , Y. Li , K. Wu , Z. Yan , A. A. Tang , S. Chaudhary , Z. M. Ticktin , J. Alcantar‐Fernandez , J. L. Moreno‐Camacho , A. Campos‐Romero , A. A. Green , Nat. Biomed. Eng. 2022, 6, 298. 35288660 10.1038/s41551-022-00857-7 PMC8940621

[158] S. J. Kim , M. Leong , M. B. Amrofell , Y. J. Lee , T. S. Moon , ACS Synth. Biol. 2019, 8, 601. 30721039 10.1021/acssynbio.8b00488

[159] Y. J. Lee , S. J. Kim , M. B. Amrofell , T. S. Moon , ACS Synth. Biol. 2019, 8, 45. 30517781 10.1021/acssynbio.8b00227

[160] J. Chappell , A. Westbrook , M. Verosloff , J. B. Lucks , Nat. Commun. 2017, 8, 1051. 29051490 10.1038/s41467-017-01082-6 PMC5648800

[161] S. Hooshangi , S. Thiberge , R. Weiss , Proc. Natl. Acad. Sci. USA 2005, 102, 3581. 15738412 10.1073/pnas.0408507102 PMC552778

[162] K. F. Murphy , R. M. Adams , X. Wang , G. Balazsi , J. J. Collins , Nucleic Acids Res. 2010, 38, 2712. 20211838 10.1093/nar/gkq091 PMC2860118

[163] T. Ozdemir , A. J. H. Fedorec , T. Danino , C. P. Barnes , Cell Syst 2018, 7, 5. 30048620 10.1016/j.cels.2018.06.008

[164] M. L. Pardi , J. Wu , S. Kawasaki , H. Saito , Adv. Drug Delivery Rev. 2022, 184, 114196. 10.1016/j.addr.2022.114196 35288218

[165] F. Sedlmayer , D. Aubel , M. Fussenegger , Nat. Biomed. Eng. 2018, 2, 399. 31011195 10.1038/s41551-018-0215-0

[166] J. W. Lee , C. T. Y. Chan , S. Slomovic , J. J. Collins , Nat. Chem. Biol. 2018, 14, 530. 29769737 10.1038/s41589-018-0056-x

[167] A. Contreras , S. Molin , J. L. Ramos , Appl. Environ. Microbiol. 1991, 57, 1504. 16348490 10.1128/aem.57.5.1504-1508.1991 PMC182976

[168] L. B. Jensen , J. L. Ramos , Z. Kaneva , S. Molin , Appl. Environ. Microbiol. 1993, 59, 3713. 8285679 10.1128/aem.59.11.3713-3717.1993 PMC182522

[169] H. Goh , S. Choi , J. Kim , Nucleic Acids Res. 2024, 52, 13469. 39526390 10.1093/nar/gkae980 PMC11602170

[170] R. Young , U. Blasi , FEMS Microbiol. Rev. 1995, 17, 191. 7669346 10.1111/j.1574-6976.1995.tb00202.x

[171] T. D. Thi , E. Lopez , A. Rodriguez‐Rojas , J. Rodriguez‐Beltran , A. Couce , J. R. Guelfo , A. Castaneda‐Garcia , J. Blazquez , J. Antimicrob. Chemother. 2011, 66, 531. 21212055 10.1093/jac/dkq496

[172] V. M. Isabella , B. N. Ha , M. J. Castillo , D. J. Lubkowicz , S. E. Rowe , Y. A. Millet , C. L. Anderson , N. Li , A. B. Fisher , K. A. West , P. J. Reeder , M. M. Momin , C. G. Bergeron , S. E. Guilmain , P. F. Miller , C. B. Kurtz , D. Falb , Nat. Biotechnol. 2018, 36, 857. 30102294 10.1038/nbt.4222

[173] T. S. Moon , Methods Mol. Biol. 2022, 2518, 111. 35666442 10.1007/978-1-0716-2421-0_7

[174] A. K. Velazquez Sanchez , B. Klopprogge , K. H. Zimmermann , Z. Ignatova , ACS Synth. Biol. 2023, 12, 2524. 37595156 10.1021/acssynbio.2c00614

[175] J. Vazquez‐Anderson , L. M. Contreras , RNA Biol 2013, 10, 1778. 24356572 10.4161/rna.27102 PMC3917981

[176] D. K. Agrawal , X. Tang , A. Westbrook , R. Marshall , C. S. Maxwell , J. Lucks , V. Noireaux , C. L. Beisel , M. J. Dunlop , E. Franco , ACS Synth. Biol. 2018, 7, 1219. 29709170 10.1021/acssynbio.8b00040

[177] G. R. Posfai , G. Plunkett , T. S. Feher , D. Frisch , G. N. M. Keil , K. Umenhoffer , V. Kolisnychenko , B. Stahl , S. S. Sharma , M. de Arruda , V. Burland , S. W. Harcum , F. R. Blattner , Science 2006, 312, 1044. 16645050 10.1126/science.1126439

[178] F. Ceroni , R. Algar , G. B. Stan , T. Ellis , Nat. Methods 2015, 12, 415. 25849635 10.1038/nmeth.3339

[179] J. Chappell , K. E. Watters , M. K. Takahashi , J. B. Lucks , Curr. Opin. Chem. Biol. 2015, 28, 47. 26093826 10.1016/j.cbpa.2015.05.018

[180] J. Chappell , M. K. Takahashi , S. Meyer , D. Loughrey , K. E. Watters , J. Lucks , Biotechnol. J. 2013, 8, 1379. 24124015 10.1002/biot.201300018 PMC4033574

[181] C. A. Hutchison , R.‐Y. Chuang , V. N. Noskov , N. Assad‐Garcia , T. J. Deerinck , M. H. Ellisman , J. Gill , K. Kannan , B. J. Karas , L. Ma , J. F. Pelletier , Z.‐Q. Qi , R. A Richter , E. A. Strychalski , L. Sun , Y. Suzuki , B. Tsvetanova , K. S. Wise , H. O. Smith , J. I. Glass , C. Merryman , D. G. Gibson , J. C Venter , Science 2016, 351, aad6253. 27013737 10.1126/science.aad6253

[182] R. Z. Moger‐Reischer , J. I. Glass , K. S. Wise , L. Sun , D. M. C. Bittencourt , B. K. Lehmkuhl , D. R. Schoolmaster , M. Lynch , J. T. Lennon , Nature 2023, 620, 122. 37407813 10.1038/s41586-023-06288-x PMC10396959

[183] P. Litovco , N. Barger , X. Li , R. Daniel , Nucleic Acids Res. 2021, 49, 5393. 34009384 10.1093/nar/gkab253 PMC8136830

[184] R. Zhang , J. Li , J. Melendez‐Alvarez , X. Chen , P. Sochor , H. Goetz , Q. Zhang , T. Ding , X. Wang , X.‐J. Tian , Nat. Chem. Biol. 2020, 16, 695. 32251409 10.1038/s41589-020-0509-x PMC7246135

[185] T. Y.‐C. Tsai , Y. S. Choi , W. Ma , J. R. Pomerening , C. Tang , J. E. Ferrell , Science 2008, 321, 126. 18599789 10.1126/science.1156951 PMC2728800

[186] M. T. Guinn , G. Balazsi , Nucleic Acids Res. 2019, 47, 7703. 31269201 10.1093/nar/gkz556 PMC6698750

[187] Z. Sun , W. Wei , M. Zhang , W. Shi , Y. Zong , Y. Chen , X. Yang , B. Yu , C. Tang , C. Lou , Nucleic Acids Res. 2022, 50, 2377. 35166832 10.1093/nar/gkac066 PMC8887471

[188] X. J. Tian , X. P. Zhang , F. Liu , W. Wang , Phys. Rev. E Stat. Nonlin. Soft Matter Phys. 2009, 80, 011926. 19658748 10.1103/PhysRevE.80.011926

[189] R. Kent , N. Dixon , ACS Synth. Biol. 2019, 8, 884. 30897329 10.1021/acssynbio.9b00017 PMC6492952

[190] J. Ang , E. Harris , B. J. Hussey , R. Kil , D. R. McMillen , ACS Synth. Biol. 2013, 2, 547. 23905721 10.1021/sb4000564 PMC3805330

[191] A. Hoynes‐O'Connor , T. S. Moon , ACS Synth. Biol. 2016, 5, 1441. 27434774 10.1021/acssynbio.6b00036

[192] A. Busch , A. S. Richter , R. Backofen , Bioinformatics 2008, 24, 2849. 18940824 10.1093/bioinformatics/btn544 PMC2639303

[193] M. Mann , P. R. Wright , R. Backofen , Nucleic Acids Res. 2017, 45, W435. 28472523 10.1093/nar/gkx279 PMC5570192

[194] C. Smith , S. Heyne , A. S. Richter , S. Will , R. Backofen , Nucleic Acids Res. 2010, 38, W373. 20444875 10.1093/nar/gkq316 PMC2896085

[195] S. U. Umu , P. P. Gardner , Bioinformatics 2017, 33, 988. 27993777 10.1093/bioinformatics/btw728 PMC5408919

[196] P. R. Wright , J. Georg , M. Mann , D. A. Sorescu , A. S. Richter , S. Lott , R. Kleinkauf , W. R. Hess , R. Backofen , Nucleic Acids Res. 2014, 42, W119. 24838564 10.1093/nar/gku359 PMC4086077

[197] S. L. Ameres , J. Martinez , R. Schroeder , Cell 2007, 130, 101. 17632058 10.1016/j.cell.2007.04.037

[198] M. Kertesz , N. Iovino , U. Unnerstall , U. Gaul , E. Segal , Nat. Genet. 2007, 39, 1278. 17893677 10.1038/ng2135

[199] K. Prevost , G. Desnoyers , J. F. Jacques , F. Lavoie , E. Masse , Genes Dev. 2011, 25, 385. 21289064 10.1101/gad.2001711 PMC3042161

[200] N. De Lay , D. J. Schu , S. Gottesman , J. Biol. Chem. 2013, 288, 7996. 23362267 10.1074/jbc.R112.441386 PMC3605619

[201] B. K. Mohanty , S. R. Kushner , Mol. Microbiol. 2003, 50, 645. 14617186 10.1046/j.1365-2958.2003.03724.x

[202] F. Ponath , J. Hor , J. Vogel , FEMS Microbiol. Rev. 2022, 46, fuac017. 35388892 10.1093/femsre/fuac017 PMC9438474

[203] D. Lalaouna , M. Simoneau‐Roy , D. Lafontaine , E. Masse , Biochim. Biophys. Acta 2013, 1829, 742. 23500183 10.1016/j.bbagrm.2013.02.013

[204] J. Yeom , J. S. Park , Y. M. Jeon , B. S. Song , S. M. Yoo , Appl. Microbiol. Biotechnol. 2022, 106, 2517. 35291022 10.1007/s00253-022-11867-5

[205] D. Yang , S. M. Yoo , C. Gu , J. Y. Ryu , J. E. Lee , S. Y. Lee , Metab. Eng. 2019, 54, 180. 30999052 10.1016/j.ymben.2019.04.003

[206] A. Lahiry , S. D. Stimple , D. W. Wood , R. A. Lease , ACS Synth. Biol. 2017, 6, 648. 28067500 10.1021/acssynbio.6b00261

[207] K. Papenfort , E. Espinosa , J. Casadesus , J. Vogel , Proc. Natl. Acad. Sci. U.S.A. 2015, 112, E4772. 26307765 10.1073/pnas.1507825112 PMC4553797

[208] W. H. Xie , H. K. Deng , J. Hou , L. J. Wang , Appl. Microbiol. Biotechnol. 2021, 105, 1. 33201273 10.1007/s00253-020-10971-8

[209] P. Apura , M. Saramago , A. Peregrina , S. C. Viegas , S. M. Carvalho , L. M. Saraiva , C. M. Arraiano , S. Domingues , Plasmid 2020, 109, 102503. 32209400 10.1016/j.plasmid.2020.102503

[210] D. Sun , J. Chen , Y. Wang , M. Li , D. Rao , Y. Guo , N. Chen , P. Zheng , J. Sun , Y. Ma , J. Ind. Microbiol. Biotechnol. 2019, 46, 203. 30666532 10.1007/s10295-018-02128-4

[211] Z. Lin , J. Li , X. Yan , J. Yang , X. Li , P. Chen , X. Yang , Appl. Environ. Microbiol. 2021, 87. 10.1128/AEM.02923-20 PMC8117753 33674434

[212] T. B. Updegrove , A. Zhang , G. Storz , Curr. Opin. Microbiol. 2016, 30, 133. 26907610 10.1016/j.mib.2016.02.003 PMC4821791

[213] K. Ziolkowska , Nucleic Acids Res. 2006, 34, 709. 16449205 10.1093/nar/gkj464 PMC1356530

[214] J. Caillet , C. Gracia , F. Fontaine , E. Hajnsdorf , RNA 2014, 20, 1567. 25147238 10.1261/rna.043372.113 PMC4174439

[215] S. Melamed , A. Peer , R. Faigenbaum‐Romm , Y. E. Gatt , N. Reiss , A. Bar , Y. Altuvia , L. Argaman , H. Margalit , Mol. Cell 2016, 63, 884. 27588604 10.1016/j.molcel.2016.07.026 PMC5145812

[216] S. W. McKellar , I. Ivanova , P. Arede , R. L. Zapf , N. Mercier , L.‐C. Chu , D. G. Mediati , A. C. Pickering , P. Briaud , R. G. Foster , G. Kudla , J. R Fitzgerald , I. Caldelari , R. K. Carroll , J. J. Tree , S. Granneman , Nat. Commun. 2022, 13, 3560. 35732654 10.1038/s41467-022-31173-y PMC9217828

[217] M. Kushwaha , H. M. Salis , Nat. Commun. 2015, 6, 7832. 26184393 10.1038/ncomms8832 PMC4518296

[218] A. Stone , A. Youssef , S. Rijal , R. Zhang , X. J. Tian , Trends Biotechnol. 2024, 42, 895. 38320912 10.1016/j.tibtech.2024.01.003 PMC11223972

[219] C. Helenek , R. Krzyszton , J. Petreczky , Y. Wan , M. Cabral , D. Coraci , G. Balázsi , Cell Chem. Biol. 2024, 31, 1447. 38925113 10.1016/j.chembiol.2024.05.018 PMC11330362

[220] E. Andrianantoandro , S. Basu , D. K. Karig , R. Weiss , Mol. Syst. Biol. 2006, 2, MSB4100073. 10.1038/msb4100073 PMC1681505 16738572

[221] S. Cardinale , A. P. Arkin , Biotechnol. J. 2012, 7, 856. 22649052 10.1002/biot.201200085 PMC3440575

[222] R. Grutzner , S. Marillonnet , Methods Mol. Biol. 2020, 2205, 107. 32809196 10.1007/978-1-0716-0908-8_7

[223] E. Weber , C. Engler , R. Gruetzner , S. Werner , S. Marillonnet , PLoS One 2011, 6, 16765. 10.1371/journal.pone.0019722 PMC3098256 21625552

[224] S. J. Moore , H.‐E. Lai , R. J. R. Kelwick , S. M. Chee , D. J. Bell , K. M. Polizzi , P. S. Freemont , ACS Synth. Biol. 2016, 5, 1059. 27096716 10.1021/acssynbio.6b00031

[225] E. Martínez‐García , S. Fraile , E. Algar , T. Aparicio , E. Velázquez , B. Calles , H. Tas , B. Blázquez , B. Martín , C. Prieto , L. Sánchez‐Sampedro , M. H. H. Nørholm , D. C. Volke , N. T. Wirth , P. Dvořák , L. Alejaldre , L. Grozinger , M. Crowther , A. Goñi‐Moreno , P. I. Nikel , J. Nogales , V. d. Lorenzo , Nucleic Acids Res. 2023, 51, D1558. 36420904 10.1093/nar/gkac1059 PMC9825617

[226] A. Dwijayanti , M. Storch , G. B. Stan , G. S. Baldwin , Nucleic Acids Res. 2022, 50, 1783. 35061908 10.1093/nar/gkab1301 PMC8860615

[227] Y. Liu , Y. Zhu , J. Li , H.‐D. Shin , R. R. Chen , G. Du , L. Liu , J. Chen , Metab. Eng. 2014, 23, 42. 24560814 10.1016/j.ymben.2014.02.005

[228] C. Cho , S. Y. Lee , Biotechnol. Bioeng. 2017, 114, 374. 27531464 10.1002/bit.26077

[229] J. Quan , J. Tian , Nat. Protoc. 2011, 6, 242. 21293463 10.1038/nprot.2010.181

[230] H. Liu , J. H. Naismith , BMC Biotechnol. 2008, 8, 91. 19055817 10.1186/1472-6750-8-91 PMC2629768

