# Supplementary material for: SUPER: Upcycling Genetic Parts for Precise Gene Expression Control, Leakage Minimization, and Genetic Circuit Stability
Source: Adv Sci (Weinh). 2025 Dec 15;13(12):e14653. doi: 10.1002/advs.202514653 (PMC12948289; doi:10.1002/advs.202514653)
Supplement: Supplementary file 1 — Supporting Information [file ADVS-13-e14653-s001.pdf]

WILEY-VCH

Supporting Information for

# SUPER: Upcycling Genetic Parts for Precise Gene Expression Control, Leakage Minimization, and Genetic Circuit Stability

Teayang Heo†, Dongwon Park†, Woosub Shin and Jongmin Kim*

Department of Life Sciences, Pohang University of Science and Technology, Pohang 37673, Korea

* To whom correspondence should be addressed. Tel: +82-54-279-2322; Fax: 82-54-279-0666 Email: jongmin.kim@postech.ac.kr

T.H. and D.P. contributed equally, and both have the right to place their name first.

# Table of contents

**Supplementary Materials and Methods** ---------------------------------------------------------------- 1
**Supplementary Tables** ------------------------------------------------------------------------------------ 5
Table S1
Table S2
Table S3
Table S4
Table S5
Table S6
Table S7
Table S8
Table S9
Table S10
Table S11
Table S12
**Supplementary Figures** ---------------------------------------------------------------------------------- 30
Figure S1
Figure S2
Figure S3
Figure S4
Figure S5
Figure S6
Figure S7
Figure S8
Figure S9
Figure S10
Figure S11
Figure S12
Figure S13
Figure S14
Figure S15
Figure S16
Figure S17
Figure S18
Figure S19
Figure S20
Figure S21
Figure S22
Figure S23
Figure S24
Figure S25
**References** ------------------------------------------------------------------------------------------------ 63

## Supplementary Method

1. Design of synthetic bacterial small RNAs (sRNAs)

In this study, we designed sRNAs to modulate various riboregulators. The sRNA design can be categorized into two main approaches. First, de novo design, where novel sRNA sequences were created. Second, the cognate sRNA design to target reported riboregulators. For the de novo design, 15-nt RNA sequence was designed using NUPACK (1-3). In contrast, for the cognate sRNA design, sRNAs were designed to target the 15-nt region immediately upstream of the ribosome binding site (RBS).

A prominent example of de novo sRNA design is the orthogonal sRNA pool presented in **Figure 2f**. Below is the Python code used for designing the 5x5 orthogonal sRNA pool. Two major conditions were required throughout the design process. First, both the sRNA and its target must have linear structures to ensure efficient RNA-RNA interaction. Second, sRNA:target interaction was rewarded, while sRNA:nontarget interaction was penalized. The significance of these rewards and penalties was scaled square of the orthogonal pool size. This rigorous approach ensured the construction of a high-performance orthogonal sRNA pool.

```
# Import nupack python module
from nupack import *
import math

config.parallelism = True

# global pattern prevention
pattern = Pattern(['A4', 'C4', 'G4', 'U4', 'M6', 'K6', 'W6', 'S6', 'R6', 'Y6'])

# global constraints
div1 = Diversity(word=4, types=2)
div2 = Diversity(word=6, types=3)
# RNA, 37 Celsius
my_model = Model(material='rna', celsius=37)

#Toehold switch conserved sequence
toehold = Domain('AGTAAGATAATGAAG', name='toehold')
lowstem = Domain('GTAGGTATGTT', name='lowstem')
bulge = Domain('AAA', name='bulge')
upstem = Domain('CTTTA', name='upstem')
AUG = Domain('AUG', name='AUG')
RBS = Domain('AGAGGAGA', name='RBS')
#cognate trigger RNA
trigger = TargetStrand([~lowstem, ~toehold], name='trigger')

# number means the size of an orthogonal pool
# In this study, 5X5 orthogonal pool was designed.
number = 5
# The number of independent trials.
trial = 30
```

```
crosstalk_targets = {}
crosstalk_excludes = []
tubes = []

sRNA_strands = []
sRNA_structure_list = []
sRNA_target_structure_list = []
switch_strands = []

target_domains = []

for i in range(number):
target = Domain('N15', name=['target',i])

target_domains.append(target)

switch = TargetStrand([toehold, lowstem, bulge, upstem, target, RBS, ~upstem,AUG, ~lowstem],
name=['strand',i])
switch_strands.append(switch)

sRNA = TargetStrand([~target], name=['sRNA',i])
sRNA_target = TargetStrand([target], name=['sRNA target',i])
loop_strand = TargetStrand([target, RBS], name=['loop_strand', i])

sRNA_strands.append(sRNA)

#sRNA
sRNA_structure = TargetComplex([sRNA], '.15', name=['sRNA_c',i])

#sRNA target
sRNA_target_structure = TargetComplex([sRNA_target], '.15', name = ['sRNA_target_c', i])

#sRNA+sRNA target
sRNA_interaction = TargetComplex([sRNA, sRNA_target], '(15+)15', name = ['sRNA interaction',
i])

#Sw
switch_structure = TargetComplex([switch], '.15(11.3(5.23)5.3)11', name=['switch', i])

#Sw Tr
switch_open = TargetComplex([trigger, switch], '(26+)26.50', name=['switch_open', i])

#Sw Tr sRNA
switch_sRNA = TargetComplex([sRNA, trigger, switch], '(15+(26+)26.8)15.27',
name=['switch_sRNA', i])

sRNA_structure_list.append(sRNA_structure)
sRNA_target_structure_list.append(sRNA_target_structure)
```

```
tube = TargetTube(on_targets={switch_open: 1e-8, switch_structure: 1e-8},
off_targets=SetSpec(max_size=2, exclude=[[switch, sRNA]]), name=['repressed', i])
tubes.append(tube)


crosstalk_targets.update({sRNA_interaction: 1e-08})
crosstalk_excludes += [sRNA_interaction]

sRNA_crosstalk_tube = []
similarity = []
num=0
for i in range(number):
for m in range(number):
if i==m:
pass
else:
sim = Similarity(sRNA_strands[i], sRNA_strands[m], limits=[0,0.4], weight=number*number)
similarity.append(sim)
tube = TargetTube(on_targets={sRNA_structure_list[i]: 1e-8, sRNA_target_structure_list[m]: 1e-
8},
off_targets=SetSpec(max_size=2), name=['sRNA_crosstalk_tube', num])
```

2. Microplate reader analysis

Microplate reader was used to confirm the GFP fluorescence expression before conducting FACS analysis. Cell cultures of 200 µL were added per well on a 96-well Black Plate (Cat #. 33396, SPL, Gyeonggi-do, Korea) after an experiment. GFP fluorescence (excitation: 479 nm, emission: 520 nm) and OD600 were measured in a Synergy H1 microplate reader (BioTek Gen5, Santa Clara, CA, USA) running Gen5 3.08 software. GFP fluorescence levels were normalized as follows: fluorescence of LB blank was subtracted for background normalization, and the measured fluorescence value was divided by the OD600 value in the corresponding well. For **Figure S5**, where sRNA was expressed following toehold switch activation, measurements were taken at 5-minute intervals using the incubating function of a microplate reader. For all GFP expression experiments, the number of biological replicates was three.

3. RT-qPCR analysis

For the preparation of RNA samples, 1 mL of cell culture was collected for RNA extraction after the IPTG and arabinose induction. Total RNA was extracted using RiboEX (GeneAll, Seoul, Korea) in DNase/RNase-free condition. cDNA was synthesized with 1 µg of total RNA as a template using GoScriptTM Reverse Transcriptase with random primers (Promega, Madison, WI, USA) with the following conditions: Anneal primer - 25 °C for 5 min, Extension - 42 °C for 60 min, Inactivation – 70 °C for 15 min. Concentration of total RNA and cDNA was measured with a Synergy H1 microplate

reader. The 1 μg of cDNA was diluted 1/20-fold, then used for the quantitative PCR using ORATM qPCR Green ROX L MIX (highQu, Kraichtal, Germany) in Stratagene Mx3000P(Agilent Technologies, Santa Clara, CA, USA) with the following conditions: Initial denaturation - 95 °C for 2 min, Denaturation - 95 °C for 5 sec, Annealing/Extension - 60 °C for 20 sec. Amplicon length for target and normalizer gene is 107 bp and 340 bp, respectively. The mixture without template cDNA was used as a non-template control, and no unspecific Cq value was detected on non-template control. The number of replicates was three for each condition. All measurements were followed by melting curve analysis. Ct values were analyzed using MxPRO software (Agilent Technologies, Santa Clara, CA, USA).

ΔCt method is used to investigate the difference in the transcript amount. For ΔCt analysis, the GFP gene is selected as a target gene, and the 16S ribosomal RNA gene is selected as a normalizer gene. The normalized expression (ΔCt) were calculated as follows: ΔCt = Ct(GFP)-Ct(16S rRNA). Primer sequences used for the RT-qPCR analysis is described as followed: GFP forward primer (5'-TGGAAGCGTTCAACTAGCAG-3'), GFP reverse primer (5'- TCGAAAGGGCAGATTGTGTG-3'), 16S rRNA forward primer (5'-GTTAATACCTTTGCTCATTGA-3'), and 16S rRNA reverse primer (5'-ACCAGGGTATCTAATCCTGTT-3'). qPCR primers for GFP gene and 16S rRNA were imported from previous research (4). The specificity of primer pairs was assessed with Primer-BLAST, and no off-target matches were identified in NCBI Transcript Reference Sequences of *Escherichia coli*. DNA oligonucleotides of qPCR primers were purchased from Bionics (Seoul, Korea).

4. Kill switch preservation under frozen storage conditions

To evaluate the storage stability of the two-input kill switch, E. coli DH5α cells harboring the kill switch circuit were prepared as frozen stocks. Chemically transformed cells were plated on LB agar with appropriate antibiotics, and single colonies were grown overnight (~16 h) in 96-deep well plates (Cat# 503102, NEST, Wuxi, Jiangsu, China) with shaking at 800 rpm and 37°C. Overnight cultures were mixed 1:1 (v/v) with a formulation buffer consisting of $KH_2PO_4$ (4.56 g/L) and $K_2HPO_4$ (25 g/L) in 30% glycerol (pH 7.5). The mixtures were snap-frozen in liquid nitrogen and stored at -80°C.

For recovery, frozen samples were thawed on ice one day before the experiment and inoculated into pre-warmed LB medium containing appropriate antibiotics. The recovered cultures were grown overnight at 37°C and then mixed with healthy control strains at a 9:1 ratio. Subsequent procedures, including incubation, induction, and flow cytometry analysis, were conducted as described for the microbial co-culture experiment (**Figure 8**).

**Supplementary Table S1.** Plasmids used in this study. Abbreviations are as follows: T7term = T7 terminator, AmpR = ampicillin resistance gene, SpecR = spectinomycin resistance gene, KanR = kanamycin resistance gene, CmR = chloramphenicol resistance gene. All riboregulators, cognate triggers, and small bacterial RNAs were cloned into pET15b, pCOLADuet, pCDFDuet, or pACYCDuet plasmid.

| Name (plasmid) | Sequence |
|---|---|
| Bacterial small RNA (pET15b) | pT7 – sRNA – T7term – AmpR – pBR322 origin – LacI |
| Toehold switch (5) (pCOLADuet) | pJ23110 – lacO - Toehold switch – GFPmut3b – ssrA (ASV) – T7term – KanR – ColA origin – LacI |
| Cognate trigger (5) (pCDFDuet) | pLlacO-1 – Trigger RNA – T7term – SpecR – CloDF13 Origin – LacI |
| Theophylline riboswitch (6) (pCOLADuet) | pLlacO-1 – Theophylline riboswitch (ENYC3) – GFPmut3b – ssrA (ASV) – T7term – KanR – ColA origin – LacI |
| *Salmonella* 4U RNA thermometer (7) (pCOLADuet) | pLlacO-1 – *Salmonella* 4U RNA thermometer – GFPmut3b – ssrA (ASV) – T7term – KanR – ColA origin – LacI |
| START system: START–switch (8) (pCOLADuet) | pLlacO-1 – START switch – GFPmut3b – ssrA (ASV) – T7term – KanR – ColA origin – LacI |
| START system: (START–trigger) (8) (pCDFDuet) | pLlacO-1 – START trigger – T7term – SpecR – CloDF13 Origin – LacI |
| MS2 coat protein expression system (8) (pACYCDuet) | pLlacO – 8X His – MBP – MS2 coat protein – T7term – CmR – p15A origin – LacI |
| Holin encoding toehold switch (pCOLADuet) | pLlacO-1 - Toehold switch (with sRNA target) – Holin system [S gene, R gene, Rz gene] – T7term – KanR – ColA origin – LacI |

| | |
|---|---|
| Strong constitutive sRNA variant 1 (pET15b) | pTlpA – sRNA – T7term - AmpR – pBR322 origin – LacI |
| Temperature responsive sRNA system (pET15b) | pTlpA – sRNA – T7term – proA – TlpA36 (9) – T7term variant - AmpR – pBR322 origin – LacI |
| GFP positive (pACYCDuet) | pLtetO-1 - GFPmut3b – ssrA (ASV) – T7term – CmR – p15A origin – LacI |
| 3WJ repressor (10) (pCOLADuet) | pJ23110 – lacO – 3WJ – GFPmut3b – ssrA (ASV) – T7term - KanR – ColA origin – LacI |
| STAR (11,12) (pCOLADuet) | pLlacO-1 – STAR – GFPmut3b – ssrA (ASV) – T7term - KanR – ColA origin – LacI |
| Double Switch 1 (pCOLADuet) | pJ23110 – lacO – Orthogonal THS 1 – GFPmut3b – ssrA (ASV) – T7term – linker (134 nt) – pJ23110 – lacO – Orthogonal THS 4 – TagBFP – ssrA (ASV) – T7term - KanR – ColA origin – LacI |
| Toehold switch with BFP (pCOLADuet) | pJ23110 – lacO – THS – TagBFP – ssrA (ASV) – T7term - KanR – ColA origin – LacI |
| pLux THS ECF11 (pCOLADuet) | pJ23110 – lacO – THS – coupling element - Ecf11 – ssrA (AAV) – T7term - KanR – ColA origin – LacI |
| pLux trigger (pET15b) | pLac – RBS (tttaactttaagaaggagatatacat) – LuxR - ssrA (ASV)– bidirectional terminator – linker (100 nt) – pLux – Trigger RNA – T7term – AmpR – pBR322 origin – LacI |
| pECF GFP (pACYCDuet) | pECF – 5' UTR with RBS (ACCTAAGGTAAATAAGGAGGAGTAAC) – GFPmut3b - ssrA (ASV) – T7term – CmR – p15A origin – LacI |
| Double sRNA 1 (pET15b) | pT7 – Orthogonal sRNA 1 – T7term – linker (143 nt) – pT7 – Orthogonal sRNA 4 – T7term – AmpR – pBR322 origin – LacI |
| Double sRNA 2 (pET15b) | pT7 – sRNA variant 2 – T7term – linker (143 nt) – pT7 – sRNA variant 3 – T7term – AmpR – pBR322 origin – LacI |
| pLlacO GFP (pCOLADuet) | pLlacO-1 – 3WJ – GFPmut3b - ssrA (ASV) – T7term - KanR – ColA origin – LacI |
| pTtgR GFP (pJKR-H) | pTtgR – 3WJ - GFPmut3b - ssrA (ASV) – bidirectional terminator – linker (17 nt) – proA – TtgR - T7term – AmpR – pUC origin – rrnB terminator |

| | |
|---|---|
| From addgene # 62565 | |
| pLux GFP<br>(pET15b)<br>From addgene #172570 | pLux – 3WJ - GFPmut3b - ssrA (ASV) - T7term – f1 origin – AmpR – pBR322 origin – LuxR |

**Supplementary Table S2.** General genetic components used in the main figures. The sequences of other riboregulators can be found in **Table S5, S6, S7 and S9**.

| Name | Sequence |
|---|---|
| T7 promoter | TAATACGACTCACTATAGGG |
| pJ23110 | TTTACGGCTAGCTCAGTCCTAGGTACAATGCTAGC |
| pLlacO-1 | ATAAATGTGAGCGGATAACATTGACATTGTGAGCGGATAACAAGATACTGAGCAC |
| pLtetO-1 | TCCCTATCAGTGATAGAGATTGACATCCCTATCAGTGATAGATATACTGAGCAC |
| pTlpA | TTTAATTTGTTTGTTAGTTAGTTTATTTGTTGGTTTGTTTGTGTTATAATAT |
| proA | CACAGCTAACACCACGTCGTCCCTATCTGCTGCCCTAGGTCTATGAGTGGTTGCTGGATAACTTTACGGGCATGCATAAGGCTCGTAGGCTATATTCAGGGAGACCACAACGGTTTCCCTCTACAAATAATTTTGTTTAACTTT |
| pLac | ATAAATGTGAGCGGATAACATTGACATTGTGAGCGGATAACAAGATACTGAGCAC |
| pLux | AGACCTGTAGGATCGTACAGGTTTACGCAAGAAAATGGTTTGTTACTTTCGAATAAA |
| pECF | GCCTCCACACCGCTCGTCACATCCTGTGATCCACTCTTCATCCCGCTACGTAACACCTCTGCATCGCGAACCAAAACCAG |
| pTtgR | CACCCAGCAGTATTTACAAACAACCATGAATGTAAGTATATTCCTTAGCAA |
| Bidirectional terminator | AAATATAATGACCCTCTTGATAACCCAAGAGGGCATTTTTTA |
| Lac operator | AATTGTGAGCGGATAACAATT |
| T7 terminator | TAGCATAACCCCTTGGGGCCTCTAAACGGGTCTTGAGGGGTTTTTTG |
| T7 terminator variant | TAGCATAAACAGATAGGCCCTCTTCGGAGGGCCTATCTGTTTTTTTTTG |
| sRNA (**target sequence** – Hfq scaffold – rho independent transcription terminator) | NNNNNNNNNNNNNNNATTTGTAGAAATATTTTATTCGCCCCCGGAAGATCATTCCGGGGGCTTTTTTATT |
| Non-targeting sRNA (Hfq scaffold – rho independent transcription terminator) | ATTTGTAGAAATATTTTATTCGCCCCCGGAAGATCATTCCGGGGGCTTTTTTATT |
| Toehold switch (sRNA target, **RBS**) | AGTAAGATAATGAAGGTAGGTATGTTAAACTTTANNNNNNNNNNNNNNN**AGAGGAGA**TAAAGATGAACATACCTACGAACCTGGCGGCAGCGCAAAAG |

| | |
|---|---|
| Cognate trigger | GCTCGATCACTAATCTGATCGAGACGAACATACCTACCTTCATTATCTTACTTGT |
| Decoy RNA | TCTCACGCCCTCAGCTGGGCGTGAGATGAGCCTCGTCTCCAGATGACGAGGCAACGTAGGA<br>TCTGACTGATCCTACTAT |
| ssrA(ASV) | AGGCCTGCAGCAAACGACGAAAACTACGCTGCATCAGTTTAATAA |
| ssrA(AAV) | AGGCCTGCAGCAAACGACGAAAACTACGCTGCTGCTGTT |
| GFPmut3b | ATGCGTAAAGGAGAAGAACTTTTCACTGGAGTTGTCCCAATTCTTGTTGAATTAGATGGTG<br>ATGTTAATGGGCACAAATTTTCTGTCAGTGGAGAGGGTGAAGGTGATGCAACATACGGAA<br>AACTTACCCTTAAATTTATTTGCACTACTGGAAAACTACCTGTTCCGTGGCCAACACTTGTC<br>ACTACTTTCGGTTATGGTGTTCAATGCTTTGCGAGATACCCAGATCACATGAAACAGCATG<br>ACTTTTTCAAGAGTGCCATGCCCGAAGGTTACGTACAGGAAAGAACTATATTTTTCAAAGA<br>TGACGGGAACTACAAGACACGTGCTGAAGTCAAGTTTGAAGGTGATACCCTTGTTAATAGA<br>ATCGAGTTAAAAGGTATTGATTTTAAAGAAGATGGAAACATTCTTGGACACAAATTGGAAT<br>ACAACTATAACTCACACAATGTATACATCATGGCAGACAAACAAAAGAATGGAATCAAAG<br>TTAACTTCAAAATTAGACACAACATTGAAGATGGAAGCGTTCAACTAGCAGACCATTATCA<br>ACAAAATACTCCGATTGGCGATGGCCCTGTCCTTTTACCAGACAACCATTACCTGTCCACA<br>CAATCTGCCCTTTCGAAAGATCCCAACGAAAAGAGAGACCACATGGTCCTTCTTGAGTTTG<br>TAACCGCTGCTGGGATTACACATGGCATGGATGAACTATACAAA |
| TlpA36 | ATGCGTCCGGCGACATACGAACCAGAACAGATTATTGAAGCAGGGCTGGCCCTGCAGGCT<br>GAAGGACGGAATATCACCGGGTTCGCACTACGTAACCAGGTGGGTGGCGGCAATCCGACA<br>CGTCTCCGCCAGATATGGGACGAATACCAGGCTTCACAGAGCACGGTCGTCACTGAACTCG<br>TTGCCGAGCTGCCAGTGGAAGTGGCTGAAGAAGTGAAGGCCGTCTCCGCCGCGCTGTCCG<br>AACGCATCACCCAGCTGGCGACAGAACTGAATGACAAGGCGGTCCGGGCTGCAGAACGCC<br>GGGTTGCGGAAGTCACGCGTGCTGCCGGTGAACAGACCGCACAGGCAGAGCGGGAGCTGG<br>CCGACGCCGCGCAGACAGTCGACGACCTGGAAGAAAAACTGGTTGAACTGCAGGACAGAT<br>ATGACAGTTTGACGCTGGCGCTGGAGTCAGAACGTTCACTGCGTCAGCAGCATGATGTGGA<br>GATGGCCCAGCTGAAAGAGCGTCTTGCGGCCGCTGAAGAGAATACCCGTCAGCGAGAGGA<br>ACGGTATCAGGAGCAGAGGACAGTGCTGCAGGATGCGCTTAATGCGGAGCAGGCACAGCA<br>CATAAACACGCGGGAAGACCAGCAGAAACGACTGGAGCAAATTTCTGCCGAAGCTAATGC<br>GCGTACAGAAGAACTGAAGTCTGAACGCGATAAAGTCAATACTCTCCTTACCCGCCTTGAA<br>TCGCAGGAAAATGCGCTGGCCTCAGAACGTCAGCAGCATCTGGCCACCCGCGAAACGCTG<br>CAGCAACGCCTCGAGCAGGCCATCGCTGACACGCAGGCGCGCGCCGGTGAGATTGCACTT<br>GAACGTGACAGAGTCAGCAGCCTCACCGCAAGGCTGGAATCGCAGGAAAAGGCCTCCTCG<br>GAGCAACTGGTGCGTATGGGCAGTGAAATAGCCAGTCTGACAGAGCGTTGCACACAGCTG<br>GAAAACCAGCGTGATGATGCCCGTCTGGAGACGATGGGGGAGAAAGAAACGGTCGCGGC<br>ACTGCGTGGTGAGGCTGAAGCCCTGAAGCGTCAGAACCAGTCACTGATGGCGGCGCTTTCA<br>GGCAATAAACAGACCGGTGGCCAGAATGCGTGA |
| coupling element | ACACTCGCAGAGGAGAGCGAGTA |
| S gene<br>(Holin complex component) | ATGCCAGAAAAACATGACCTGTTGGCCGCCATTCTCGCGGCAAAGGAACAAGGCATCGGGG<br>CAATCCTTGCGTTTGCAATGGCGTACCTTCGCGGCAGATATAATGGCGGTGCGTTTACAAAA<br>ACAGTAATCGACGCAACGATGTGCGCCATTATCGCCTGGTTCATTCGTGACCTTCTCGACTTC<br>GCCGGACTAAGTAGCAATCTCGCTTATATAACGAGCGTGTTTATCGGCTACATCGGTACTGAC<br>TCGATTGGTTCGCTTATCAAACGCTTCGCTGCTAAAAAAGCCGGAGTAGAAGATGGTAGAAA<br>TCAATAA |
| *R gene*<br>(Holin complex component) | *ATGGTAGAAATCAATAATCAACGTAAGGCGTTCCTCGATATGCTGGCGTGGTCGGAGGGAACTGAT*<br>*AACGGACGTCAGAAAACCAGAAATCATGGTTATGACGTCATTGTAGGCGGAGAGCTATTTACTGAT*<br>*TACTCCGATCACCCTCGCAAACTTGTCACGCTAAACCCAAAACTCAAATCAACAGGCGCCGGACG*<br>*CTACCAGCTTCTTTCCCGTTGGTGGGATGCCTACCGCAAGCAGCTTGGCCTGAAAGACTTCTCTC*<br>*CGAAAAGTCAGGACGCTGTGGCATTGCAGCAGATTAAGGAGCGTGGCGCTTTACCTATGATTGAT*<br>*CGTGGTGATATCCGTCAGGCAATCGACCGTTGCAGCAATATCTGGGCTTCACTGCCGGGCGCTG*<br>*GTTATGGTCAGTTCGAGCATAAGGCTGACAGCCTGATTGCAAAATTCAAAGAAGCGGGCGGAACG*<br>*GTCAGAGAGATTGATGTATGA* |
| Rz gene<br>(Holin complex | ATGAGCAGAGTCACCGCGATTATCTCCGCTCTGGTTATCTGCATCATCGTCTGCCTGTCATGG<br>GCTGTTAATCATTACCGTGATAACGCCATTACCTACAAAGCCCAGCGCGACAAAAATGCCAG<br>AGAACTGAAGCTGGCGAACGCGGCAATTACTGACATGCAGATGCGTCAGCGTGATGTTGCT<br>GCGCTCGATGCAAAATACACGAAGGAGTTAGCTGATGCTAAAGCTGAAAATGATGCTCTGCG<br>TGATGATGTTGCCGCTGGTCGTCGTCGGTTGCACATCAAAGCAGTCTGTCAGTCAGTGCGTG |

| | |
|---|---|
| component) | AAGCCACCACCGCCTCCGGCGTGGATAATGCAGCCTCCCCCCGACTGGCAGACACCGCTGA<br>ACGGGATTATTTCACCCTCAGAGAGAGGCTGATCACTATGCAAAAACAACTGGAAGGAACC<br>CAGAAGTATATTAATGAGCAGTGCAGATAG |
| LuxR | ATGAAAAACATAAATGCCGACGACACATACAGAATAATTAATAAAATTAAAGCTTGTAGAAG<br>CAATAATGATATTAATCAATGCTTATCTGATATGACTAAAATGGTACATTGTGAATATTATTTAC<br>TCGCGATCATTTATCCTCATTCTATGGTTAAATCTGATATTTCAATCCTAGATAATTACCCTAAA<br>AAATGGAGGCAATATTATGATGACGCTAATTTAATAAAATATGATCCTATAGTAGATTATTCTAA<br>CTCCAATCATTCACCAATTAATTGGAATATATTTGAAAACAATGCTGTAAATAAAAAATCTCC<br>AAATGTAATTAAAGAAGCGAAAACATCAGGTCTTATCACTGGGTTTAGTTTCCCTATTCATAC<br>GGCTAACAATGGCTTCGGAATGCTTAGTTTTGCACATTCAGAAAAAGACAACTATATAGATA<br>GTTTATTTTTACATGCGTGTATGAACATACCATTAATTGTTCCTTCTCTAGTTGATAATTATCGA<br>AAAATAAATATAGCAAATAATAAATCAAACAACGATTTAACCAAAAGAGAAAAAGAATGTTT<br>AGCGTGGGCATGCGAAGGAAAAAGCTCTTGGGATATTTCAAAAATATTAGGTTGCAGTGAGC<br>GTACTGTCACTTTCCATTTAACCAATGCGCAAATGAAACTCAATACAACAAACCGCTGCCAA<br>AGTATTTCTAAAGCAATTTTAACAGGAGCAATTGATTGCCCATACTTTAAAAAT |
| Ecf11 | ATGGGCAGCAGCCATCATCATCATCATCACAGCAGCGGCCTGGAAGTTCTGTTCCAGGGGCC<br>CCATATGATGAGCGATAGTCCGCAGAAACTGGGTCGTAATGAATGGAATGCCTATATGGATAA<br>AGTGAAAGCCAAAGATCGTGAAGCCTTTGCATTTGTGTTTCGTTTTTATGCACCGAAACTGA<br>AACAGTTCGCCTATAAACATGTGGGTAATGAACAGGTTGCCATGGAAATGGTTCAAGAAACC<br>ATGGCCACCGTTTGGCAGAAAGCACATCTGTATGATGGTAAAAAAAGCGCACTGAGCACCT<br>GGATTTATACCATTATTCGCAACCTGTGCTTTGATCTGCTGCGTAAACAGAAAGGTAAAGAAC<br>TGCATATCCACTCCGATGATATTTGGCCGAGCGAATATTATCCGCCTGATATGGTTGATCACTA<br>TAGTCCGGAACAGGATATGCTGAAAGAACAGGTGGTGAAATTTCTGGATATCCTGCCGAAAA<br>ATCAGCGTGATGTTCTGCAGGCAGTTTATCTGGAAGAACTGCCGCATCAGCAGGTTGCAGAA<br>CTGTTTGATATTCCGCTGGGCACCGTTAAAAGCCGTCTGCGTCTGGCAGTTGAAAAACTGCG<br>TCATAGCATGCATACCGAACAGCTG |
| TtgR | ATGGTGCGTCGCACCAAAGAAGAAGCACAGGAAACGCGTGCGCAGATTATCGAAGCGGCC<br>GAACGCGCGTTTTATAAACGTGGTGTGGCACGTACCACGCTGGCAGATATTGCAGAACTGGC<br>AGGTGTTACCCGCGGTGCAATCTACTGGCATTTCAACAATAAAGCCGAACTGGTTCAGGCAC<br>TGCTGGATTCTCTGCACGAAACGCATGATCACCTGGCCCGTGCAAGCGAATCTGAAGATGA<br>ACTGGACCCGCTGGGCTGCATGCGCAAACTGCTGCTGCAGGTGTTTAACGAACTGGTTCTG<br>GATGCACGTACCCGTCGCATTAATGAAATCCTGCATCACAAATGCGAATTTACGGATGATATG<br>TGTGAAATTCGTCAGCAGCGCCAGAGCGCCGTGCTGGATTGTCATAAAGGTATCACCCTGGC<br>ACTGGCAAACGCAGTTCGTCGCGGTCAGCTGCCGGGTGAACTGGATGTGGAACGCGCAGC<br>GGTTGCGATGTTTGCCTATGTGGATGGCCTGATTGGTCGTTGGCTGCTGCTGCCGGATAGTGT<br>TGACCTGCTGGGCGATGTGGAAAAATGGGTTGATACCGGTCTGGATATGCTGCGTCTGAGCC<br>CGGCGCTGCGCAAATAA |

**Supplementary Table S3.** Examples of DNA plasmid sequences. Origin of replication and LacI has been reversed to other elements.

| Name<br>[plasmid]<br>(architecture) | Sequence |
|---|---|
| Bacterial small RNA variant 1<br><br>[pET15b]<br><br>(pT7 – **target sequence** – Hfq scaffold – rho independent transcription terminator – T7term – AmpR – pBR322 origin – LacI) | AACGTGTACGGGCTATCTGGCTTTCGTTGCGCTAATACGACTCACTATAGG**GTTCT**<br>**TGTCTTGTTC**ATTTGTAGAAATATTTTATTCGCCCCCGGAAGATCATTCCGGGGGC<br>TTTTTTATTTAGCATAACCCCTTGGGGCCTCTAAACGGGTCTTGAGGGGTTTTTTGC<br>TGAAAGGAGGAACTATATCCGGATATCCCGCAAGAGGCCCGGCAGTACCGGCATA<br>ACCAAGCCTATGCCTACAGCATCCAGGGTGACGGTGCCGAGGATGACGATGAGCG<br>CATTGTTAGATTTCATACACGGTGCCTGACTGCGTTAGCAATTTAACTGTGATAAA<br>CTACCGCATTAAAGCTTATCGATGATAAGCTGTCAAACATGAGAATTCTTGAAGAC<br>GAAAGGGCCTCGTGATACGCCTATTTTTATAGGTTAATGTCATGATAATAATGGTT<br>TCTTAGACGTCAGGTGGCACTTTTCGGGGAAATGTGCGCGGAACCCCTATTTGTTT<br>ATTTTTCTAAATACATTCAAATATGTATCCGCTCATGAGACAATAACCCTGATAAA<br>TGCTTCAATAATATTGAAAAAGGAAGAGTATGAGTATTCAACATTTCCGTGTCGCC<br>CTTATTCCCTTTTTTGCGGCATTTTGCCTTCCTGTTTTTGCTCACCCAGAAACGCTGG<br>TGAAAGTAAAAGATGCTGAAGATCAGTTGGGTGCACGAGTGGGTTACATCGAACT<br>GGATCTCAACAGCGGTAAGATCCTTGAGAGTTTTCGCCCCGAAGAACGTTTTCCAA<br>TGATGAGCACTTTTAAAGTTCTGCTATGTGGCGCGGTATTATCCCGTGTTGACGCC<br>GGGCAAGAGCAACTCGGTCGCCGCATACACTATTCTCAGAATGACTTGGTTGAGTA<br>CTCACCAGTCACAGAAAAGCATCTTACGGATGGCATGACAGTAAGAGAATTATGC<br>AGTGCTGCCATAACCATGAGTGATAACACTGCGGCCAACTTACTTCTGACAACGAT<br>CGGAGGACCGAAGGAGCTAACCGCTTTTTTGCACAACATGGGGGATCATGTAACT<br>CGCCTTGATCGTTGGGAACCGGAGCTGAATGAAGCCATACCAAACGACGAGCGTG<br>ACACCACGATGCCTGCAGCAATGGCAACAACGTTGCGCAAACTATTAACTGGCGA<br>ACTACTTACTCTAGCTTCCCGGCAACAATTAATAGACTGGATGGAGGCGGATAAAG<br>TTGCAGGACCACTTCTGCGCTCGGCCCTTCCGGCTGGCTGGTTTATTGCTGATAAAT<br>CTGGAGCCGGTGAGCGTGGGTCTCGCGGTATCATTGCAGCACTGGGGCCAGATGG<br>TAAGCCCTCCCGTATCGTAGTTATCTACACGACGGGGAGTCAGGCAACTATGGATG<br>AACGAAATAGACAGATCGCTGAGATAGGTGCCTCACTGATTAAGCATTGGTAACT<br>GTCAGACCAAGTTTACTCATATATACTTTAGATTGATTTAAAACTTCATTTTTAATT<br>TAAAAGGATCTAGGTGAAGATCCTTTTTGATAATCTCATGACCAAAATCCCTTAAC<br>GTGAGTTTTCGTTCCACTGAGCGTCAGACCCCGTAGAAAAGATCAAAGGATCTTCT<br>TGAGATCCTTTTTTTCTGCGCGTAATCTGCTGCTTGCAAACAAAAAAACCACCGCT<br>ACCAGCGGTGGTTTGTTTGCCGGATCAAGAGCTACCAACTCTTTTTCCGAAGGTAA<br>CTGGCTTCAGCAGAGCGCAGATACCAAATACTGTCCTTCTAGTGTAGCCGTAGTTA<br>GGCCACCACTTCAAGAACTCTGTAGCACCGCCTACATACCTCGCTCTGCTAATCCT<br>GTTACCAGTGGCTGCTGCCAGTGGCGATAAGTCGTGTCTTACCGGGTTGGACTCAA<br>GACGATAGTTACCGGATAAGGCGCAGCGGTCGGGCTGAACGGGGGGTTCGTGCAC<br>ACAGCCCAGCTTGGAGCGAACGACCTACACCGAACTGAGATACCTACAGCGTGAG<br>CTATGAGAAAGCGCCACGCTTCCCGAAGGGAGAAAGGCGGACAGGTATCCGGTAA<br>GCGGCAGGGTCGGAACAGGAGAGCGCACGAGGGAGCTTCCAGGGGGAAACGCCT<br>GGTATCTTTATAGTCCTGTCGGGTTTCGCCACCTCTGACTTGAGCGTCGATTTTTGT<br>GATGCTCGTCAGGGGGGCGGAGCCTATGGAAAAACGCCAGCAACGCGGCCTTTTT<br>ACGGTTCCTGGCCTTTTGCTGGCCTTTTGCTCACATGTTCTTTCCTGCGTTATCCCCT<br>GATTCTGTGGATAACCGTATTACCGCCTTTGAGTGAGCTGATACCGCTCGCCGCAG<br>CCGAACGACCGAGCGCAGCGAGTCAGTGAGCGAGGAAGCGGAAGAGCGCCTGAT<br>GCGGTATTTTCTCCTTACGCATCTGTGCGGTATTTCACACCGCATATATGGTGCACT<br>CTCAGTACAATCTGCTCTGATGCCGCATAGTTAAGCCAGTATACACTCCGCTATCG<br>CTACGTGACTGGGTCATGGCTGCGCCCCGACACCCGCCAACACCCGCTGACGCGCC<br>CTGACGGGCTTGTCTGCTCCCGGCATCCGCTTACAGACAAGCTGTGACCGTCTCCG<br>GGAGCTGCATGTGTCAGAGGTTTTCACCGTCATCACCGAAACGCGCGAGGCAGCT<br>GCGGTAAAGCTCATCAGCGTGGTCGTGAAGCGATTCACAGATGTCTGCCTGTTCAT<br>CCGCGTCCAGCTCGTTGAGTTTCTCCAGAAGCGTTAATGTCTGGCTTCTGATAAAG<br>CGGGCCATGTTAAGGGCGGTTTTTTCCTGTTTGGTCACTGATGCCTCCGTGTAAGG<br>GGGATTTCTGTTCATGGGGGTAATGATACCGATGAAACGAGAGAGGATGCTCACG<br>ATACGGGTTACTGATGATGAACATGCCCGGTTACTGGAACGTTGTGAGGGTAAAC<br>AACTGGCGGTATGGATGCGGCGGGACCAGAGAAAAATCACTCAGGGTCAATGCCA<br>GCGCTTCGTTAATACAGATGTAGGTGTTCCACAGGGTAGCCAGCAGCATCCTGCGA<br>TGCAGATCCGGAACATAATGGTGCAGGGCGCTGACTTCCGCGTTTCCAGACTTTAC |

| | |
|---|---|
| | GAAACACGGAAACCGAAGACCATTCATGTTGTTGCTCAGGTCGCAGACGTTTTGCA<br>GCAGCAGTCGCTTCACGTTCGCTCGCGTATCGGTGATTCATTCTGCTAACCAGTAA<br>GGCAACCCCGCCAGCCTAGCCGGGTCCTCAACGACAGGAGCACGATCATGCGCAC<br>CCGTGGCCAGGACCCAACGCTGCCCGAGATGCGCCGCGTGCGGCTGCTGGAGATG<br>GCGGACGCGATGGATATGTTCTGCCAAGGGTTGGTTTGCGCATTCACAGTTCTCCG<br>CAAGAATTGATTGGCTCCAATTCTTGGAGTGGTGAATCCGTTAGCGAGGTGCCGCC<br>GGCTTCCATTCAGGTCGAGGTGGCCCGGCTCCATGCACCGCGACGCAACGCGGGG<br>AGGCAGACAAGGTATAGGGCGGCGCCTACAATCCATGCCAACCCGTTCCATGTGC<br>TCGCCGAGGCGGCATAAATCGCCGTGACGATCAGCGGTCCAGTGATCGAAGTTAG<br>GCTGGTAAGAGCCGCGAGCGATCCTTGAAGCTGTCCCTGATGGTCGTCATCTACCT<br>GCCTGGACAGCATGGCCTGCAACGCGGGCATCCCGATGCCGCCGGAAGCGAGAAG<br>AATCATAATGGGGAAGGCCATCCAGCCTCGCGTCGCGAACGCCAGCAAGACGTAG<br>CCCAGCGCGTCGGCCGCCATGCCGGCGATAATGGCCTGCTTCTCGCCGAAACGTTT<br>GGTGGCGGGACCAGTGACGAAGGCTTGAGCGAGGGCGTGCAAGATTCCGAATACC<br>GCAAGCGACAGGCCGATCATCGTCGCGCTCCAGCGAAAGCGGTCCTCGCCGAAAA<br>TGACCCAGAGCGCTGCCGGCACCTGTCCTACGAGTTGCATGATAAAGAAGACAGT<br>CATAAGTGCGGCGACGATAGTCATGCCCCGCGCCCACCGGAAGGAGCTGACTGGG<br>TTGAAGGCTCTCAAGGGCATCGGTCGAGATCCCGGTGCCTAATGAGTGAGCTAACT<br>TACATTAATTGCGTTGCGCTCACTGCCCGCTTTCCAGTCGGGAAACCTGTCGTGCC<br>AGCTGCATTAATGAATCGGCCAACGCGCGGGGAGAGGCGGTTTGCGTATTGGGCG<br>CCAGGGTGGTTTTTCTTTTCACCAGTGAGACGGGCAACAGCTGATTGCCCTTCACC<br>GCCTGGCCCTGAGAGAGTTGCAGCAAGCGGTCCACGCTGGTTTGCCCCAGCAGGC<br>GAAAATCCTGTTTGATGGTGGTTAACGGCGGGATATAACATGAGCTGTCTTCGGTA<br>TCGTCGTATCCCACTACCGAGATATCCGCACCAACGCGCAGCCCGGACTCGGTAAT<br>GGCGCGCATTGCGCCCAGCGCCATCTGATCGTTGGCAACCAGCATCGCAGTGGGA<br>ACGATGCCCTCATTCAGCATTTGCATGGTTTGTTGAAAACCGGACATGGCACTCCA<br>GTCGCCTTCCCGTTCCGCTATCGGCTGAATTTGATTGCGAGTGAGATATTTATGCCA<br>GCCAGCCAGACGCAGACGCGCCGAGACAGAACTTAATGGGCCCGCTAACAGCGCG<br>ATTTGCTGGTGACCCAATGCGACCAGATGCTCCACGCCCAGTCGCGTACCGTCTTC<br>ATGGGAGAAAATAATACTGTTGATGGGTGTCTGGTCAGAGACATCAAGAAATAAC<br>GCCGGAACATTAGTGCAGGCAGCTTCCACAGCAATGGCATCCTGGTCATCCAGCG<br>GATAGTTAATGATCAGCCCACTGACGCGTTGCGCGAGAAGATTGTGCACCGCCGCT<br>TTACAGGCTTCGACGCCGCTTCGTTCTACCATCGACACCACCACGCTGGCACCCAG<br>TTGATCGGCGCGAGATTTAATCGCCGCGACAATTTGCGACGGCGCGTGCAGGGCC<br>AGACTGGAGGTGGCAACGCCAATCAGCAACGACTGTTTGCCCGCCAGTTGTTGTGC<br>CACGCGGTTGGGAATGTAATTCAGCTCCGCCATCGCCGCTTCCACTTTTTCCCGCGT<br>TTTCGCAGAAACGTGGCTGGCCTGGTTCACCACGCGGGAAACGGTCTGATAAGAG<br>ACACCGGCATACTCTGCGACATCGTATAACGTTACTGGTTTCACATTCACCACCCT<br>GAATTGACTCTCTTCCGGGCGCTATCATGCCATACCGCGAAAGGTTTTGCGCCATT<br>CGATGGTGTCCGGGATCTCGACGCTCTCCCTTATGCGACTCCTGCATTAGGAAGCA<br>GCCCAGTAGTAGGTTGAGGCCGTTGAGCACCGCCGCCGCAAGGAATGGTGCATGC<br>AAGGAGATGGCGCCCAACAGTCCCCCGGCCACGGGGCCTGCCACCATACCCACGC<br>CGAAACAAGCGCTCATGAGCCCGAAGTGGCGAGCCCGATCTTCCCCATCGGTGAT<br>GTCGGCGATATAGGCGCCAGCAACCGCACCTGTGGCGCCGGTGATGCCGGCCACG<br>ATGCGTCCGGCGTAGAGGATCGAGATCTCG |
| Toehold switch<br><br>[pCOLADuet]<br><br>(pJ23110 – lacO - Toehold switch (sRNA target, **RBS**) – GFPmut3b - ssrA(ASV) – T7term – KanR – ColA origin – LacI) | CGGGATCTCGACGCTCTCCCTTATGAAGTCTAACGCTGCTCTGGGCTAACTGTCGC<br>GCTTTACGGCTAGCTCAGTCCTAGGTACAATGCTAGCAATTGTGAGCGGATAACAA<br>TTAGTAAGATAATGAAGGTAGGTATGTTAAACTTTAGAACAAGACAAGAAC**AGAG**<br>**GAGA**TAAAGATGAACATACCTACGAACCTGGCGGCAGCGCAAAAGATGCGTAAA<br>GGAGAAGAACTTTTCACTGGAGTTGTCCCAATTCTTGTTGAATTAGATGGTGATGT<br>TAATGGGCACAAATTTTCTGTCAGTGGAGAGGGTGAAGGTGATGCAACATACGGA<br>AAACTTACCCTTAAATTTATTTGCACTACTGGAAAACTACCTGTTCCGTGGCCAAC<br>ACTTGTCACTACTTTCGGTTATGGTGTTCAATGCTTTGCGAGATACCCAGATCACAT<br>GAAACAGCATGACTTTTTCAAGAGTGCCATGCCCGAAGGTTACGTACAGGAAAGA<br>ACTATATTTTTCAAAGATGACGGGAACTACAAGACACGTGCTGAAGTCAAGTTTGA<br>AGGTGATACCCTTGTTAATAGAATCGAGTTAAAAGGTATTGATTTTAAAGAAGATG<br>GAAACATTCTTGGACACAAATTGGAATACAACTATAACTCACACAATGTATACATC<br>ATGGCAGACAAACAAAAGAATGGAATCAAAGTTAACTTCAAAATTAGACACAACA<br>TTGAAGATGGAAGCGTTCAACTAGCAGACCATTATCAACAAAATACTCCGATTGGC<br>GATGGCCCTGTCCTTTTACCAGACAACCATTACCTGTCCACACAATCTGCCCTTTCG<br>AAAGATCCCAACGAAAAGAGAGACCACATGGTCCTTCTTGAGTTTGTAACCGCTGC<br>TGGGATTACACATGGCATGGATGAACTATACAAAAGGCCTGCAGCAAACGACGAA<br>AACTACGCTGCATCAGTTTAATAATAATAAGATAAACCAGAGCGGCACGGCAAGC |

| | |
|---|---|
| | AGAGTATACGAGATTCGGTAGCCACCGCTGAGCAATAACTAGCATAACCCCTTGG<br>GGCCTCTAAACGGGTCTTGAGGGGTTTTTTGCTGAAACCTCAGGCATTTGAGAAGC<br>ACACGGTCACACTGCTTCCGGTAGTCAATAAACCGGTAAACCAGCAATAGACATA<br>AGCGGCTATTTAACGACCCTGCCCTGAACCGACGACAAGCTGACGACCGGGTCTCC<br>GCAAGTGGCACTTTTCGGGGAAATGTGCGCGGAACCCCTATTTGTTTATTTTTCTAA<br>ATACATTCAAATATGTATCCGCTCATGAATTAATTCTTAGAAAAACTCATCGAGCA<br>TCAAATGAAACTGCAATTTATTCATATCAGGATTATCAATACCATATTTTTGAAAA<br>AGCCGTTTCTGTAATGAAGGAGAAAACTCACCGAGGCAGTTCCATAGGATGGCAA<br>GATCCTGGTATCGGTCTGCGATTCCGACTCGTCCAACATCAATACAACCTATTAAT<br>TTCCCCTCGTCAAAAATAAGGTTATCAAGTGAGAAATCACCATGAGTGACGACTGA<br>ATCCGGTGAGAATGGCAAAAGTTTATGCATTTCTTTCCAGACTTGTTCAACAGGCC<br>AGCCATTACGCTCGTCATCAAAATCACTCGCATCAACCAAACCGTTATTCATTCGT<br>GATTGCGCCTGAGCGAGACGAAATACGCGGTCGCTGTTAAAAGGACAATTACAAA<br>CAGGAATCGAATGCAACCGGCGCAGGAACACTGCCAGCGCATCAACAATATTTTC<br>ACCTGAATCAGGATATTCTTCTAATACCTGGAATGCTGTTTTCCCGGGGATCGCAG<br>TGGTGAGTAACCATGCATCATCAGGAGTACGGATAAAATGCTTGATGGTCGGAAG<br>AGGCATAAATTCCGTCAGCCAGTTTAGTCTGACCATCTCATCTGTAACATCATTGG<br>CAACGCTACCTTTGCCATGTTTCAGAAACAACTCTGGCGCATCGGGCTTCCCATAC<br>AATCGATAGATTGTCGCACCTGATTGCCCGACATTATCGCGAGCCCATTTATACCC<br>ATATAAATCAGCATCCATGTTGGAATTTAATCGCGGCCTAGAGCAAGACGTTTCCC<br>GTTGAATATGGCTCATACTCTTCCTTTTTCAATATTATTGAAGCATTTATCAGGGTT<br>ATTGTCTCATGAGCGGATACATATTTGAATGTATTTAGAAAAATAAACAAATAGGC<br>ATGCTAGCGCAGAAACGTCCTAGAAGATGCCAGGAGGATACTTAGCAGAGAGACA<br>ATAAGGCCGGAGCGAAGCCGTTTTTCCATAGGCTCCGCCCCCCTGACGAACATCAC<br>GAAATCTGACGCTCAAATCAGTGGTGGCGAAACCCGACAGGACTATAAAGATACC<br>AGGCGTTTCCCCCTGATGGCTCCCTCTTGCGCTCTCCTGTTCCCGTCCTGCGGCGTC<br>CGTGTTGTGGTGGAGGCTTTACCCAAATCACCACGTCCCGTTCCGTGTAGACAGTT<br>CGCTCCAAGCTGGGCTGTGTGCAAGAACCCCCCGTTCAGCCCGACTGCTGCGCCTT<br>ATCCGGTAACTATCATCTTGAGTCCAACCCGGAAAGACACGACAAAACGCCACTG<br>GCAGCAGCCATTGGTAACTGAGAATTAGTGGATTTAGATATCGAGAGTCTTGAAGT<br>GGTGGCCTAACAGAGGCTACACTGAAAGGACAGTATTTGGTATCTGCGCTCCACTA<br>AAGCCAGTTACCAGGTTAAGCAGTTCCCCAACTGACTTAACCTTCGATCAAACCGC<br>CTCCCCAGGCGGTTTTTTCGTTTACAGAGCAGGAGATTACGACGATCGTAAAAGGA<br>TCTCAAGAAGATCCTTTACGGATTCCCGACACCATCACTCTAGATTTCAGTGCAAT<br>TTATCTCTTCAAATGTAGCACCTGAAGTCAGCCCCATACGATATAAGTTGTAATTCT<br>CATGTTAGTCATGCCCCGCGCCCACCGGAAGGAGCTGACTGGGTTGAAGGCTCTCA<br>AGGGCATCGGTCGAGATCCCGGTGCCTAATGAGTGAGCTAACTTACATTAATTGCG<br>TTGCGCTCACTGCCCGCTTTCCAGTCGGGAAACCTGTCGTGCCAGCTGCATTAATG<br>AATCGGCCAACGCGCGGGGAGAGGCGGTTTGCGTATTGGGCGCCAGGGTGGTTTT<br>TCTTTTCACCAGTGAGACGGGCAACAGCTGATTGCCCTTCACCGCCTGGCCCTGAG<br>AGAGTTGCAGCAAGCGGTCCACGCTGGTTTGCCCCAGCAGGCGAAAATCCTGTTTG<br>ATGGTGGTTAACGGCGGGATATAACATGAGCTGTCTTCGGTATCGTCGTATCCCAC<br>TACCGAGATATCCGCACCAACGCGCAGCCCGGACTCGGTAATGGCGCGCATTGCG<br>CCCAGCGCCATCTGATCGTTGGCAACCAGCATCGCAGTGGGAACGATGCCCTCATT<br>CAGCATTTGCATGGTTTGTTGAAAACCGGACATGGCACTCCAGTCGCCTTCCCGTT<br>CCGCTATCGGCTGAATTTGATTGCGAGTGAGATATTTATGCCAGCCAGCCAGACGC<br>AGACGCGCCGAGACAGAACTTAATGGGCCCGCTAACAGCGCGATTTGCTGGTGAC<br>CCAATGCGACCAGATGCTCCACGCCCAGTCGCGTACCGTCTTCATGGGAGAAAATA<br>ATACTGTTGATGGGTGTCTGGTCAGAGACATCAAGAAATAACGCCGGAACATTAG<br>TGCAGGCAGCTTCCACAGCAATGGCATCCTGGTCATCCAGCGGATAGTTAATGATC<br>AGCCCACTGACGCGTTGCGCGAGAAGATTGTGCACCGCCGCTTTACAGGCTTCGAC<br>GCCGCTTCGTTCTACCATCGACACCACCACGCTGGCACCCAGTTGATCGGCGCGAG<br>ATTTAATCGCCGCGACAATTTGCGACGGCGCGTGCAGGGCCAGACTGGAGGTGGC<br>AACGCCAATCAGCAACGACTGTTTGCCCGCCAGTTGTTGTGCCACGCGGTTGGGAA<br>TGTAATTCAGCTCCGCCATCGCCGCTTCCACTTTTTCCCGCGTTTTCGCAGAAACGT<br>GGCTGGCCTGGTTCACCACGCGGGAAACGGTCTGATAAGAGACACCGGCATACTC<br>TGCGACATCGTATAACGTTACTGGTTTCACATTCACCACCCTGAATTGACTCTCTTC<br>CGGGCGCTATCATGCCATACCGCGAAAGGTTTTGCGCCATTCGATGGTGTC |
| Trigger RNA<br><br>[pCDFDuet] | CGGGATCTCGACGCTCTCCCTTATGAGTGATAGCCGTTTGTCTGGTGTCTACGCCGC<br>GCATAAATGTGAGCGGATAACATTGACATTGTGAGCGGATAACAAGATACTGAGC<br>ACGCTCGATCACTAATCTGATCGAGACGAACATACCTACCTTCATTATCTTACTTGT<br>TAGCATAACCCCTTGGGGCCTCTAAACGGGTCTTGAGGGGTTTTTTGCTGAAACCT<br>CAGGCATTTGAGAAGCACACGGTCACACTGCTTCCGGTAGTCAATAAACCGGTAA<br>ACCAGCAATAGACATAAGCGGCTATTTAACGACCCTGCCCTGAACCGACGACCGG<br>GTCATCGTGGCCGGATCTTGCGGCCCCTCGGCTTGAACGAATTGTTAGACATTATT |

| | |
|---|---|
| (pLlacO-1 – Trigger RNA – T7term – SpecR – CloDF13 Origin – LacI) | TGCCGACTACCTTGGTGATCTCGCCTTTCACGTAGTGGACAAATTCTTCCAACTGAT<br>CTGCGCGCGAGGCCAAGCGATCTTCTTCTTGTCCAAGATAAGCCTGTCTAGCTTCA<br>AGTATGACGGGCTGATACTGGGCCGGCAGGCGCTCCATTGCCCAGTCGGCAGCGA<br>CATCCTTCGGCGCGATTTTGCCGGTTACTGCGCTGTACCAAATGCGGGACAACGTA<br>AGCACTACATTTCGCTCATCGCCAGCCCAGTCGGGCGGCGAGTTCCATAGCGTTAA<br>GGTTTCATTTAGCGCCTCAAATAGATCCTGTTCAGGAACCGGATCAAAGAGTTCCT<br>CCGCCGCTGGACCTACCAAGGCAACGCTATGTTCTCTTGCTTTTGTCAGCAAGATA<br>GCCAGATCAATGTCGATCGTGGCTGGCTCGAAGATACCTGCAAGAATGTCATTGCG<br>CTGCCATTCTCCAAATTGCAGTTCGCGCTTAGCTGGATAACGCCACGGAATGATGT<br>CGTCGTGCACAACAATGGTGACTTCTACAGCGCGGAGAATCTCGCTCTCTCCAGGG<br>GAAGCCGAAGTTTCCAAAAGGTCGTTGATCAAAGCTCGCCGCGTTGTTTCATCAAG<br>CCTTACGGTCACCGTAACCAGCAAATCAATATCACTGTGTGGCTTCAGGCCGCCAT<br>CCACTGCGGAGCCGTACAAATGTACGGCCAGCAACGTCGGTTCGAGATGGCGCTC<br>GATGACGCCAACTACCTCTGATAGTTGAGTCGATACTTCGGCGATCACCGCTTCCC<br>TCATACTCTTCCTTTTTCAATATTATTGAAGCATTTATCAGGGTTATTGTCTCATGA<br>GCGGATACATATTTGAATGTATTTAGAAAAATAAACAAATAGCTAGCTCACTCGGT<br>CGCTACGCTCCGGGCGTGAGACTGCGGCGGGCGCTGCGGACACATACAAAGTTAC<br>CCACAGATTCCGTGGATAAGCAGGGGACTAACATGTGAGGCAAAACAGCAGGGCC<br>GCGCCGGTGGCGTTTTTCCATAGGCTCCGCCCTCCTGCCAGAGTTCACATAAACAG<br>ACGCTTTTCCGGTGCATCTGTGGGAGCCGTGAGGCTCAACCATGAATCTGACAGTA<br>CGGGCGAAACCCGACAGGACTTAAAGATCCCCACCGTTTCCGGCGGGTCGCTCCCT<br>CTTGCGCTCTCCTGTTCCGACCCTGCCGTTTACCGGATACCTGTTCCGCCTTTCTCC<br>CTTACGGGAAGTGTGGCGCTTTCTCATAGCTCACACACTGGTATCTCGGCTCGGTG<br>TAGGTCGTTCGCTCCAAGCTGGGCTGTAAGCAAGAACTCCCCGTTCAGCCCGACTG<br>CTGCGCCTTATCCGGTAACTGTTCACTTGAGTCCAACCCGGAAAAGCACGGTAAAA<br>CGCCACTGGCAGCAGCCATTGGTAACTGGGAGTTCGCAGAGGATTTGTTTAGCTAA<br>ACACGCGGTTGCTCTTGAAGTGTGCGCCAAAGTCCGGCTACACTGGAAGGACAGA<br>TTTGGTTGCTGTGCTCTGCGAAAGCCAGTTACCACGGTTAAGCAGTTCCCCAACTG<br>ACTTAACCTTCGATCAAACCACCTCCCCAGGTGGTTTTTTCGTTTACAGGGCAAAA<br>GATTACGCGCAGAAAAAAAGGATCTCAAGAAGATCCTTTGATCTTTTCTACTGAAC<br>CGCTCTAGATTTCAGTGCAATTTATCTCTTCAAATGTAGCACCTGAAGTCAGCCCC<br>ATACGATATAAGTTGTAATTCTCATGTTAGTCATGCCCCGCGCCCACCGGAAGGAG<br>CTGACTGGGTTGAAGGCTCTCAAGGGCATCGGTCGAGATCCCGGTGCCTAATGAGT<br>GAGCTAACTTACATTAATTGCGTTGCGCTCACTGCCCGCTTTCCAGTCGGGAAACC<br>TGTCGTGCCAGCTGCATTAATGAATCGGCCAACGCGCGGGGAGAGGCGGTTTGCG<br>TATTGGGCGCCAGGGTGGTTTTTCTTTTCACCAGTGAGACGGGCAACAGCTGATTG<br>CCCTTCACCGCCTGGCCCTGAGAGAGTTGCAGCAAGCGGTCCACGCTGGTTTGCCC<br>CAGCAGGCGAAAATCCTGTTTGATGGTGGTTAACGGCGGGATATAACATGAGCTG<br>TCTTCGGTATCGTCGTATCCCACTACCGAGATATCCGCACCAACGCGCAGCCCGGA<br>CTCGGTAATGGCGCGCATTGCGCCCAGCGCCATCTGATCGTTGGCAACCAGCATCG<br>CAGTGGGAACGATGCCCTCATTCAGCATTTGCATGGTTTGTTGAAAACCGGACATG<br>GCACTCCAGTCGCCTTCCCGTTCCGCTATCGGCTGAATTTGATTGCGAGTGAGATA<br>TTTATGCCAGCCAGCCAGACGCAGACGCGCCGAGACAGAACTTAATGGGCCCGCT<br>AACAGCGCGATTTGCTGGTGACCCAATGCGACCAGATGCTCCACGCCCAGTCGCGT<br>ACCGTCTTCATGGGAGAAAATAATACTGTTGATGGGTGTCTGGTCAGAGACATCAA<br>GAAATAACGCCGGAACATTAGTGCAGGCAGCTTCCACAGCAATGGCATCCTGGTC<br>ATCCAGCGGATAGTTAATGATCAGCCCACTGACGCGTTGCGCGAGAAGATTGTGC<br>ACCGCCGCTTTACAGGCTTCGACGCCGCTTCGTTCTACCATCGACACCACCACGCT<br>GGCACCCAGTTGATCGGCGCGAGATTTAATCGCCGCGACAATTTGCGACGGCGCGT<br>GCAGGGCCAGACTGGAGGTGGCAACGCCAATCAGCAACGACTGTTTGCCCGCCAG<br>TTGTTGTGCCACGCGGTTGGGAATGTAATTCAGCTCCGCCATCGCCGCTTCCACTTT<br>TTCCCGCGTTTTCGCAGAAACGTGGCTGGCCTGGTTCACCACGCGGGAAACGGTCT<br>GATAAGAGACACCGGCATACTCTGCGACATCGTATAACGTTACTGGTTTCACATTC<br>ACCACCCTGAATTGACTCTCTTCCGGGCGCTATCATGCCATACCGCGAAAGGTTTT<br>GCGCCATTCGATGGTGTC |
| GFP positive<br><br>[pACYCDuet] | CTCCCTTATGCTAGTAAATTCGCGTTTCTACGGTAGTCCCTATCAGTGATAGAGATT<br>GACATCCCTATCAGTGATAGATATACTGAGCACACCTAAGGTAAATAAGGAGGAG<br>TAACATGAAAGAGACGAACCTGGCGGCAGCGCAAAAGATGCGTAAAGGAGAAGA<br>ACTTTTCACTGGAGTTGTCCCAATTCTTGTTGAATTAGATGGTGATGTTAATGGGCA<br>CAAATTTTCTGTCAGTGGAGAGGGTGAAGGTGATGCAACATACGGAAAACTTACC<br>CTTAAATTTATTTGCACTACTGGAAAACTACCTGTTCCGTGGCCAACACTTGTCACT<br>ACTTTCGGTTATGGTGTTCAATGCTTTGCGAGATACCCAGATCACATGAAACAGCA<br>TGACTTTTTCAAGAGTGCCATGCCCGAAGGTTACGTACAGGAAAGAACTATATTTT<br>TCAAAGATGACGGGAACTACAAGACACGTGCTGAAGTCAAGTTTGAAGGTGATAC<br>CCTTGTTAATAGAATCGAGTTAAAAGGTATTGATTTTAAAGAAGATGGAAACATTC |

| | |
|---|---|
| (pLtetO-1 – GFPmut3b - ssrA(ASV) – T7term - CmR – p15A origin – LacI) | TTGGACACAAATTGGAATACAACTATAACTCACACAATGTATACATCATGGCAGAC<br>AAACAAAAGAATGGAATCAAAGTTAACTTCAAAATTAGACACAACATTGAAGATG<br>GAAGCGTTCAACTAGCAGACCATTATCAACAAAATACTCCGATTGGCGATGGCCCT<br>GTCCTTTTACCAGACAACCATTACCTGTCCACACAATCTGCCCTTTCGAAAGATCCC<br>AACGAAAAGAGAGACCACATGGTCCTTCTTGAGTTTGTAACCGCTGCTGGGATTAC<br>ACATGGCATGGATGAACTATACAAAAGGCCTGCAGCAAACGACGAAAACTACGCT<br>GCATCAGTTTAATAATAATAAGATAAACCAGAGCGGCACGGCAAGCAGAGTATAC<br>GAGATTCGGTAGCCACCGCTGAGCAATAACTAGCATAACCCCTTGGGGCCTCTAAA<br>CGGGTCTTGAGGGGTTTTTTGGGCGAGCTACTCGTTTGCGTAGAAGTAGTTGGCAT<br>TTGAGAAGCACACGGTCACACTGCTTCCGGTAGTCAATAAACCGGTAAACCAGCA<br>ATAGACATAAGCGGCTATTTAACGACCCTGCCCTGAACCGACGACCGGGTCGAATT<br>TGCTTTCGAATTTCTGCCATTCATCCGCTTATTATCACTTATTCAGGCGTAGCAACC<br>AGGCGTTTAAGGGCACCAATAACTGCCTTAAAAAAATTACGCCCCGCCCTGCCACT<br>CATCGCAGTACTGTTGTAATTCATTAAGCATTCTGCCGACATGGAAGCCATCACAG<br>ACGGCATGATGAACCTGAATCGCCAGCGGCATCAGCACCTTGTCGCCTTGCGTATA<br>ATATTTGCCCATAGTGAAAACGGGGGCGAAGAAGTTGTCCATATTGGCCACGTTTA<br>AATCAAAACTGGTGAAACTCACCCAGGGATTGGCTGAGACGAAAAACATATTCTC<br>AATAAACCCTTTAGGGAAATAGGCCAGGTTTTCACCGTAACACGCCACATCTTGCG<br>AATATATGTGTAGAAACTGCCGGAAATCGTCGTGGTATTCACTCCAGAGCGATGAA<br>AACGTTTCAGTTTGCTCATGGAAAACGGTGTAACAAGGGTGAACACTATCCCATAT<br>CACCAGCTCACCGTCTTTCATTGCCATACGGAACTCCGGATGAGCATTCATCAGGC<br>GGGCAAGAATGTGAATAAAGGCCGGATAAAACTTGTGCTTATTTTTCTTTACGGTC<br>TTTAAAAAGGCCGTAATATCCAGCTGAACGGTCTGGTTATAGGTACATTGAGCAAC<br>TGACTGAAATGCCTCAAAATGTTCTTTACGATGCCATTGGGATATATCAACGGTGG<br>TATATCCAGTGATTTTTTTCTCCATTTTAGCTTCCTTAGCTCCTGAAAATCTCGATA<br>ACTCAAAAAATACGCCCGGTAGTGATCTTATTTCATTATGGTGAAAGTTGGAACCT<br>CTTACGTGCCGATCAACGTCTCATTTTCGCCAAAAGTTGGCCCAGGGCTTCCCGGT<br>ATCAACAGGGACACCAGGATTTATTTATTCTGCGAAGTGATCTTCCGTCACAGGTA<br>TTTATTCGGCGCAAAGTGCGTCGGGTGATGCTGCCAACTTACTGATTTAGTGTATG<br>ATGGTGTTTTTGAGGTGCTCCAGTGGCTTCTGTTTCTATCAGCTGTCCCTCCTGTTC<br>AGCTACTGACGGGGTGGTGCGTAACGGCAAAAGCACCGCCGGACATCAGCGCTAG<br>CGGAGTGTATACTGGCTTACTATGTTGGCACTGATGAGGGTGTCAGTGAAGTGCTT<br>CATGTGGCAGGAGAAAAAAGGCTGCACCGGTGCGTCAGCAGAATATGTGATACAG<br>GATATATTCCGCTTCCTCGCTCACTGACTCGCTACGCTCGGTCGTTCGACTGCGGCG<br>AGCGGAAATGGCTTACGAACGGGGCGGAGATTTCCTGGAAGATGCCAGGAAGATA<br>CTTAACAGGGAAGTGAGAGGGCCGCGGCAAAGCCGTTTTTCCATAGGCTCCGCCC<br>CCCTGACAAGCATCACGAAATCTGACGCTCAAATCAGTGGTGGCGAAACCCGACA<br>GGACTATAAAGATACCAGGCGTTTCCCCTGGCGGCTCCCTCGTGCGCTCTCCTGTT<br>CCTGCCTTTCGGTTTACCGGTGTCATTCCGCTGTTATGGCCGCGTTTGTCTCATTCC<br>ACGCCTGACACTCAGTTCCGGGTAGGCAGTTCGCTCCAAGCTGGACTGTATGCACG<br>AACCCCCCGTTCAGTCCGACCGCTGCGCCTTATCCGGTAACTATCGTCTTGAGTCC<br>AACCCGGAAAGACATGCAAAAGCACCACTGGCAGCAGCCACTGGTAATTGATTTA<br>GAGGAGTTAGTCTTGAAGTCATGCGCCGGTTAAGGCTAAACTGAAAGGACAAGTT<br>TTGGTGACTGCGCTCCTCCAAGCCAGTTACCTCGGTTCAAAGAGTTGGTAGCTCAG<br>AGAACCTTCGAAAAACCGCCCTGCAAGGCGGTTTTTTCGTTTTCAGAGCAAGAGAT<br>TACGCGCAGACCAAAACGATCTCAAGAAGATCATCTTATTAATCAGATAAAATATT<br>TCTAGATTTCAGTGCAATTTATCTCTTCAAATGTAGCACCTGAAGTCAGCCCCATAC<br>GATATAAGTTGTAATTCTCATGTTAGTCATGCCCCGCGCCCACCGGAAGGAGCTGA<br>CTGGGTTGAAGGCTCTCAAGGGCATCGGTCGAGATCCCGGTGCCTAATGAGTGAG<br>CTAACTTACATTAATTGCGTTGCGCTCACTGCCCGCTTTCCAGTCGGGAAACCTGTC<br>GTGCCAGCTGCATTAATGAATCGGCCAACGCGCGGGGAGAGGCGGTTTGCGTATT<br>GGGCGCCAGGGTGGTTTTTCTTTTCACCAGTGAGACGGGCAACAGCTGATTGCCCT<br>TCACCGCCTGGCCCTGAGAGAGTTGCAGCAAGCGGTCCACGCTGGTTTGCCCCAGC<br>AGGCGAAAATCCTGTTTGATGGTGGTTAACGGCGGGATATAACATGAGCTGTCTTC<br>GGTATCGTCGTATCCCACTACCGAGATATCCGCACCAACGCGCAGCCCGGACTCGG<br>TAATGGCGCGCATTGCGCCCAGCGCCATCTGATCGTTGGCAACCAGCATCGCAGTG<br>GGAACGATGCCCTCATTCAGCATTTGCATGGTTTGTTGAAAACCGGACATGGCACT<br>CCAGTCGCCTTCCCGTTCCGCTATCGGCTGAATTTGATTGCGAGTGAGATATTTATG<br>CCAGCCAGCCAGACGCAGACGCGCCGAGACAGAACTTAATGGGCCCGCTAACAGC<br>GCGATTTGCTGGTGACCCAATGCGACCAGATGCTCCACGCCCAGTCGCGTACCGTC<br>TTCATGGGAGAAAATAATACTGTTGATGGGTGTCTGGTCAGAGACATCAAGAAAT<br>AACGCCGGAACATTAGTGCAGGCAGCTTCCACAGCAATGGCATCCTGGTCATCCA<br>GCGGATAGTTAATGATCAGCCCACTGACGCGTTGCGCGAGAAGATTGTGCACCGC<br>CGCTTTACAGGCTTCGACGCCGCTTCGTTCTACCATCGACACCACCACGCTGGCAC<br>CCAGTTGATCGGCGCGAGATTTAATCGCCGCGACAATTTGCGACGGCGCGTGCAG |

| | |
|---|---|
| | GGCCAGACTGGAGGTGGCAACGCCAATCAGCAACGACTGTTTGCCCGCCAGTTGTT<br>GTGCCACGCGGTTGGGAATGTAATTCAGCTCCGCCATCGCCGCTTCCACTTTTTCCC<br>GCGTTTTCGCAGAAACGTGGCTGGCCTGGTTCACCACGCGGGAAACGGTCTGATAA<br>GAGACACCGGCATACTCTGCGACATCGTATAACGTTACTGGTTTCACATTCACCAC<br>CCTGAATTGACTCTCTTCCGGGCGCTATCATGCCATACCGCGAAAGGTTTTGCGCC<br>ATTCGATGGTGTC |

**Supplementary Table S4.** Synthetic bacterial small RNA sequences used in the study. The structure of sRNA was referenced from a prior study that developed a reusable high-performance sRNA pool. All the sRNAs are cloned into the pET15b plasmid, with an architecture with [pT7 – target variable region (15 nt) – Hfq scaffold (13) – rho independent terminator – T7term]. The variable region and Hfq scaffold are specified with underline and blue lane, respectively.

| Name | Sequence | Experiment used | Reference |
|---|---|---|---|
| sRNA decoy | ATTTGTAGAAATATTTTATTC<br>GCCCCCGGAAGATCATTCCGGGGGCTTTTTTATT | **Figure 2, 3, 4, 5, 6, 7, and 8**<br><br>**Supplementary Figure S3, S4, S5, S6, S7, S8, S9, S10, S11, S12, S13, S14, S15, S16, S18, S19, S22 and S25** | This study |
| sRNA variant 1 | GTTCTTGTCTTGTTCATTTGTAGAAATATTTTATTC<br>GCCCCCGGAAGATCATTCCGGGGGCTTTTTTATT | **Figure 2c, 2d, 2e, 4, 5, 6i-p, 7 and 8**<br><br>**Supplementary Figure S1, S3, S4, S5, S10, S13, S14, S15, S17, S18, S19, S21, S22, S23, S24 and S25** | This study |
| sRNA variant 2 | GTCTGTTTCTGTTTCATTTGTAGAAATATTTTATTC<br>GCCCCCGGAAGATCATTCCGGGGGCTTTTTTATT | **Figure 2d and 3g-k** | This study |

| | | | |
|---|---|---|---|
| | | Supplementary Figure S3, S7 and S9 | |
| sRNA variant 3 | GTCTTTGTCTTGTTCATTTGTAGAAATATTTTATTC GCCCCCGGAAGATCATTCCGGGGGCTTTTTTATT | Figure 2d and 3g-k<br><br>Supplementary Figure S3, S7 and S9 | This study |
| sRNA variant 4 | GTCAAAGTTTGTTATATTTGTAGAAATATTTTATTC GCCCCCGGAAGATCATTCCGGGGGCTTTTTTATT | Supplementary Figure S8 | (13) |
| sRNA orthogonal 1 (sRNA O1) | CTTCCATTTACTTTCATTTGTAGAAATATTTTATTC GCCCCCGGAAGATCATTCCGGGGGCTTTTTTATT | Figure 2f<br><br>Supplementary Figure S6 and S9 | This study |
| sRNA orthogonal 2 (sRNA O2) | GACTCTTACTATTTCATTTGTAGAAATATTTTATTC GCCCCCGGAAGATCATTCCGGGGGCTTTTTTATT | Figure 2f<br><br>Supplementary Figure S6 | This study |
| sRNA orthogonal 3 (sRNA O3) | GTTCCTGTTCTATTCATTTGTAGAAATATTTTATTC GCCCCCGGAAGATCATTCCGGGGGCTTTTTTATT | Figure 2f<br><br>Supplementary Figure S6 | This study |
| sRNA orthogonal 4 (sRNA O4) | ATTGTATTTGTCTTTATTTGTAGAAATATTTTATTC GCCCCCGGAAGATCATTCCGGGGGCTTTTTTATT | Figure 2f<br><br>Supplementary Figure S6 | This study |
| sRNA orthogonal 5 (sRNA O5) | CTTTACACTTTCACTATTTGTAGAAATATTTTATTC GCCCCCGGAAGATCATTCCGGGGGCTTTTTTATT | Figure 2f<br><br>Supplementary Figure S6 and S9 | This study |
| sRNA ENYC3 | AGCAGGGTGCTGCCAATTTGTAGAAATATTTTATTC GCCCCCGGAAGATCATTCCGGGGGCTTTTTTATT | Figure 6a-c<br><br>Supplementary Figure S11 | This study |
| sRNA 4U | AAATCACTATTTAAAATTTGTAGAAATATTTTATTC GCCCCCGGAAGATCATTCCGGGGGCTTTTTTATT | Figure 6d-f<br><br>Supplementary Figure S12 | This study |
| sRNA START_theophylline | GTTCTATTGTCTACTATTTGTAGAAATATTTTATTC GCCCCCGGAAGATCATTCCGGGGGCTTTTTTATT | Figure 6i-l<br><br>Supplementary Figure S13 | This study |

| | | | |
|---|---|---|---|
| sRNA START_MS2 | GTTCTAAAGTTTAACATTTGTAGAAATATTTTATTC GCCCCCGGAAGATCATTCCGGGGGCTTTTTTATT | **Figure 6m-p**<br><br>**Supplementary Figure S14** | This study |

**Supplementary Table S5.** Sequence of the toehold switches used in **Figure 2**. The detailed architecture from the promoter to the terminator is specified, along with the exact sequences corresponding to the toehold switches. Sequences referenced from previous study are highlighted with a pink lane (5). The sRNA binding region and RBS are underlined or bolded, respectively. All these toehold switches are cloned into the pCOLADuet plasmid.

| Name | (Architecture) Sequence | Experiment used |
|---|---|---|
| Toehold switch 1 sRNA variant 1 | (pJ23110 – lacO – **riboregulator** – GFPmut3b – ssrA(ASV) – T7term)<br>AGTAAGATAATGAAGGTAGGTATGTTAAACTTTAGAACAAGA CAAGAAC**AGAGGAGA**TAAAGATGAACATACCTACGAACCTG GCGGCAGCGCAAAAG | **Figure 2b-e**<br><br>**Supplementary Figure S3C, D, S4, and S5** |
| Toehold switch 1 sRNA variant 2 | (pJ23110 – lacO – **riboregulator** – GFPmut3b – ssrA(ASV) – T7term)<br>AGTAAGATAATGAAGGTAGGTATGTTAAACTTTAGAAACAGA AACAGAC**AGAGGAGA**TAAAGATGAACATACCTACGAACCTG GCGGCAGCGCAAAAG | **Figure 2d**<br><br>**Supplementary Figure S3C, D** |
| Toehold switch 1 sRNA variant 3 | (pJ23110 – lacO – **riboregulator** – GFPmut3b – ssrA(ASV) – T7term)<br>AGTAAGATAATGAAGGTAGGTATGTTAAACTTTAGAACAAGA CAAAGAC**AGAGGAGA**TAAAGATGAACATACCTACGAACCTG GCGGCAGCGCAAAAG | **Figure 2d**<br><br>**Supplementary Figure S3C, D** |
| Toehold switch 1 sRNA variant 2 with TagBFP | (pJ23110 – lacO – **riboregulator** – TagBFP – ssrA(ASV) – T7term)<br>AGTAAGATAATGAAGGTAGGTATGTTAAACTTTAGAAACAGA AACAGAC**AGAGGAGA**TAAAGATGAACATACCTACGAACCTG GCGGCAGCGCAAAAG | **Figure 3g-k**<br><br>**Supplementary Figure S9** |
| Orthogonal switch 1 (THS-O1) | (pJ23110 – lacO – **riboregulator** – GFPmut3b – ssrA(ASV) – T7term)<br>AGTAAGATAATGAAGGTAGGTATGTTAAACTTTAGAAAGTAA ATGGAAG**AGAGGAGA**TAAAGATGAACATACCTACGAACCTG GCGGCAGCGCAAAAG | **Figure 2f**<br><br>**Supplementary Figure S6 and S9** |
| Orthogonal switch 2 (THS-O2) | (pJ23110 – lacO – **riboregulator** – GFPmut3b – ssrA(ASV) – T7term)<br>AGTAAGATAATGAAGGTAGGTATGTTAAACTTTAGAAATAGTA AGAGTC**AGAGGAGA**TAAAGATGAACATACCTACGAACCTGG CGGCAGCGCAAAAG | **Figure 2f**<br><br>**Supplementary Figure S6** |
| Orthogonal switch 3 | (pJ23110 – lacO – **riboregulator** – GFPmut3b – | **Figure 2f** |

| | | |
|---|---|---|
| (THS-O3) | ssrA(ASV) – T7term) AGTAAGATAATGAAGGTAGGTATGTTAAACTTTAGAATAGAACAGGAAC**AGAGGAGA**TAAAGATGAACATACCTACGAACCTGGCGGCAGCGCAAAAG | **Supplementary Figure S6** |
| Orthogonal switch 4 (THS-O4) | (pJ23110 – lacO – **riboregulator** – GFPmut3b – ssrA(ASV) – T7term) AGTAAGATAATGAAGGTAGGTATGTTAAACTTTAAAAGACAAATACAAT**AGAGGAGA**TAAAGATGAACATACCTACGAACCTGGCGGCAGCGCAAAAG | **Figure 2f**<br><br>**Supplementary Figure S6** |
| Orthogonal switch 5 (THS-O5) | (pJ23110 – lacO – **riboregulator** – GFPmut3b – ssrA(ASV) – T7term) AGTAAGATAATGAAGGTAGGTATGTTAAACTTTAAGTGAAAGTGTAAAG**AGAGGAGA**TAAAGATGAACATACCTACGAACCTGGCGGCAGCGCAAAAG | **Figure 2f**<br><br>**Supplementary Figure S6 and S9** |
| Constitutive toehold switch 1 sRNA variant 1 | (pJ23110 – **riboregulator** – GFPmut3b – ssrA(ASV) – T7term) AGTAAGATAATGAAGGTAGGTATGTTAAACTTTAGAACAAGACAAGAAC**AGAGGAGA**TAAAGATGAACATACCTACGAACCTGGCGGCAGCGCAAAAG | **Figure 4** |

**Supplementary Table S6.** Sequence variations in the trigger binding domain across toehold switch variants in **Supplementary Figure S3**. Sequence changes compared to the toehold switch in **Figure 2** are highlighted with a blue lane. Trigger binding domains are adopted from the previous study (5). The sRNA binding region and RBS are underlined or **bolded**, respectively. All these toehold switches are cloned into the pCOLADuet plasmid.

| Name | (Architecture) Sequence | Experiment used |
|---|---|---|
| Toehold switch 2 sRNA variant 1 | Switch RNA, pCOLADuet (pJ23110 – lacO – **riboregulator** – GFPmut3b – ssrA(ASV) – T7term) ATTGAATATGATAGAAGTTTAGTAGTAGACAATAGAACAAGACAAGAAC**AGAGGAGA**TATTGATGACTACTAAACTAAACCTGGCGGCAGCGCAAAAG<br><br>Trigger RNA, pCDFDuet (pLlacO – **Trigger RNA** – T7term) GATACACATAGAATCATGTGTATAACACTACTAAACTTCTATCATATTCAATCAC | **Supplementary Figure S3E, F** |
| Toehold switch 2 sRNA variant 2 | Switch RNA, pCOLADuet (pLlacO-1 – **riboregulator** – GFPmut3b – ssrA(ASV) – T7term) ATTGAATATGATAGAAGTTTAGTAGTAGACAATAGAAACAGAAACAGAC**AGAGGAGA**TATTGATGACTACTAAACTAAACCTGGCGGCAGCGCAAAAG<br><br>Trigger RNA, pCDFDuet (pLlacO – **Trigger RNA** – T7term) GATACACATAGAATCATGTGTATAACACTACTAAACTTCTATCATATTCAATCAC | **Supplementary Figure S3E, F** |
| Toehold switch 2 | Switch RNA, pCOLADuet | **Supplementary** |

| | | |
|---|---|---|
| sRNA variant 3 | (pLlacO-1 – **riboregulator** – GFPmut3b – ssrA(ASV) – T7term)<br>ATTGAATATGATAGAAGTTTAGTAGTAGACAATAGAACAAGACAAAGAC**AGAGGAGA**TATTGATGACTACTAAACTAAACCTGGCGGCAGCGCAAAAG | **Figure S3E, F** |
| | Trigger RNA, pCDFDuet<br>(pLlacO – **Trigger RNA** – T7term)<br>GATACACATAGAATCATGTGTATAACACTACTAAACTTCTATCATATTCAATCAC | |
| Toehold switch 3 sRNA variant 1 | Switch RNA, pCOLADuet<br>(pLlacO-1 – **riboregulator** – GFPmut3b – ssrA(ASV) – T7term)<br>ACTGATTTGAATACACTGCTTCGTTCAAGATTCAGAACAAGACAAGAAC**AGAGGAGA**TGAATATGGAACGAAGCAGAAACCTGGCGGCAGCGCAAAAG | **Supplementary Figure S3G, H** |
| | Trigger RNA, pCDFDuet<br>(pLlacO – **Trigger RNA** – T7term)<br>AGTTGCGGCACGGACCGCAACTATAGAACGAAGCAGTGTATTCAAATCAGTTAG | |
| Toehold switch 3 sRNA variant 2 | Switch RNA, pCOLADuet<br>(pLlacO-1 – **riboregulator** – GFPmut3b – ssrA(ASV) – T7term)<br>ACTGATTTGAATACACTGCTTCGTTCAAGATTCAGAAACAGAAACAGAC**AGAGGAGA**TGAATATGGAACGAAGCAGAAACCTGGCGGCAGCGCAAAAG | **Supplementary Figure S3G, H** |
| | Trigger RNA, pCDFDuet<br>(pLlacO – **Trigger RNA** – T7term)<br>AGTTGCGGCACGGACCGCAACTATAGAACGAAGCAGTGTATTCAAATCAGTTAG | |
| Toehold switch 3 sRNA variant 3 | Switch RNA, pCOLADuet<br>(pLlacO-1 – **riboregulator** – GFPmut3b – ssrA(ASV) – T7term)<br>ACTGATTTGAATACACTGCTTCGTTCAAGATTCAGAACAAGACAAAGAC**AGAGGAGA**TGAATATGGAACGAAGCAGAAACCTGGCGGCAGCGCAAAAG | **Supplementary Figure S3G, H** |
| | Trigger RNA, pCDFDuet<br>(pLlacO – **Trigger RNA** – T7term)<br>AGTTGCGGCACGGACCGCAACTATAGAACGAAGCAGTGTATTCAAATCAGTTAG | |

**Supplementary Table S7.** Sequences for the synthetic riboregulators in **Supplementary Figure S7, and S8**. The detailed architecture from the promoter to the terminator is specified, along with the exact sequences corresponding to the riboregulators. Sequences referenced from previous studies are highlighted with a blue lane (11,12) or pink lane (5). The sRNA binding region and RBS are underlined or **bolded**, respectively. All these riboregulators are cloned into the pCOLADuet plasmid.

| Name<br>(Cognate sRNA) | (Architecture)<br>Sequence | Experiment used |
|---|---|---|
| STAR 1_1<br>(sRNA variant 2) | Switch RNA, pCOLADuet<br>(pLlacO-1 – **riboregulator** – GFPmut3b – ssrA(ASV) – T7term)<br>AGTTTTTACAGTGAATTGTTTTAATTAGTTGTATAAATGTTGGAGCAGC<br>GGGGAATGTATACAGTTCATGTATATATTCCCCGCTTTTTTTTTAACCTG<br>GCGGCAGCGCAAAAGGAAACAGAAACAGAC**AGAGGAGA**CAGAG | **Supplementary Figure S7C, D** |
| | Trigger RNA, pCDFDuet<br>(pLlacO – **Trigger RNA** – T7term)<br>TGAACTGTATACATTCCCCGCTGCTCCAACATTTATACAACTAATTAAA<br>ACAATTCACTGTAAAAACT | |
| STAR 1_2<br>(sRNA variant 3) | Switch RNA, pCOLADuet<br>(pLlacO-1 – **riboregulator** – GFPmut3b – ssrA(ASV) – T7term)<br>AGTTTTTACAGTGAATTGTTTTAATTAGTTGTATAAATGTTGGAGCAGC<br>GGGGAATGTATACAGTTCATGTATATATTCCCCGCTTTTTTTTTAACCTG<br>GCGGCAGCGCAAAAGGAACAAGACAAAGAC**AGAGGAGA**CAGAG | **Supplementary Figure S7C, D** |
| | Trigger RNA, pCDFDuet<br>(pLlacO – **Trigger RNA** – T7term)<br>TGAACTGTATACATTCCCCGCTGCTCCAACATTTATACAACTAATTAAA<br>ACAATTCACTGTAAAAACT | |
| STAR 2_1<br>sRNA variant 2 | Switch RNA, pCOLADuet<br>(pLlacO-1 – **riboregulator** – GFPmut3b – ssrA(ASV) – T7term)<br>CCATCTTACCTTTGCATCTCTATCGTTCTCATCTCATCCTGCGGGGAAT<br>GTATACAGTTCATGTATATATTCCCCGCTTTTTTTTTAACCTGGCGGCAG<br>CGCAAAAGGAAACAGAAACAGAC**AGAGGAGA**CAGAG | **Supplementary Figure S7E, F** |
| | Trigger RNA, pCDFDuet<br>(pLlacO – **Trigger RNA** – T7term)<br>TGAACTGTATACATTCCCCGCAGGATGAGATGAGAACGATAGAGATGC<br>AAAGGTAAGATGG | |
| STAR 2_2<br>(sRNA variant 3) | Switch RNA, pCOLADuet<br>(pLlacO-1 – **riboregulator** – GFPmut3b – ssrA(ASV) – T7term)<br>CCATCTTACCTTTGCATCTCTATCGTTCTCATCTCATCCTGCGGGGAAT<br>GTATACAGTTCATGTATATATTCCCCGCTTTTTTTTTAACCTGGCGGCAG<br>CGCAAAAGGAACAAGACAAAGAC**AGAGGAGA**CAGAG | **Supplementary Figure S7E, F** |
| | Trigger RNA, pCDFDuet<br>(pLlacO – **Trigger RNA** – T7term)<br>TGAACTGTATACATTCCCCGCAGGATGAGATGAGAACGATAGAGATGC<br>AAAGGTAAGATGG | |
| | Trigger RNA, pCDFDuet<br>(pLlacO-1 – **Trigger RNA** – T7term)<br>CCCGTGGTGTTGTCTTACACCACGGGCAAACACATCACACACCTCCAT<br>TCACCTCCATTCTTTCTATTTTCTCTACT | |

| | | |
|---|---|---|
| 3WJ 1<br>(sRNA variant 4) | Switch RNA, pCOLADuet<br>(pJ23110 – lacO – **riboregulator** – GFPmut3b – ssrA(ASV) – T7term)<br>ATGAATGATATACACTTGTTATAGTTATGATAACAAACTTTGAC**AGAGGAGA**CATAACATGAACAAGCACGAATTGACTACACTAAACCTGGCGGCAGCGCAAAAG | **Supplementary Figure S8C, D** |
| | Trigger RNA, pCDFDuet<br>(pLlacO-1 – **Trigger RNA** – T7term)<br>GGGACGAATTGATTTGTCAATTCGTGCGTGTATATCATTCATCATACC | |
| 3WJ 2<br>(sRNA variant 4) | Switch RNA, pCOLADuet<br>(pJ23110 – lacO – **riboregulator** – GFPmut3b – ssrA(ASV) – T7term)<br>ATGAATGATATACACATGTAAACCTATTGATAACAAACTTTGAC**AGAGGAGA**CAATAGATGTACATGCACGAATTGACTACACTAAACCTGGCGGCAGCGCAAAAG | **Supplementary Figure S8E, F** |
| | Trigger RNA, pCDFDuet<br>(pLlacO-1 – **Trigger RNA** – T7term)<br>GGGACGAATTGATTTGTCAATTCGTGCGTGTATATCATTCATCATACC | |
| 3WJ 3<br>(sRNA variant 4) | Switch RNA, pCOLADuet<br>(pJ23110 – lacO – **riboregulator** – GFPmut3b – ssrA(ASV) – T7term)<br>ATGAATGATATACACTTGATAAGCTTATGATAACAAACTTTGAC**AGAGGAGA**CATAAGATGATCAAGCACGAATTGACTACACTAAACCTGGCGGCAGCGCAAAAG | **Supplementary Figure S8G, H** |
| | Trigger RNA, pCDFDuet<br>(pLlacO-1 – **Trigger RNA** – T7term)<br>GGGACGAATTGATTTGTCAATTCGTGCGTGTATATCATTCATCATACC | |

**Supplementary Table S8.** Summary of experimental conditions for leakage reduction and performance enhancement of riboregulators. Single colonies were grown overnight (~16 h) in 96-deep well plates with shaking at 800 rpm. For the overnight cell culture, the *Salmonella* 4U RNA thermometer experiments were conducted at 30°C, while all other experiments were conducted at 37°C. Overnight cultures were diluted 1:100 in the fresh LB media with appropriate antibiotics and returned to an orbital shaker. After 80 min, cell cultures were induced as following conditions. All GFP fluorescence measurements was conducted with *E. coli* BL21-AI™, and the number of biological replicates was three. *E. coli* BL21-AI™ has a chromosomally integrated T7 RNA polymerase under the control of the arabinose-inducible promoter, pBAD.

| Name (Experiment) | System composition (plasmid, promoter) | Induction condition (after 80min incubation from 1:100 dilution) | Induction time |
|---|---|---|---|
| Toehold switch | Switch RNA (pCOLADuet, pJ23110) | IPTG | 4h 30m |

| | | | |
|---|---|---|---|
| (**Figure 4**) | Trigger RNA (pCDFDuet, pLlacO-1) | 0, 0.05, 0.1, 0.25, 0.5, 1.0, or 2.0 mM<br><br>Arabinose (w/w)<br>0, 0.0002, 0.002, 0.02, or 0.2 % | |
| | sRNA (pET15b, pT7) | | |
| Cis-acting Theophylline riboswitch (**Figure 6b, c**) | ENYC3 theophylline riboswitch (pCOLADuet, pLlacO-1) | Theophylline<br>0 or 5 mM<br><br>Arabinose (w/w)<br>0, 0.0002, 0.0011, 0.002, 0.011, 0.02, 0.11, or 0.2 %<br><br>IPTG 0.1 mM | 3h 30m |
| | sRNA (pET15b, pT7) | | |
| *Salmonella* 4U RNA thermometer (**Figure 6e, f**) | 4U riboswitch (pCOLADuet, pLlacO-1) | Temperature<br>30°C or 37°C<br><br>Arabinose (w/w)<br>0, 0.0002, 0.0011, 0.002, 0.011, 0.02, 0.11, or 0.2 %<br><br>IPTG 0.1 mM | 3h 30m |
| | sRNA (pET15b, pT7) | | |
| Theophylline responsive START system (**Figure 6k, l**) | Switch RNA (pCOLADuet, pLlacO-1) | Theophylline<br>0 or 5 mM<br><br>Arabinose (w/w)<br>0, 0.0002, 0.0011, 0.002, 0.011, 0.02, 0.11, or 0.2 %<br><br>IPTG 0.1 mM | 4h 30m |
| | Trigger RNA (pCDFDuet, pLlacO-1) | | |
| | sRNA (pET15b, pT7) | | |
| MS2 responsive START system (**Figure 6o, p**) | Switch RNA (pCOLADuet, pLlacO-1) | IPTG 0.1 mM<br><br>Arabinose (w/w)<br>0, 0.0002, 0.0011, 0.002, 0.011, 0.02, 0.11, or 0.2 % | 4h 30m |
| | Trigger RNA (pCDFDuet, pLlacO-1) | | |
| | MS2 expression system or non-coding RNA expression system (pACYC, pLlacO-1) | | |
| | sRNA (pET15b, pT7) | | |

**Supplementary Table S9.** Sequence of the riboregulators used in **Figure 6** and **Supplementary Figure S11, S12, S13, and S14**. The detailed architecture from the promoter to the terminator is specified, along with the exact sequences corresponding to the riboregulator. The riboregulators listed here have not undergone any specific modifications from their reported sequences. The sRNA binding region is underlined, and the RBS is bolded. Aptamer sequences, when known, are indicated in blue. All these riboswitches are cloned into the pCOLADuet plasmid.

| Name | (Architecture)<br>Sequence | Experiment used | Reference |
|---|---|---|---|
| Cis-acting theophylline riboswitch (ENYC3) | (pLlacO-1 – **riboregulator** – GFPmut3b – ssrA(ASV) – T7term)<br>GGTGATACCAGCATCGTCTTGATGCCCTTGGCAGCACCCTGCT**AAGGAGG**TAACAACAAG | **Figure 6b, c**<br><br>**Supplementary Figure S11** | (6) |
| *Salmonella* 4U RNA thermometer | (pLlacO-1 – **riboregulator** – GFPmut3b – ssrA(ASV) – T7term)<br>AGCGTTGAACTTTTAAATAGTGATTT**AGGA**GGTTAATGATGGCAGAATTC | **Figure 6e, f**<br><br>**Supplementary Figure S12** | (7) |
| Trans-acting theophylline riboswitch (START system) | i) Switch (pLlacO-1 – **switch** – GFPmut3b – ssrA(ASV) – T7term)<br>ATTGAATATGATAGAAGTTTAGTAGTAGACAATAGAAC**AGAGGAGA**TATTGATGACTACTAAACTA | **Figure 6k, l**<br><br>**Supplementary Figure S13** | (8) |
| | ii) Trigger (pLlacO-1 – **Trigger with a theophylline aptamer** – T7term)<br>GATACACATAGAATCATGTGTATAACTACTAAACTTCAACTATGATACCAGCATCGTCTTGATGCCCTTGGCAGCATAAACTATCATATTCAC | | |
| Trans-acting MS2 riboswitch (START system) | i) Switch (pLlacO-1 – **switch** – GFPmut3b – ssrA(ASV) – T7term)<br>AGTAAGATAATGAAGGTAGGTATGTTAAACTTTAGAAC**AGAGGAGA**TAAAGATGAACATACCTACG | **Figure 6o, p**<br><br>**Supplementary Figure S14** | |
| | ii) Trigger (pLlacO-1 – **Trigger with a MS2 aptamer** – T7term)<br>GCTCGATCACTAATCTGATCGAGACGAACATACCTACCTACAAACATGAGGACCACCCATGTTAACTCATTATCTTACTTGT | | |

**Supplementary Table S10.** Sequence of the MS2 expression system in **Supplementary Figure S14**.

| Name (plasmid) | (Architecture)<br>Sequence | Experiment used | Reference |
|---|---|---|---|
| MS2 expression system (pACYCDuet) | (pLlacO-1 – 8X His tag – MBP – linker - MS2 coat protein – T7term)<br>ATAAATGTGAGCGGATAACATTGACATTGTGAGCGGATAACA<br>AGATACTGAGCACAAGAATTCAAAAGATCTAAAGAGGAGAAA<br>GGATCTATGCATCACCATCACCATCACCATCACAAAATCGAAGA<br>AGGTAAACTGGTAATCTGGATTAACGGCGATAAAGGCTATAACG<br>GTCTCGCTGAAGTCGGTAAGAAATTCGAGAAAGATACCGGAAT<br>TAAAGTCACCGTTGAGCATCCGGATAAACTGGAAGAGAAATTC<br>CCACAGGTTGCGGCAACTGGCGATGGCCCTGACATTATCTTCTG<br>GGCACACGACCGCTTTGGTGGCTACGCTCAATCTGGCCTGTTGG<br>CTGAAATCACCCCGGACAAAGCGTTCCAGGACAAGCTGTATCC<br>GTTTACCTGGGATGCCGTACGTTACAACGGCAAGCTGATTGCTT<br>ACCCGATCGCTGTTGAAGCGTTATCGCTGATTTATAACAAAGAT<br>CTGCTGCCGAACCCGCCAAAAACCTGGGAAGAGATCCCGGCGC<br>TGGATAAAGAACTGAAAGCGAAAGGTAAGAGCGCGCTGATGTT<br>CAACCTGCAAGAACCGTACTTCACCTGGCCGCTGATTGCTGCTG<br>ACGGGGGTTATGCGTTCAAGTATGAAAACGGCAAGTACGACAT<br>TAAAGACGTGGGCGTGGATAACGCTGGCGCGAAAGCGGGTCTG<br>ACCTTCCTGGTTGACCTGATTAAAAACAAACACATGAATGCAG<br>ACACCGATTACTCCATCGCAGAAGCTGCCTTTAATAAAGGCGAA<br>ACAGCGATGACCATCAACGGCCCGTGGGCATGGTCCAACATCG<br>ACACCAGCAAAGTGAATTATGGTGTAACGGTACTGCCGACCTTC<br>AAGGGTCAACCATCCAAACCGTTCGTTGGCGTGCTGAGCGCAG<br>GTATTAACGCCGCCAGTCCGAACAAAGAGCTGGCGAAAGAGTT<br>CCTCGAAAACTATCTGCTGACTGATGAAGGTCTGGAAGCGGTT<br>AATAAAGACAAACCGCTGGGTGCCGTAGCGCTGAAGTCTTACG<br>AGGAAGAGTTGGCGAAAGATCCACGTATTGCCGCCACCATGGA<br>AAACGCCCAGAAAGGTGAAATCATGCCGAACATCCCGCAGATG<br>TCCGCTTTCTGGTATGCCGTGCGTACTGCGGTGATCAACGCCGC<br>CAGCGGTCGTCAGACTGTCGATGAAGCCCTGAAAGACGCGCAG<br>ACTAATTCGAGCTCGGTACCCGGCCGGGGATCCATCGAGGGTAG<br>GGCTTCTAACTTTACTCAGTTCGTTCTCGTCGACAATGGCGGAA<br>CTGGCGACGTGACTGTCGCCCCAAGCAACTTCGCTAACGGGAT<br>CGCTGAATGGATCAGCTCTAACTCGCGTTCACAGGCTTACAAAG<br>TAACCTGTAGCGTTCGTCAGAGCTCTGCGCAGAATCGCAAATAC<br>ACCATCAAAGTCGAGGTGCCTAAAGGCGCCTGGCGTTCGTACT<br>TAAATATGGAACTAACCATTCCAATTTTCGCCACGAATTCCGACT<br>GCGAGCTTATTGTTAAGGCAATGCAAGGTCTCCTAAAAGATGGA<br>AACCCGATTCCCTCAGCAATCGCAGCAAACTCCGGCATCTACTA<br>ATAGCCACCGCTGAGCATAACTAGCATAACCCCTTGGGGCCTCT<br>AAACGGGTCTTGAGGGGTTTTTTG | **Supplementary Figure S14** | (8) |

**Supplementary Table S11.** Sequences of kill switch system in Figure **7** and **8**, and **Supplementary Figure S15, S16, S17, S18, S19, S20, S22, S23 and S24**. For the toehold switch, the RBS and sRNA binding site are indicated in **bold** and underline, respectively. The Holin system was adopted from the previous study (4).

| Name (plasmid) | (Architecture)<br>Sequence | Experiment used |
|---|---|---|
| Holin encoding toehold switch (pCOLADuet) | (pLlacO-1 – Toehold switch variant 1 – coupling element – S gene – *R gene* – Rz gene – T7term)<br>ATAAATGTGAGCGGATAACATTGACATTGTGAGCGGATAACAAGATAC<br>TGAGCACAGTAAGATAATGAAGGTAGGTATGTTAAACTTTAGAACAAG<br>ACAAGAAC**AGAGGAGA**TAAAGATGAACATACCTACGAACCTGGCGGC<br>AGCGCAAAAGACACTCGCAGAGGAGAGCGAGTAATGCCAGAAAAACA<br>TGACCTGTTGGCCGCCATTCTCGCGGCAAAGGAACAAGGCATCGGGGC<br>AATCCTTGCGTTTGCAATGGCGTACCTTCGCGGCAGATATAATGGCGGTG<br>CGTTTACAAAAACAGTAATCGACGCAACGATGTGCGCCATTATCGCCTG<br>GTTCATTCGTGACCTTCTCGACTTCGCCGGACTAAGTAGCAATCTCGCTT<br>ATATAACGAGCGTGTTTATCGGCTACATCGGTACTGACTCGATTGGTTCG<br>CTTATCAAACGCTTCGCTGCTAAAAAAGCCGGAGTAGAAG(*ATGGTAGAA*<br>*ATCAATAATCAACGTAAGGCGTTCCTCGATATGCTGGCGTGGTCGGAGGGAA*<br>*CTGATAACGGACGTCAGAAAACCAGAAATCATGGTTATGACGTCATTGTAGG*<br>*CGGAGAGCTATTTACTGATTACTCCGATCACCCTCGCAAACTTGTCACGCTAA*<br>*ACCCAAAACTCAAATCAACAGGCGCCGGACGCTACCAGCTTCTTTCCCGTTG*<br>*GTGGGATGCCTACCGCAAGCAGCTTGGCCTGAAAGACTTCTCTCCGAAAAG*<br>*TCAGGACGCTGTGGCATTGCAGCAGATTAAGGAGCGTGGCGCTTTACCTAT*<br>*GATTGATCGTGGTGATATCCGTCAGGCAATCGACCGTTGCAGCAATATCTGG*<br>*GCTTCACTGCCGGGCGCTGGTTATGGTCAGTTCGAGCATAAGGCTGACAGC*<br>*CTGATTGCAAAATTCAAAGAAGCGGGCGGAACGGTCAGAGAGATTGATGTAT*<br>*GA)*GCAGAGTCACCGCGATTATCTCCGCTCTGGTTATCTGCATCATCGTCT<br>GCCTGTCATGGGCTGTTAATCATTACCGTGATAACGCCATTACCTACAAA<br>GCCCAGCGCGACAAAAATGCCAGAGAACTGAAGCTGGCGAACGCGGC<br>AATTACTGACATGCAGATGCGTCAGCGTGATGTTGCTGCGCTCGATGCA<br>AAATACACGAAGGAGTTAGCTGATGCTAAAGCTGAAAATGATGCTCTGC<br>GTGATGATGTTGCCGCTGGTCGTCGTCGGTTGCACATCAAAGCAGTCTG<br>TCAGTCAGTGCGTGAAGCCACCACCGCCTCCGGCGTGGATAATGCAGC<br>CTCCCCCCGACTGGCAGACACCGCTGAACGGGATTATTTCACCCTCAGA<br>GAGAGGCTGATCACTATGCAAAAACAACTGGAAGGAACCCAGAAGTAT<br>ATTAATGAGCAGTGCAGATAGTCACTATGCAAAAACAACTGGAAGGAA<br>CCCAGAAGTATATTAATGAGCAGTGCAGATAGGATAAACCAGAGCGGCA<br>CGGCAAGCAGAGTATACGAGATTCGGTAGCCACCGCTGAGCAATAACTA<br>GCATAACCCCTTGGGGCCTCTAAACGGGTCTTGAGGGGTTTTTTG | **Figure 7, 8**<br><br>**Supplementary Figure S15, S16, S17, S18, S19, S20, S22, S23 and S24** |
| Trigger (pCDFDuet) | (pLlacO-1 – Trigger RNA – T7term)<br>ATAAATGTGAGCGGATAACATTGACATTGTGAGCGGATAACAAGATAC<br>TGAGCACGCTCGATCACTAATCTGATCGAGACGAACATACCTACCTTC<br>ATTATCTTACTTGTTAGCATAACCCCTTGGGGCCTCTAAACGGGTCTTG<br>AGGGGTTTTTTG | **Figure 7, 8**<br><br>**Supplementary Figure S15, S16, S17, S18, S19, S20, S22, S23 and S24** |
| Strong constitutive sRNA variant 1<br>(pET15b) | (pTlpA - Target sequence – Hfq scaffold – rho independent transcription terminator – T7term)<br>TTTAATTTGTTTGTTAGTTAGTTTATTTGTTGGTTTGTTTGTGTTATAATAT<br>GGGTTCTTGTCTTGTTCATTTGTAGAAATATTTTATTCGCCCCCGGAAGAT<br>CATTCCGGGGGCTTTTTTATTTAGCATAACCCCTTGGGGCCTCTAAACGG<br>GTCTTGAGGGGTTTTTTG | **Figure 7**<br><br>**Supplementary Figure S15, S16, S17, S18, and S19** |
| sRNA decoy (pET15b) | (pTlpA – Hfq scaffold – rho independent transcription terminator – T7term) | **Figure 7** |

| | | |
|---|---|---|
| | TTTAATTTGTTTGTTAGTTAGTTTATTTGTTGGTTTGTTTGTGTTATAATAT<br>ATTTGTAGAAATATTTTATTCGCCCCCGGAAGATCATTCCGGGGGCTTTT<br>TTATTTAGCATAACCCCTTGGGGCCTCTAAACGGGTCTTGAGGGGTTTTT<br>TG | **Supplementary Figure S15, S16, S17, S18, and S19** |
| GFP positive (pACYCDuet) | (pLtetO-1 – GFPmut3b - ssrA(ASV) – T7term)<br>TCCCTATCAGTGATAGAGATTGACATCCCTATCAGTGATAGATATACTG<br>AGCACACCTAAGGTAAATAAGGAGGAGTAACATGAAAGAGACGAACC<br>TGGCGGCAGCGCAAAAGATGCGTAAAGGAGAAGAACTTTTCACTGGA<br>GTTGTCCCAATTCTTGTTGAATTAGATGGTGATGTTAATGGGCACAAAT<br>TTTCTGTCAGTGGAGAGGGTGAAGGTGATGCAACATACGGAAAACTTA<br>CCCTTAAATTTATTTGCACTACTGGAAAACTACCTGTTCCGTGGCCAAC<br>ACTTGTCACTACTTTCGGTTATGGTGTTCAATGCTTTGCGAGATACCCA<br>GATCACATGAAACAGCATGACTTTTTCAAGAGTGCCATGCCCGAAGGT<br>TACGTACAGGAAAGAACTATATTTTTCAAAGATGACGGGAACTACAAG<br>ACACGTGCTGAAGTCAAGTTTGAAGGTGATACCCTTGTTAATAGAATC<br>GAGTTAAAAGGTATTGATTTTAAAGAAGATGGAAACATTCTTGGACAC<br>AAATTGGAATACAACTATAACTCACACAATGTATACATCATGGCAGAC<br>AAACAAAAGAATGGAATCAAAGTTAACTTCAAAATTAGACACAACATT<br>GAAGATGGAAGCGTTCAACTAGCAGACCATTATCAACAAAATACTCCG<br>ATTGGCGATGGCCCTGTCCTTTTACCAGACAACCATTACCTGTCCACAC<br>AATCTGCCCTTTCGAAAGATCCCAACGAAAAGAGAGACCACATGGTCC<br>TTCTTGAGTTTGTAACCGCTGCTGGGATTACACATGGCATGGATGAACT<br>ATACAAAAGGCCTGCAGCAAACGACGAAAACTACGCTGCATCAGTTTA<br>ATAATAATAAGATAAACCAGAGCGGCACGGCAAGCAGAGTATACGAG<br>ATTCGGTAGCCACCGCTGAGCAATAACTAGCATAACCCCTTGGGGCCT<br>CTAAACGGGTCTTGAGGGGTTTTTTG | **Figure 7, 8** |

**Supplementary Table S12.** Plasmid maps for the temperature responsive sRNA system in Figure **8** and **Supplementary Figure S21, S22, S23 and S24**.

| Name<br>[plasmid]<br>(architecture) | Sequence |
|---|---|
| Temperature responsive sRNA system<br><br>[pET15b]<br><br>(pTlpA – **target sequence** – Hfq scaffold – rho independent transcription terminator – T7term – proA – TlpA36 – T7term variant – AmpR – pBR322 origin – LacI) | AACGTGTACGGGCTATCTGGCTTTCGTTGCGCTTTAATTTGTTTGTTAGTTAGTTTATT<br>TGTTGGTTTGTTTGTGTTATAATATGG**GTTCTTGTCTTGTTC**ATTTGTAGAAATATTTT<br>ATTCGCCCCCGGAAGATCATTCCGGGGGCTTTTTTATTTAGCATAACCCCTTGGGGCC<br>TCTAAACGGGTCTTGAGGGGTTTTTTGCTGAAAGGAGGAACTATATCCGGATATCCC<br>GCAAGAGGCCCGGCAGTACCGGCATAACCAAGCCTATGCCTACAGCATCCAGGGTG<br>ACGGTGCCGAGGATGACGATGAGCGCATTGTTAGATTTCATACACGGCACAGCTAA<br>CACCACGTCGTCCCTATCTGCTGCCCTAGGTCTATGAGTGGTTGCTGGATAACTTTAC<br>GGGCATGCATAAGGCTCGTAGGCTATATTCAGGGAGACCACAACGGTTTCCCTCTAC<br>AAATAATTTTGTTTAACTTTGAAATAAGGAGGTAATACAAATGCGTCCGGCGACATA<br>CGAACCAGAACAGATTATTGAAGCAGGGCTGGCCCTGCAGGCTGAAGGACGGAATA<br>TCACCGGGTTCGCACTACGTAACCAGGTGGGTGGCGGCAATCCGACACGTCTCCGCC<br>AGATATGGGACGAATACCAGGCTTCACAGAGCACGGTCGTCACTGAACTCGTTGCC<br>GAGCTGCCAGTGGAAGTGGCTGAAGAAGTGAAGGCCGTCTCCGCCGCGCTGTCCGA<br>ACGCATCACCCAGCTGGCGACAGAACTGAATGACAAGGCGGTCCGGGCTGCAGAAC<br>GCCGGGTTGCGGAAGTCACGCGTGCTGCCGGTGAACAGACCGCACAGGCAGAGCGG<br>GAGCTGGCCGACGCCGCGCAGACAGTCGACGACCTGGAAGAAAAACTGGTTGAACT<br>GCAGGACAGATATGACAGTTTGACGCTGGCGCTGGAGTCAGAACGTTCACTGCGTC<br>AGCAGCATGATGTGGAGATGGCCCAGCTGAAAGAGCGTCTTGCGGCCGCTGAAGAG<br>AATACCCGTCAGCGAGAGGAACGGTATCAGGAGCAGAGGACAGTGCTGCAGGATGC<br>GCTTAATGCGGAGCAGGCACAGCACATAAACACGCGGGAAGACCAGCAGAAACGA<br>CTGGAGCAAATTTCTGCCGAAGCTAATGCGCGTACAGAAGAACTGAAGTCTGAACG<br>CGATAAAGTCAATACTCTCCTTACCCGCCTTGAATCGCAGGAAAATGCGCTGGCCTC<br>AGAACGTCAGCAGCATCTGGCCACCCGCGAAACGCTGCAGCAACGCCTCGAGCAGG<br>CCATCGCTGACACGCAGGCGCGCGCCGGTGAGATTGCACTTGAACGTGACAGAGTC<br>AGCAGCCTCACCGCAAGGCTGGAATCGCAGGAAAAGGCCTCCTCGGAGCAACTGGT |

GCGTATGGGCAGTGAAATAGCCAGTCTGACAGAGCGTTGCACACAGCTGGAAAACC
AGCGTGATGATGCCCGTCTGGAGACGATGGGGGAGAAAGAAACGGTCGCGGCACTG
CGTGGTGAGGCTGAAGCCCTGAAGCGTCAGAACCAGTCACTGATGGCGGCGCTTTC
AGGCAATAAACAGACCGGTGGCCAGAATGCGTGAGGTACCCTCGAGTCTGGTAAAG
AAACCGCTGCTGCGAAATTTGAACGCCAGCACATGGACTCGTCTACTAGCGCAGCTT
AATTAACCTAGGCTGCTGCCACCGCTGAGCAATAATAGCATAAACAGATAGGCCCTC
TTCGGAGGGCCTATCTGTTTTTTTTTGTGCCTGACTGCGTTAGCAATTTAACTGTGAT
AAACTACCGCATTAAAGCTTATCGATGATAAGCTGTCAAACATGAGAATTCTTGAAG
ACGAAAGGGCCTCGTGATACGCCTATTTTTATAGGTTAATGTCATGATAATAATGGT
TTCTTAGACGTCAGGTGGCACTTTTCGGGGAAATGTGCGCGGAACCCCTATTTGTTT
ATTTTTCTAAATACATTCAAATATGTATCCGCTCATGAGACAATAACCCTGATAAAT
GCTTCAATAATATTGAAAAAGGAAGAGTATGAGTATTCAACATTTCCGTGTCGCCCT
TATTCCCTTTTTTGCGGCATTTTGCCTTCCTGTTTTTGCTCACCCAGAAACGCTGGTGA
AAGTAAAAGATGCTGAAGATCAGTTGGGTGCACGAGTGGGTTACATCGAACTGGAT
CTCAACAGCGGTAAGATCCTTGAGAGTTTTCGCCCCGAAGAACGTTTTCCAATGATG
AGCACTTTTAAAGTTCTGCTATGTGGCGCGGTATTATCCCGTGTTGACGCCGGGCAA
GAGCAACTCGGTCGCCGCATACACTATTCTCAGAATGACTTGGTTGAGTACTCACCA
GTCACAGAAAAGCATCTTACGGATGGCATGACAGTAAGAGAATTATGCAGTGCTGC
CATAACCATGAGTGATAACACTGCGGCCAACTTACTTCTGACAACGATCGGAGGACC
GAAGGAGCTAACCGCTTTTTTGCACAACATGGGGGATCATGTAACTCGCCTTGATCG
TTGGGAACCGGAGCTGAATGAAGCCATACCAAACGACGAGCGTGACACCACGATGC
CTGCAGCAATGGCAACAACGTTGCGCAAACTATTAACTGGCGAACTACTTACTCTAG
CTTCCCGGCAACAATTAATAGACTGGATGGAGGCGGATAAAGTTGCAGGACCACTT
CTGCGCTCGGCCCTTCCGGCTGGCTGGTTTATTGCTGATAAATCTGGAGCCGGTGAG
CGTGGGTCTCGCGGTATCATTGCAGCACTGGGGCCAGATGGTAAGCCCTCCCGTATC
GTAGTTATCTACACGACGGGGAGTCAGGCAACTATGGATGAACGAAATAGACAGAT
CGCTGAGATAGGTGCCTCACTGATTAAGCATTGGTAACTGTCAGACCAAGTTTACTC
ATATATACTTTAGATTGATTTAAAACTTCATTTTTAATTTAAAAGGATCTAGGTGAAG
ATCCTTTTTGATAATCTCATGACCAAAATCCCTTAACGTGAGTTTTCGTTCCACTGAG
CGTCAGACCCCGTAGAAAAGATCAAAGGATCTTCTTGAGATCCTTTTTTTCTGCGCG
TAATCTGCTGCTTGCAAACAAAAAAACCACCGCTACCAGCGGTGGTTTGTTTGCCGG
ATCAAGAGCTACCAACTCTTTTTCCGAAGGTAACTGGCTTCAGCAGAGCGCAGATAC
CAAATACTGTCCTTCTAGTGTAGCCGTAGTTAGGCCACCACTTCAAGAACTCTGTAG
CACCGCCTACATACCTCGCTCTGCTAATCCTGTTACCAGTGGCTGCTGCCAGTGGCG
ATAAGTCGTGTCTTACCGGGTTGGACTCAAGACGATAGTTACCGGATAAGGCGCAGC
GGTCGGGCTGAACGGGGGGTTCGTGCACACAGCCCAGCTTGGAGCGAACGACCTAC
ACCGAACTGAGATACCTACAGCGTGAGCTATGAGAAAGCGCCACGCTTCCCGAAGG
GAGAAAGGCGGACAGGTATCCGGTAAGCGGCAGGGTCGGAACAGGAGAGCGCACG
AGGGAGCTTCCAGGGGGAAACGCCTGGTATCTTTATAGTCCTGTCGGGTTTCGCCAC
CTCTGACTTGAGCGTCGATTTTTGTGATGCTCGTCAGGGGGGCGGAGCCTATGGAAA
AACGCCAGCAACGCGGCCTTTTTACGGTTCCTGGCCTTTTGCTGGCCTTTTGCTCACA
TGTTCTTTCCTGCGTTATCCCCTGATTCTGTGGATAACCGTATTACCGCCTTTGAGTG
AGCTGATACCGCTCGCCGCAGCCGAACGACCGAGCGCAGCGAGTCAGTGAGCGAGG
AAGCGGAAGAGCGCCTGATGCGGTATTTTCTCCTTACGCATCTGTGCGGTATTTCAC
ACCGCATATATGGTGCACTCTCAGTACAATCTGCTCTGATGCCGCATAGTTAAGCCA
GTATACACTCCGCTATCGCTACGTGACTGGGTCATGGCTGCGCCCCGACACCCGCCA
ACACCCGCTGACGCGCCCTGACGGGCTTGTCTGCTCCCGGCATCCGCTTACAGACAA
GCTGTGACCGTCTCCGGGAGCTGCATGTGTCAGAGGTTTTCACCGTCATCACCGAAA
CGCGCGAGGCAGCTGCGGTAAAGCTCATCAGCGTGGTCGTGAAGCGATTCACAGAT
GTCTGCCTGTTCATCCGCGTCCAGCTCGTTGAGTTTCTCCAGAAGCGTTAATGTCTGG
CTTCTGATAAAGCGGGCCATGTTAAGGGCGGTTTTTTCCTGTTTGGTCACTGATGCCT
CCGTGTAAGGGGGATTTCTGTTCATGGGGGTAATGATACCGATGAAACGAGAGAGG
ATGCTCACGATACGGGTTACTGATGATGAACATGCCCGGTTACTGGAACGTTGTGAG
GGTAAACAACTGGCGGTATGGATGCGGCGGGACCAGAGAAAAATCACTCAGGGTCA
ATGCCAGCGCTTCGTTAATACAGATGTAGGTGTTCCACAGGGTAGCCAGCAGCATCC
TGCGATGCAGATCCGGAACATAATGGTGCAGGGCGCTGACTTCCGCGTTTCCAGACT
TTACGAAACACGGAAACCGAAGACCATTCATGTTGTTGCTCAGGTCGCAGACGTTTT
GCAGCAGCAGTCGCTTCACGTTCGCTCGCGTATCGGTGATTCATTCTGCTAACCAGT
AAGGCAACCCCGCCAGCCTAGCCGGGTCCTCAACGACAGGAGCACGATCATGCGCA
CCCGTGGCCAGGACCCAACGCTGCCCGAGATGCGCCGCGTGCGGCTGCTGGAGATG
GCGGACGCGATGGATATGTTCTGCCAAGGGTTGGTTTGCGCATTCACAGTTCTCCGC
AAGAATTGATTGGCTCCAATTCTTGGAGTGGTGAATCCGTTAGCGAGGTGCCGCCGG
CTTCCATTCAGGTCGAGGTGGCCCGGCTCCATGCACCGCGACGCAACGCGGGGAGG
CAGACAAGGTATAGGGCGGCGCCTACAATCCATGCCAACCCGTTCCATGTGCTCGCC
GAGGCGGCATAAATCGCCGTGACGATCAGCGGTCCAGTGATCGAAGTTAGGCTGGT

| | |
|---|---|
| | AAGAGCCGCGAGCGATCCTTGAAGCTGTCCCTGATGGTCGTCATCTACCTGCCTGGA<br>CAGCATGGCCTGCAACGCGGGCATCCCGATGCCGCCGGAAGCGAGAAGAATCATAA<br>TGGGGAAGGCCATCCAGCCTCGCGTCGCGAACGCCAGCAAGACGTAGCCCAGCGCG<br>TCGGCCGCCATGCCGGCGATAATGGCCTGCTTCTCGCCGAAACGTTTGGTGGCGGGA<br>CCAGTGACGAAGGCTTGAGCGAGGGCGTGCAAGATTCCGAATACCGCAAGCGACAG<br>GCCGATCATCGTCGCGCTCCAGCGAAAGCGGTCCTCGCCGAAAATGACCCAGAGCG<br>CTGCCGGCACCTGTCCTACGAGTTGCATGATAAAGAAGACAGTCATAAGTGCGGCG<br>ACGATAGTCATGCCCCGCGCCCACCGGAAGGAGCTGACTGGGTTGAAGGCTCTCAA<br>GGGCATCGGTCGAGATCCCGGTGCCTAATGAGTGAGCTAACTTACATTAATTGCGTT<br>GCGCTCACTGCCCGCTTTCCAGTCGGGAAACCTGTCGTGCCAGCTGCATTAATGAAT<br>CGGCCAACGCGCGGGGAGAGGCGGTTTGCGTATTGGGCGCCAGGGTGGTTTTTCTTT<br>TCACCAGTGAGACGGGCAACAGCTGATTGCCCTTCACCGCCTGGCCCTGAGAGAGTT<br>GCAGCAAGCGGTCCACGCTGGTTTGCCCCAGCAGGCGAAAATCCTGTTTGATGGTGG<br>TTAACGGCGGGATATAACATGAGCTGTCTTCGGTATCGTCGTATCCCACTACCGAGA<br>TATCCGCACCAACGCGCAGCCCGGACTCGGTAATGGCGCGCATTGCGCCCAGCGCC<br>ATCTGATCGTTGGCAACCAGCATCGCAGTGGGAACGATGCCCTCATTCAGCATTTGC<br>ATGGTTTGTTGAAAACCGGACATGGCACTCCAGTCGCCTTCCCGTTCCGCTATCGGC<br>TGAATTTGATTGCGAGTGAGATATTTATGCCAGCCAGCCAGACGCAGACGCGCCGA<br>GACAGAACTTAATGGGCCCGCTAACAGCGCGATTTGCTGGTGACCCAATGCGACCA<br>GATGCTCCACGCCCAGTCGCGTACCGTCTTCATGGGAGAAAATAATACTGTTGATGG<br>GTGTCTGGTCAGAGACATCAAGAAATAACGCCGGAACATTAGTGCAGGCAGCTTCC<br>ACAGCAATGGCATCCTGGTCATCCAGCGGATAGTTAATGATCAGCCCACTGACGCGT<br>TGCGCGAGAAGATTGTGCACCGCCGCTTTACAGGCTTCGACGCCGCTTCGTTCTACC<br>ATCGACACCACCACGCTGGCACCCAGTTGATCGGCGCGAGATTTAATCGCCGCGACA<br>ATTTGCGACGGCGCGTGCAGGGCCAGACTGGAGGTGGCAACGCCAATCAGCAACGA<br>CTGTTTGCCCGCCAGTTGTTGTGCCACGCGGTTGGGAATGTAATTCAGCTCCGCCATC<br>GCCGCTTCCACTTTTTCCCGCGTTTTCGCAGAAACGTGGCTGGCCTGGTTCACCACGC<br>GGGAAACGGTCTGATAAGAGACACCGGCATACTCTGCGACATCGTATAACGTTACT<br>GGTTTCACATTCACCACCCTGAATTGACTCTCTTCCGGGCGCTATCATGCCATACCGC<br>GAAAGGTTTTGCGCCATTCGATGGTGTCCGGGATCTCGACGCTCTCCCTTATGCGAC<br>TCCTGCATTAGGAAGCAGCCCAGTAGTAGGTTGAGGCCGTTGAGCACCGCCGCCGC<br>AAGGAATGGTGCATGCAAGGAGATGGCGCCCAACAGTCCCCCGGCCACGGGGCCTG<br>CCACCATACCCACGCCGAAACAAGCGCTCATGAGCCCGAAGTGGCGAGCCCGATCT<br>TCCCCATCGGTGATGTCGGCGATATAGGCGCCAGCAACCGCACCTGTGGCGCCGGTG<br>ATGCCGGCCACGATGCGTCCGGCGTAGAGGATCGAGATCTCG |

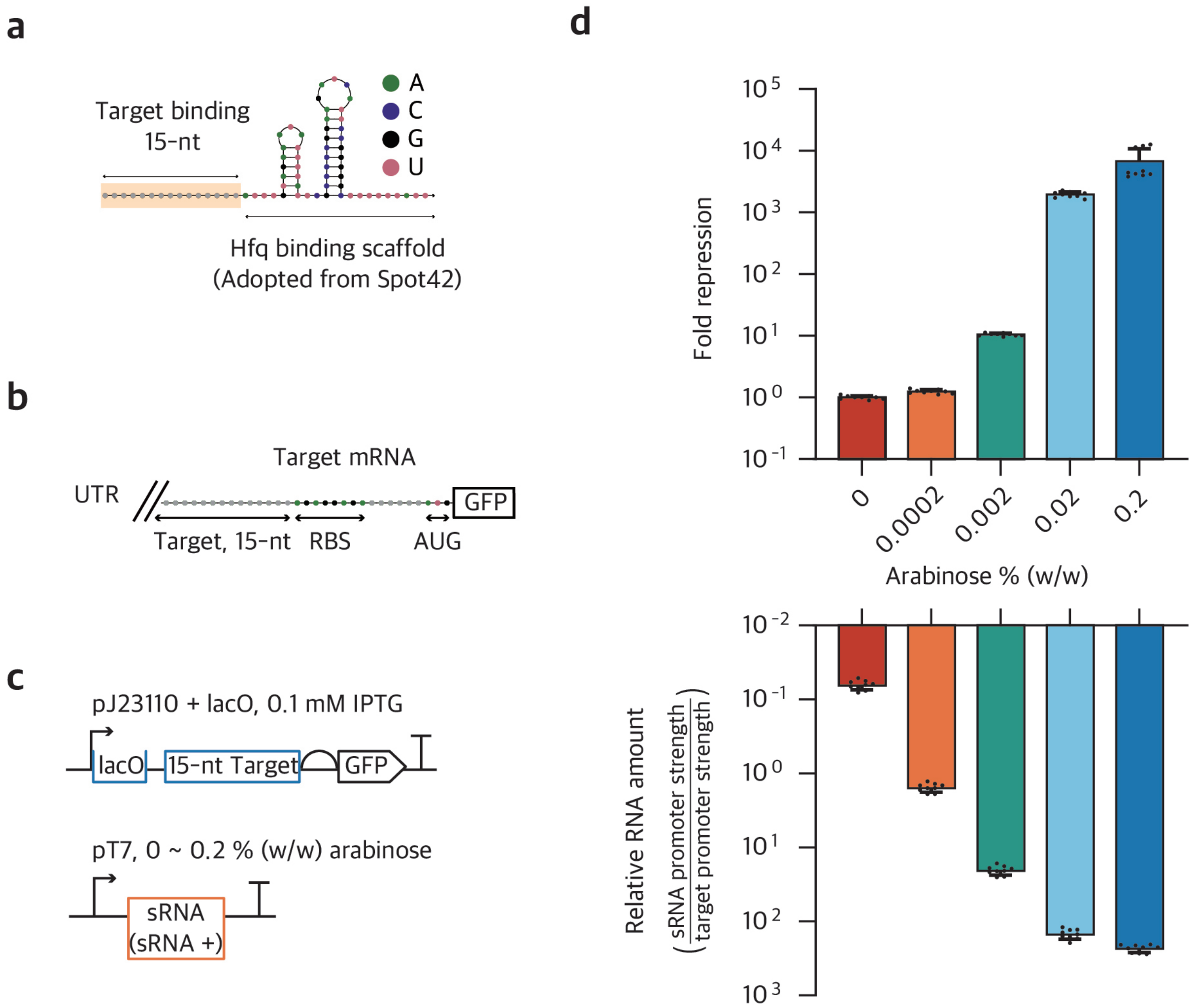


**Supplementary Figure S1.** Secondary structures of the synthetic sRNA and its target mRNA, and sRNA-mediated GFP expression repression.

(**a**) Secondary structure of a synthetic sRNA used in this study. (**b**) Secondary structure of a target mRNA which was used in **Supplementary Figure S1**. The secondary structure of **Supplementary Figure S1A** and **S1B** was analyzed using NUPACK (1-3). The linker sequence indicated rigid RNA sequence between toehold switch and GFP for reducing steric hinderance. (**c**) Expression cassettes of Target RNA and sRNA. Target RNA is expressed from a strong constitutive promoter pJ23110 with lacO for IPTG-inducible expression. sRNA is under the control of T7 promoter, which in turn is induced by l-arabinose (ara) in E. coli BL21-AITM that harbors a genomic copy of T7 RNA polymerase under the pBAD promoter. (**d**) Comparison of relative sRNA abundance and fold repression strength. The fluorescence of cells was measured via flow cytometry 4 h 30 min after induction. IPTG concentration was fixed at 0.1 mM, while the arabinose concentration was varied at 0, 0.0002, 0.002, 0.02, 0.2 % (w/w). Relative sRNA abundance was calculated as the ratio of sRNA promoter strength to target promoter strength. The target mRNA levels were measured by performing qPCR on *gfp* RNA. However, due to the short length of sRNA, direct quantification via qPCR was not feasible. Instead, a *gfp* coding sequence was inserted into the sRNA expression cassette, and qPCR was performed on the *gfp* RNA. During the measurement of sRNA promoter strength, the gene regulated under the switch RNA was replaced with *mcherry* instead of *gfp*. The amounts of *gfp* RNA were subsequently normalized using

16S rRNA. The number of biological replicates is three. The fold repression and relative RNA amount was calculated for all possible combinations between groups, resulting in nine cases. Error bars indicated standard deviation.

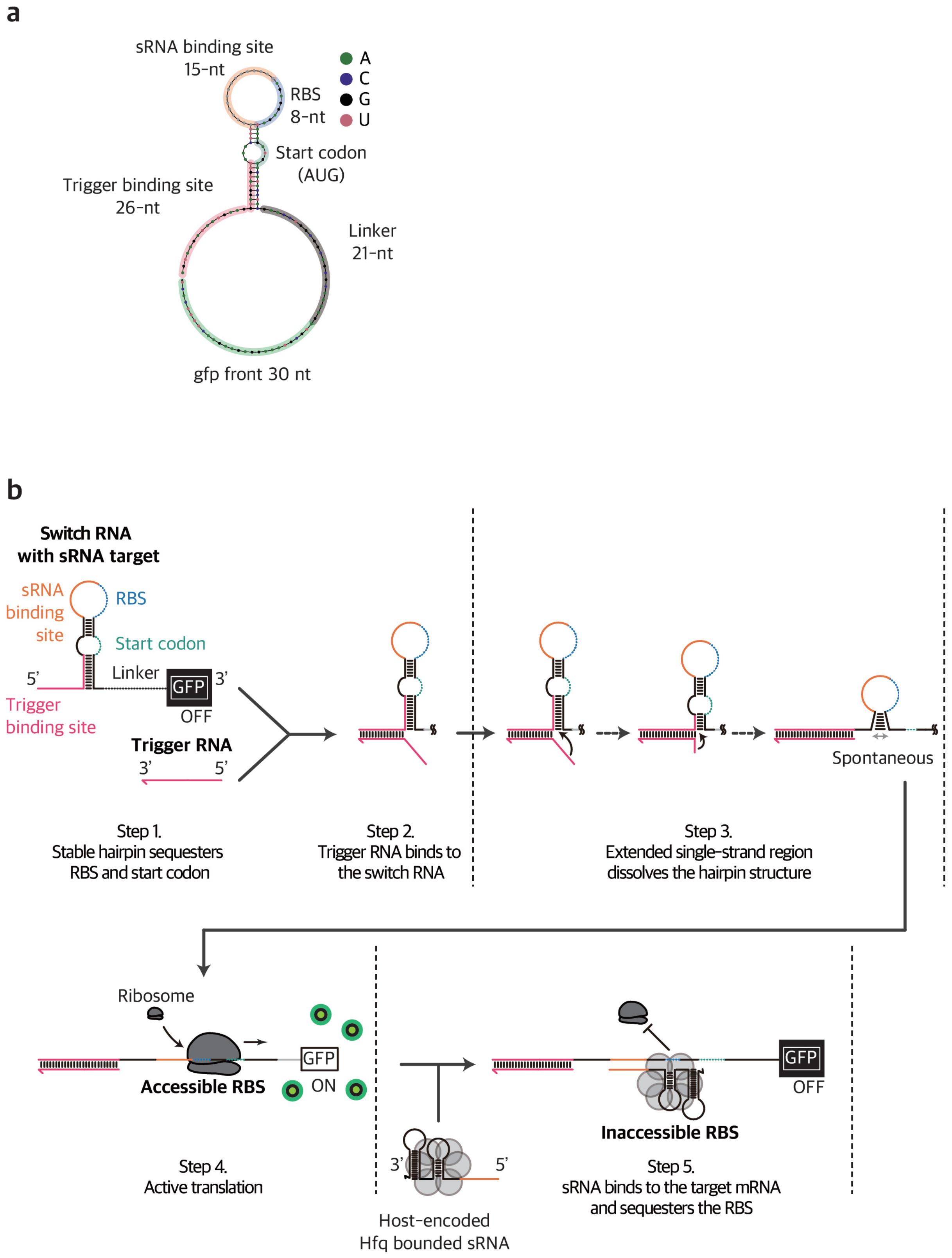


**Supplementary Figure S2.** Schematics of bacterial small RNA (sRNA) mediated post-transcriptional repression of a toehold switch.

(**a**) Secondary structure of a toehold switch used in this study (5). (**b**) Stepwise operational schematic of a toehold switch. In its initial state, a unique hairpin structure sequesters the ribosome binding site (RBS) and the start codon (Step 1). When a cognate trigger RNA binds to the toehold switch (Step 2), extended single stranded domain of a trigger RNA resolves the hairpin structure via strand displacement (Step 3). Freely accessible RBS allows strong translation initiation (Step 4). However, when sRNA with host-encoded Hfq binds to the target mRNA, the RBS is re-sequestered, blocking translational initiation (Step 5).

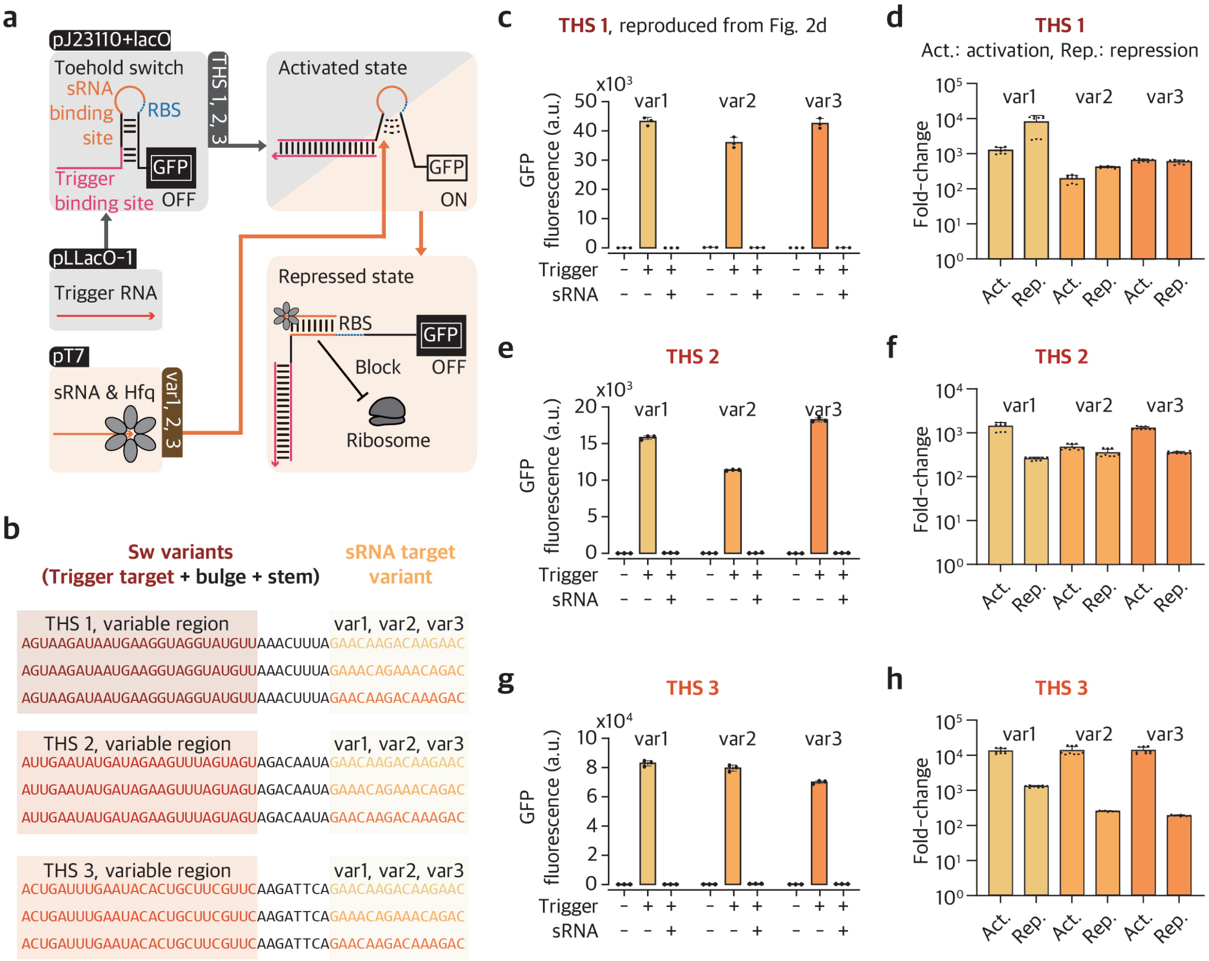


**Supplementary Figure S3.** Post-transcriptional repression of toehold switch variants using sRNA variant 1, 2, and 3.

(**a**) Schematics representation of sRNA-mediated toehold switch (THS) repression. (**b**) The trigger binding domain and sRNA binding site with sequence variations. (**c**) Application of sRNA variants to toehold switch variant 1. This figure was reproduced from **Figure 2d**. (**d**) Activation fold change upon trigger introduction, and repression fold change upon sRNA introduction. (**e**) Application of sRNA variants to toehold switch variant 2. (**f**) Activation fold change upon trigger introduction, and repression fold change upon sRNA introduction. (**g**) Application of sRNA variants to toehold switch variant 3. (**h**) Activation fold change of THS upon trigger introduction, and repression fold change upon sRNA introduction. Experiments and measurements were performed as described in Figure 2. The number of

biological replicates was three. Fold-change was calculated for all possible combinations between groups, resulting in nine cases. Error bars indicate standard deviation.

**a**

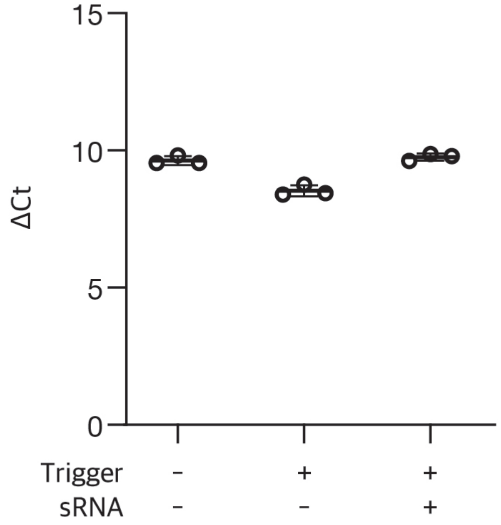


**b**

| Trigger | sRNA | ΔCt |
|---|---|---|
| - | - | 9.63 ± 0.16 |
| + | - | 8.53 ± 0.20 |
| + | + | 9.76 ± 0.13 |

**c**

Welch's t-test

| Trigger/sRNA | Trigger/sRNA | P-value |
|---|---|---|
| -/- | +/- | **(0.002) |
| -/- | +/+ | ns(0.4247) |
| +/- | +/+ | **(0.0017) |

**Supplementary Figure S4** Quantification of a toehold switch RNA using qPCR.

(**a** and **b**) Changes in switch RNA levels in **Figure 2b** (sRNA variant 1). Switch RNA levels were quantified as *gfp* mRNA, normalized using 16S rRNA. The normalized expression (ΔCt) were calculated as follows: ΔCt = Ct(GFP)-Ct(16S rRNA). (**c**) p-values based on Welch's t-test. Minus symbol indicates the expression of decoy RNA, while plus symbol indicates the expression of either the cognate trigger or cognate sRNA. The number of biological replicates was three. Error bars indicate standard deviation.

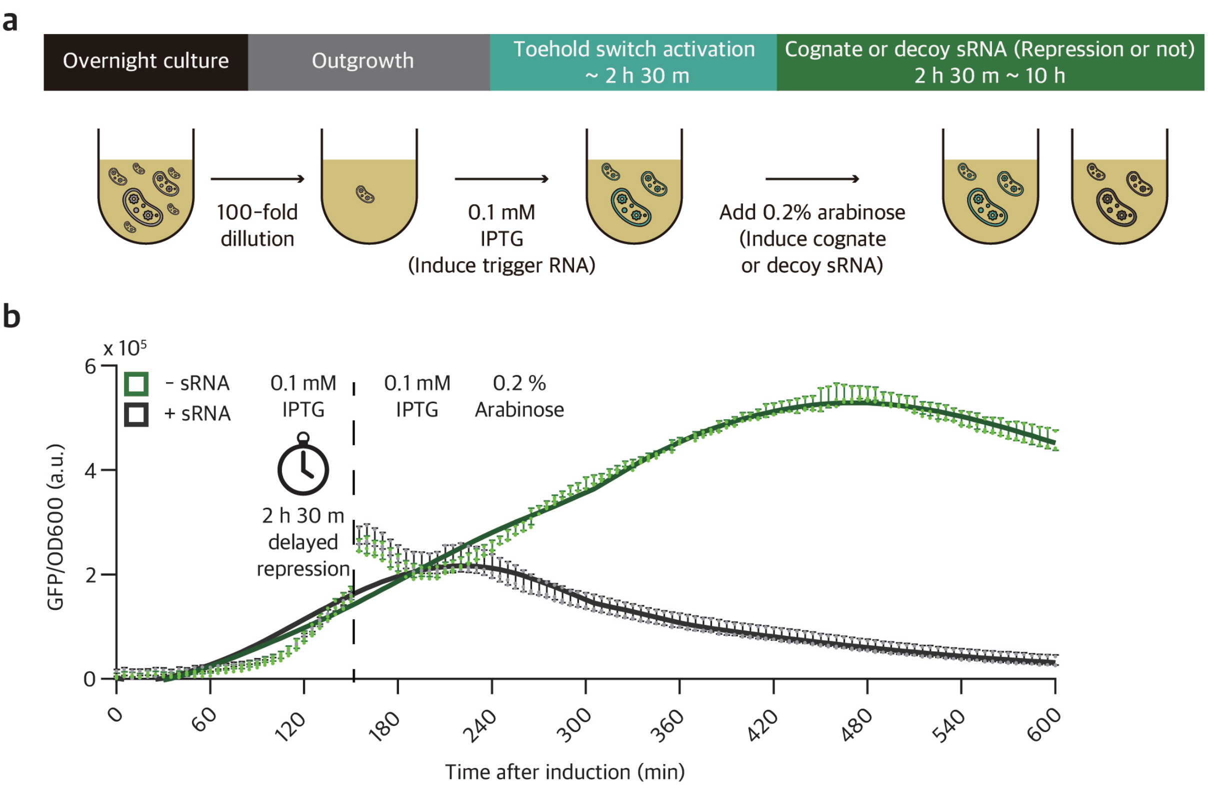


**Supplementary Figure S5** Sequential activation and repression of a toehold switch using sRNA.

(**a**) Schematics representation of the experimental procedure. GFP fluorescence and OD600 were measured at 5-minute intervals during incubation using a plate reader. The plasmid configurations are identical to those in **Figure 2b** (sRNA variant 1). (**b**) The toehold switch system was induced with 0.1 mM IPTG, and sRNA expression was initiated 2 h 30 m later with 0.2% arabinose. Green and gray dots represent each replicate, while green and gray lines represent smoothed lines obtained using 30 neighbors. -sRNA indicates the expression of decoy RNA, while +sRNA indicates the expression of cognate sRNA. The number of biological replicates was three. Error bars indicate standard deviation.

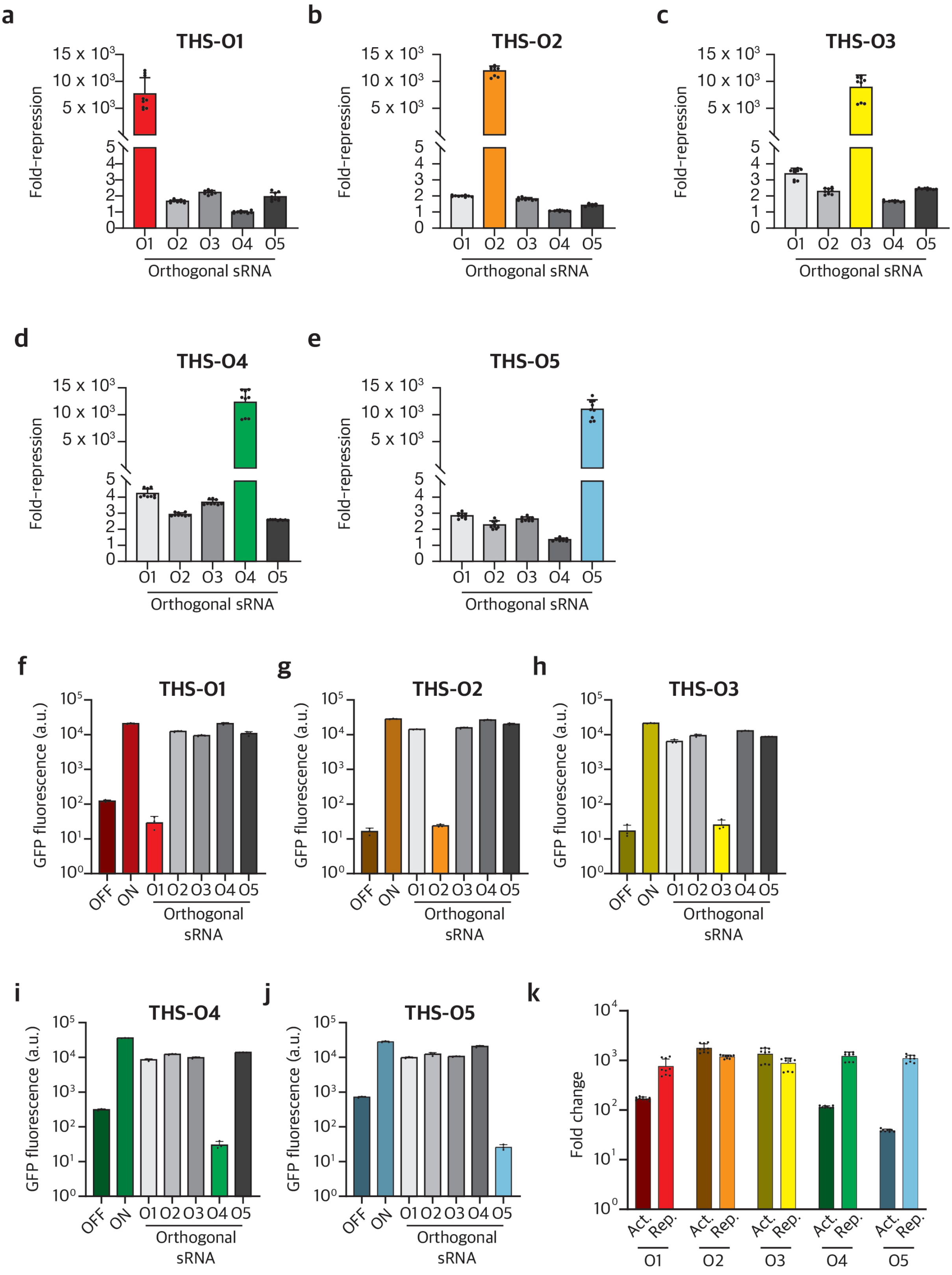


**Supplementary Figure S6.** Cross reactivity within the 5X5 orthogonal sRNA pool

(**a–e**) GFP expression fold-repression by five orthogonal sRNAs: (a) orthogonal switch 1, (b) orthogonal switch 2, (c) orthogonal switch 3, (d) orthogonal switch 4, and (e) orthogonal switch 5. **(f–**

**j**) GFP expression levels for (f) orthogonal switch 1, (g) orthogonal switch 2, (h) orthogonal switch 3, (i) orthogonal switch 4, and (j) orthogonal switch 5. (**k**) Fold-activations and fold-repressions for the five orthogonal switches. In (a–e), colored bars represent responsiveness to the cognate sRNA, while gray bars represent responsiveness to the non-cognate sRNAs. Measurements were performed as described in Figure 2. Three biological replicates were performed. Fold-repression was calculated for all possible combinations between groups, resulting in nine cases. Error bars indicate standard deviation.

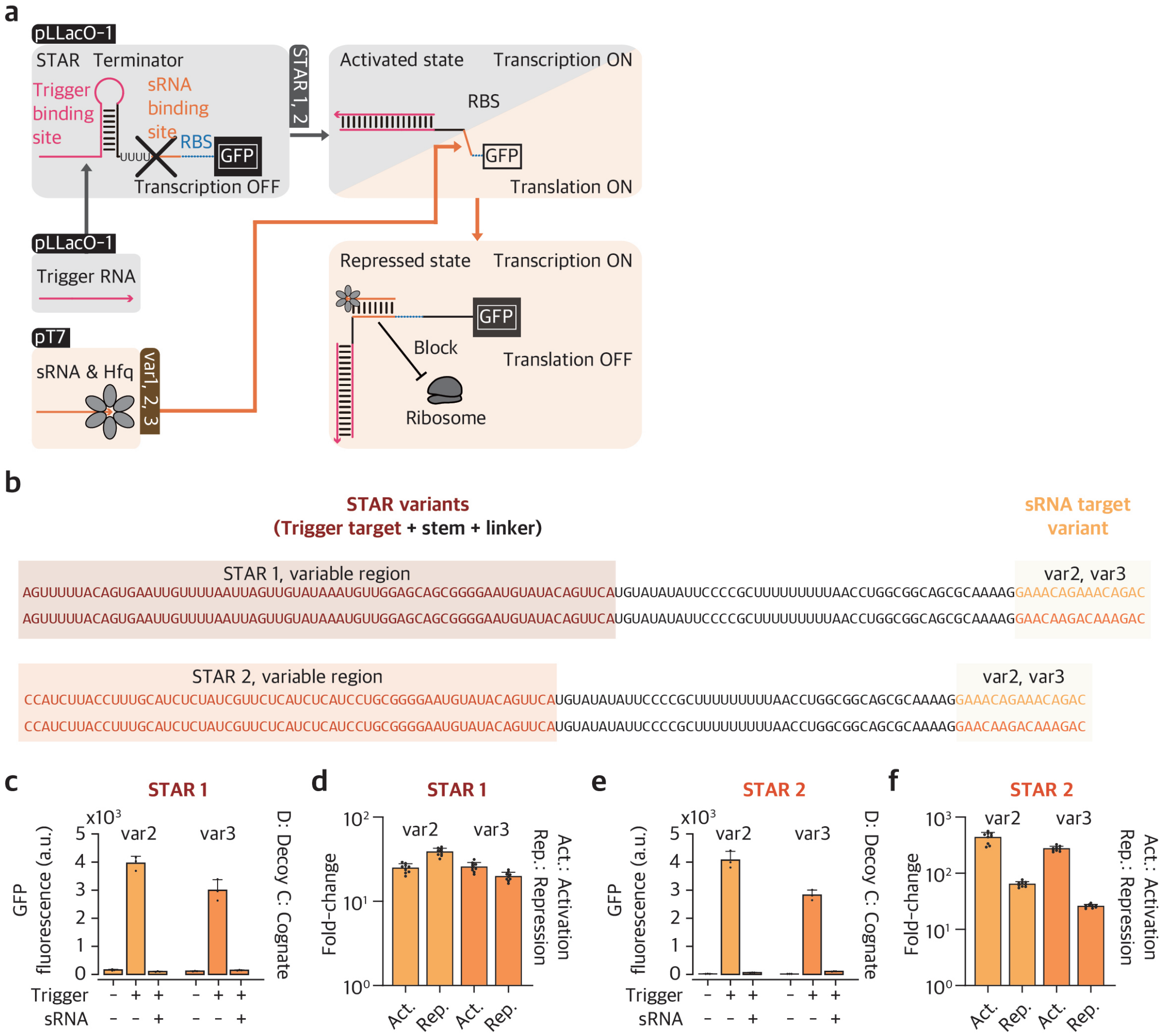


**Supplementary Figure S7.** Post-transcriptional repression of small transcription activating RNA (STAR) variants using sRNA variant 2 and 3.

(**a**) Schematics representation of sRNA-mediated post-transcriptional of STAR (11,12). The STAR system functions as a rho-independent transcription terminator in the absence of trigger RNA. However, when a cognate trigger RNA binds, the terminator structure is resolved through strand displacement. (**b**) Sequences of STAR variants with diversified trigger binding sites and sRNA target sites. (**c**) Application of sRNA variants to STAR variant 1. (**d**) Activation fold change upon trigger introduction and repression fold change upon sRNA introduction. (**e**) Application of sRNA variants to STAR variant 2. (**f**) Activation fold change upon trigger introduction and repression fold change upon sRNA introduction.

Minus symbol indicates the expression of decoy RNA, while plus symbol indicates the expression of either the cognate trigger or cognate sRNA. Experiments and measurements were performed as described in Figure 2. The number of biological replicates was three. Fold-change was calculated for all possible combinations between groups, resulting in nine cases. Error bars indicate standard deviation.

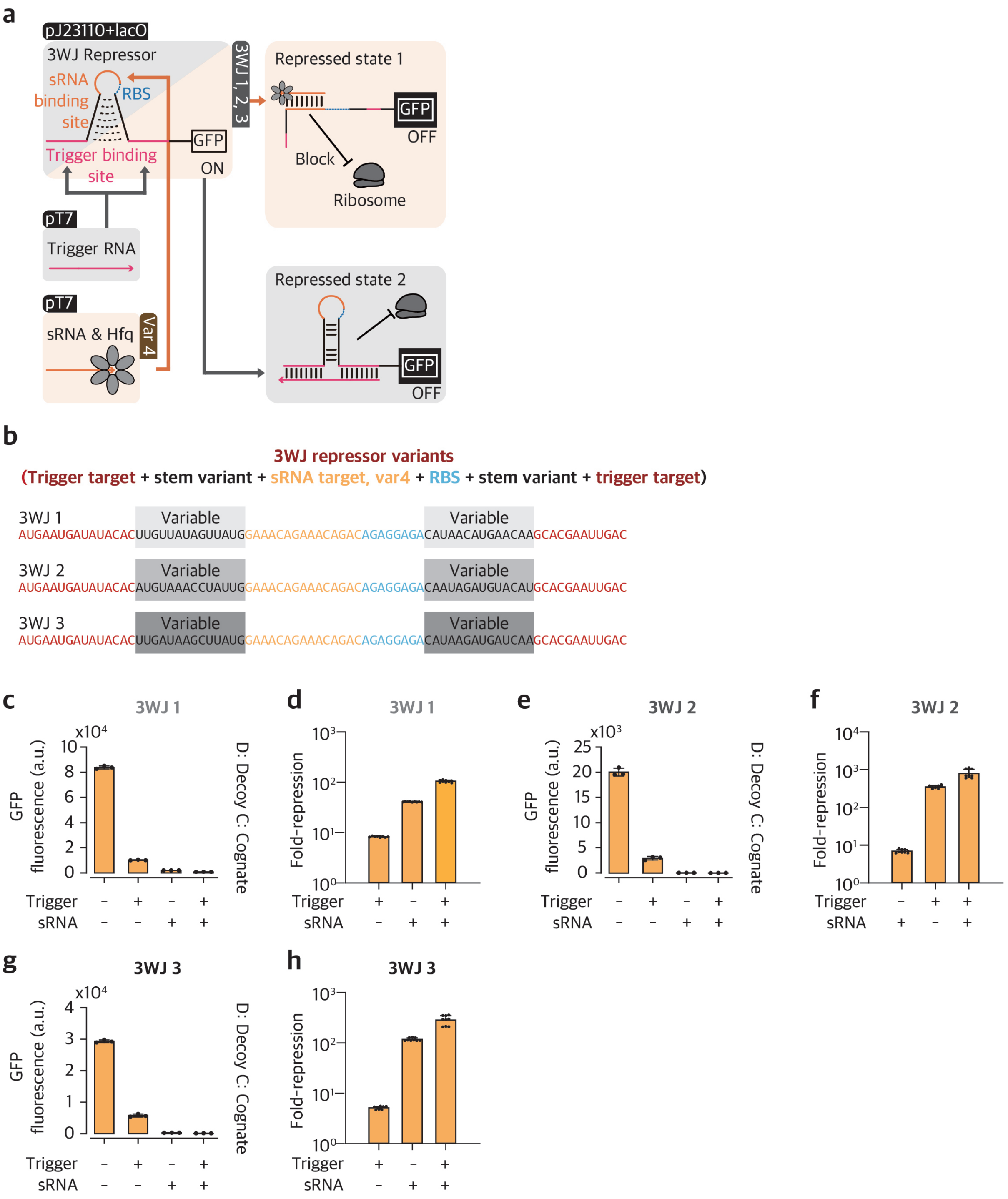

**Supplementary Figure S8.** Post-transcriptional repression of 3-way junction (3WJ) repressor variants using sRNA variant 4.

(**a**) Schematics representation of sRNA-mediated post-transcriptional repression of a 3WJ repressor (10). In its initial state, the 3WJ leaves the RBS freely accessible. However, when the trigger RNA binds, structural changes form a new hairpin structure, sequestering the RBS. (**b**) Sequences of 3WJ repressor variants with diversified hairpin stem sequences. (**c**) Application of sRNA variant 4 to 3WJ repressor variant 1. (**d**) Repression fold-change upon trigger introduction and/or sRNA introduction for (c). (**e**) Application of sRNA variant 4 to 3WJ repressor variant 2. (**f**) Repression fold-change upon trigger introduction and/or sRNA introduction for (e). (**g**) Application of sRNA variant 4 to 3WJ repressor variant 3. (**h**) Repression fold-change upon trigger introduction and/or sRNA introduction for (g). Minus symbol indicates the expression of decoy RNA, while plus symbol indicates the expression of either the cognate trigger or cognate sRNA. Experiments and measurements were performed as described in Figure 2. The number of biological replicates was three. Fold-change was calculated for all possible combinations between groups, resulting in nine cases. Error bars indicate standard deviation.

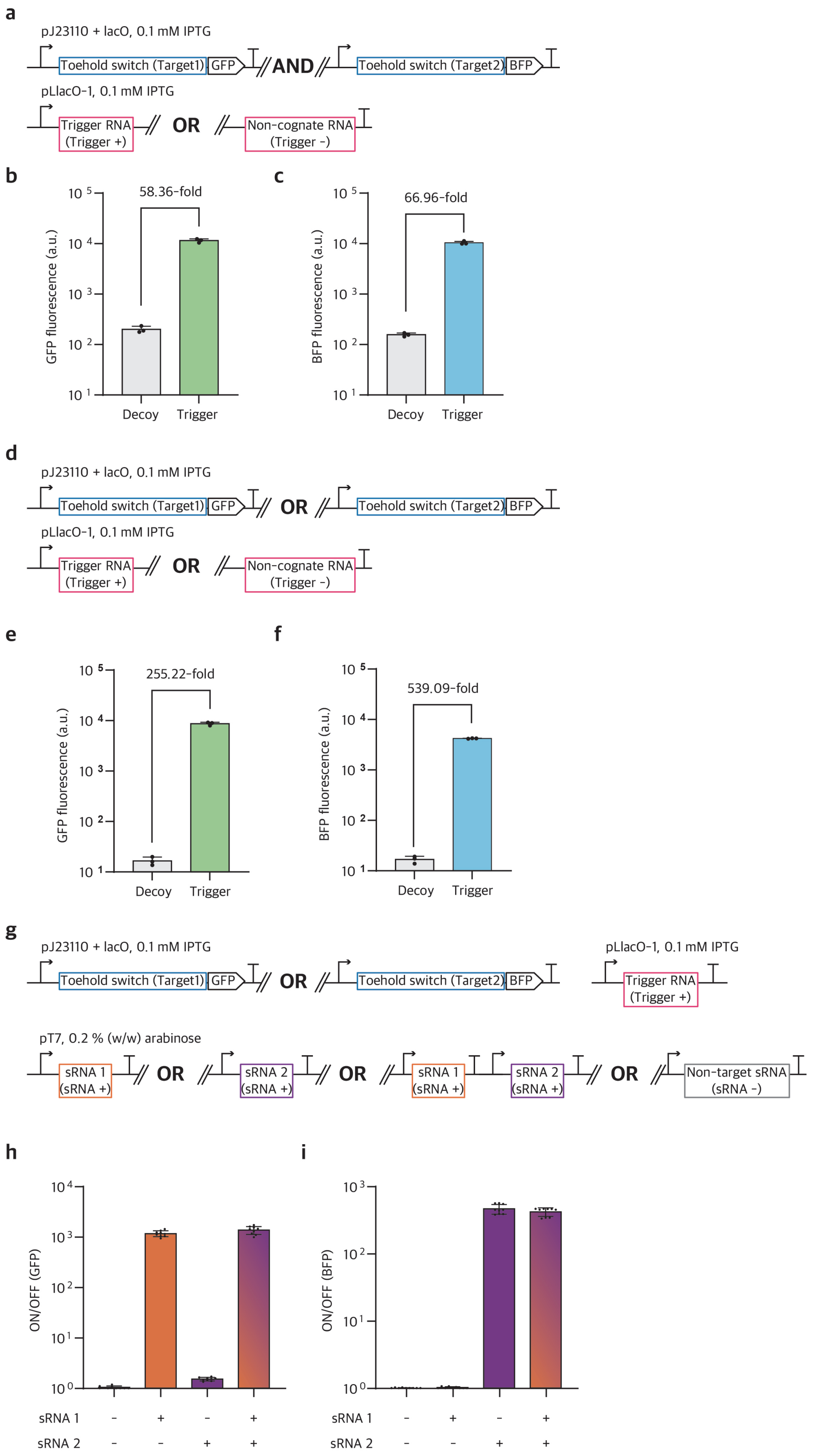
a
pJ23110 + lacO, 0.1 mM IPTG
Toehold switch (Target1)
GFP
AND
Toehold switch (Target2)
BFP
pLlacO-1, 0.1 mM IPTG
Trigger RNA (Trigger +)
OR
Non-cognate RNA (Trigger -)
b
58.36-fold
GFP fluorescence (a.u.)
Decoy
Trigger
c
66.96-fold
BFP fluorescence (a.u.)
Decoy
Trigger
d
pJ23110 + lacO, 0.1 mM IPTG
Toehold switch (Target1)
GFP
OR
Toehold switch (Target2)
BFP
pLlacO-1, 0.1 mM IPTG
Trigger RNA (Trigger +)
OR
Non-cognate RNA (Trigger -)
e
255.22-fold
GFP fluorescence (a.u.)
Decoy
Trigger
f
539.09-fold
BFP fluorescence (a.u.)
Decoy
Trigger
g
pJ23110 + lacO, 0.1 mM IPTG
Toehold switch (Target1)
GFP
OR
Toehold switch (Target2)
BFP
pLlacO-1, 0.1 mM IPTG
Trigger RNA (Trigger +)
pT7, 0.2 % (w/w) arabinose
sRNA 1 (sRNA +)
OR
sRNA 2 (sRNA +)
OR
sRNA 1 (sRNA +)
sRNA 2 (sRNA +)
OR
Non-target sRNA (sRNA -)
h
ON/OFF (GFP)
sRNA 1 - + - +
sRNA 2 - - + +
i
ON/OFF (BFP)
sRNA 1 - + - +
sRNA 2 - - + +

**Supplementary Figure S9.** Characterization of orthogonal regulation in dual-gene expression and microbial co-culture systems. (**a**) Schematic of a dual toehold-switch design expressing two independent modules (GFP-regulating THS and BFP-regulating THS) within a single cell. (**b, c**) GFP (b) and BFP (c) fluorescence outputs in single cells under either cognate Trigger RNA or Decoy RNA conditions. (**d**) Schematic of two distinct cell populations, each harboring a single switch (GFP-regulating THS or BFP-regulating THS). (**e, f**) Fluorescence outputs of the GFP-regulating switch (e) and the BFP-regulating switch (f) under Trigger RNA or Decoy RNA conditions. (**g**) Schematic of the mixed co-culture system in which the two cell types were combined for orthogonality testing. (**h, i**) Fold repression of GFP (h) and BFP (i) in co-culture upon introduction of sRNA 1, sRNA 2, or both, demonstrating orthogonal and combinatorial control across distinct populations. Experiments and measurements were performed as described in Figure 4. Error bars indicate the standard deviation of three biological replicates.

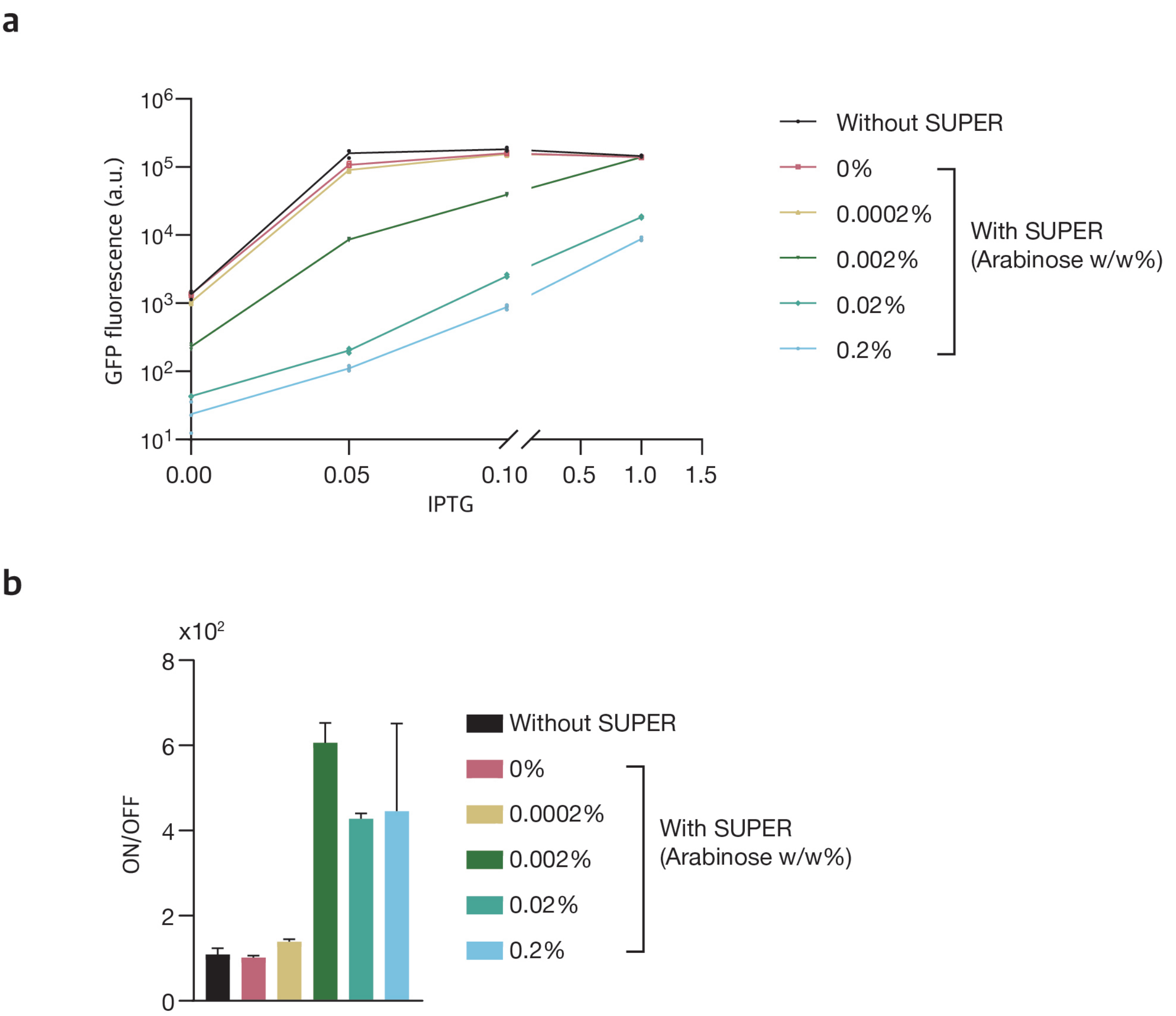


**Supplementary Figure S10.** Leakage reduction and performance enhancement of a THS using SUPER.

(**a**) Screening of arabinose concentrations to identify the optimal sRNA expression balance. Increasing the sRNA expression level led to greater leakage reduction at 0 mM IPTG but also caused a decrease in the ON level. (**b**) Enhancement of fold activation upon application of SUPER. The number of biological replicates was three. Error bars indicate standard deviation. Fold activation was calculated for all possible combinations between groups, resulting in nine cases.

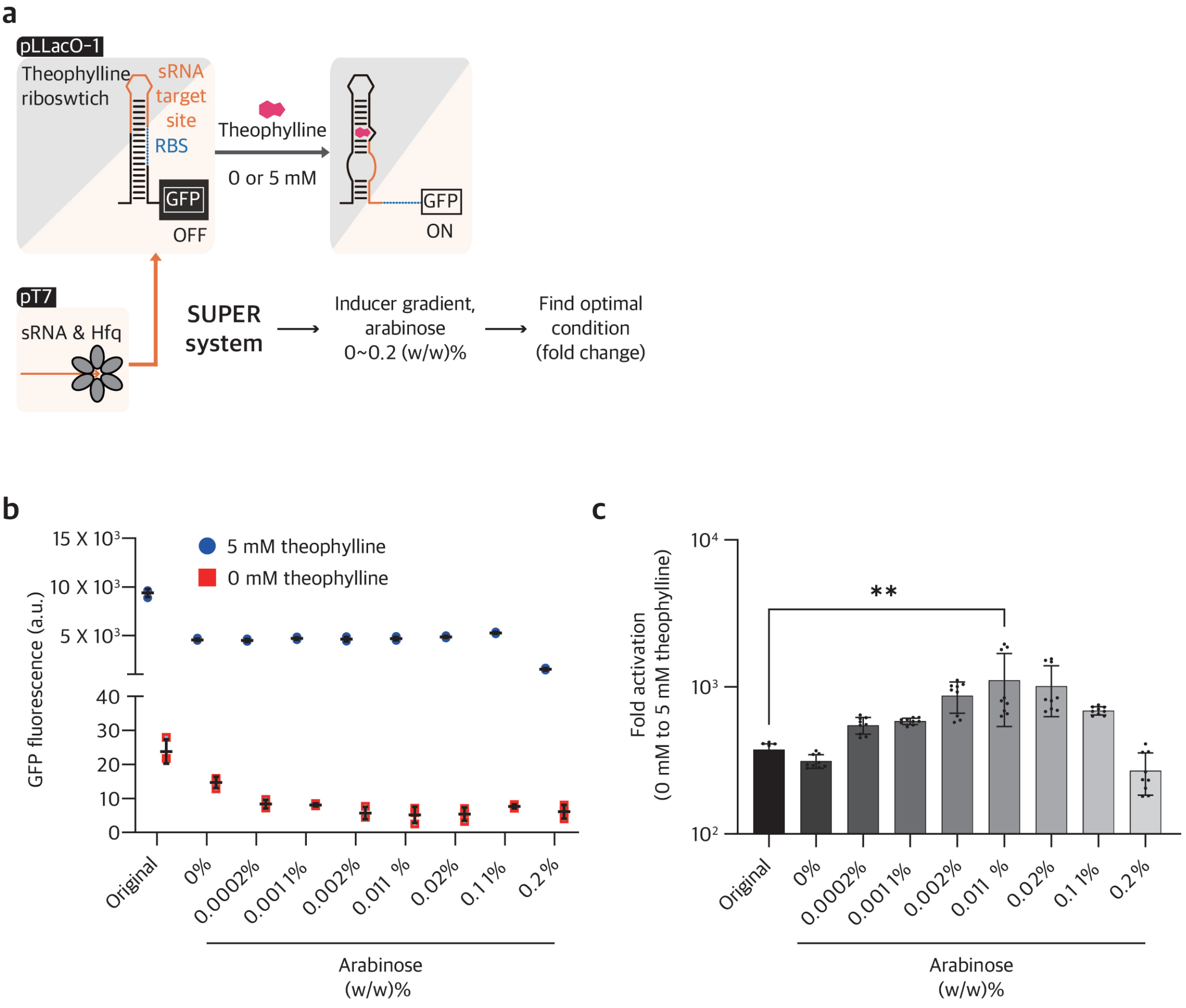


**Supplementary Figure S11.** The leakage reduction and performance enhancement of a cis-acting theophylline riboswitch using SUPER

(**a**) Operational schematic of the cis-acting theophylline riboswitch (6). In its initial state, a unique hairpin structure sequesters the RBS. However, when theophylline binds to the aptamer domain, it undergoes structural change. As a result, RBS is exposed and allowed for translation initiation. The theophylline riboswitch remained unchanged from the reported sequence, and a synthetic sRNA was designed to target the 15-nt region immediately upstream of the RBS. (**b**) Screening of arabinose concentrations to identify the optimal sRNA balance. (**c**) Enhancement of fold activation following the application of SUPER. Experiments and measurements were performed as described in Figure 6. The number of biological replicates was three. Error bars indicate standard deviation. Fold activation was calculated for all possible combinations between groups, resulting in nine cases. Based on Welch's *t*-tests, $^{**}P < 0.01$.

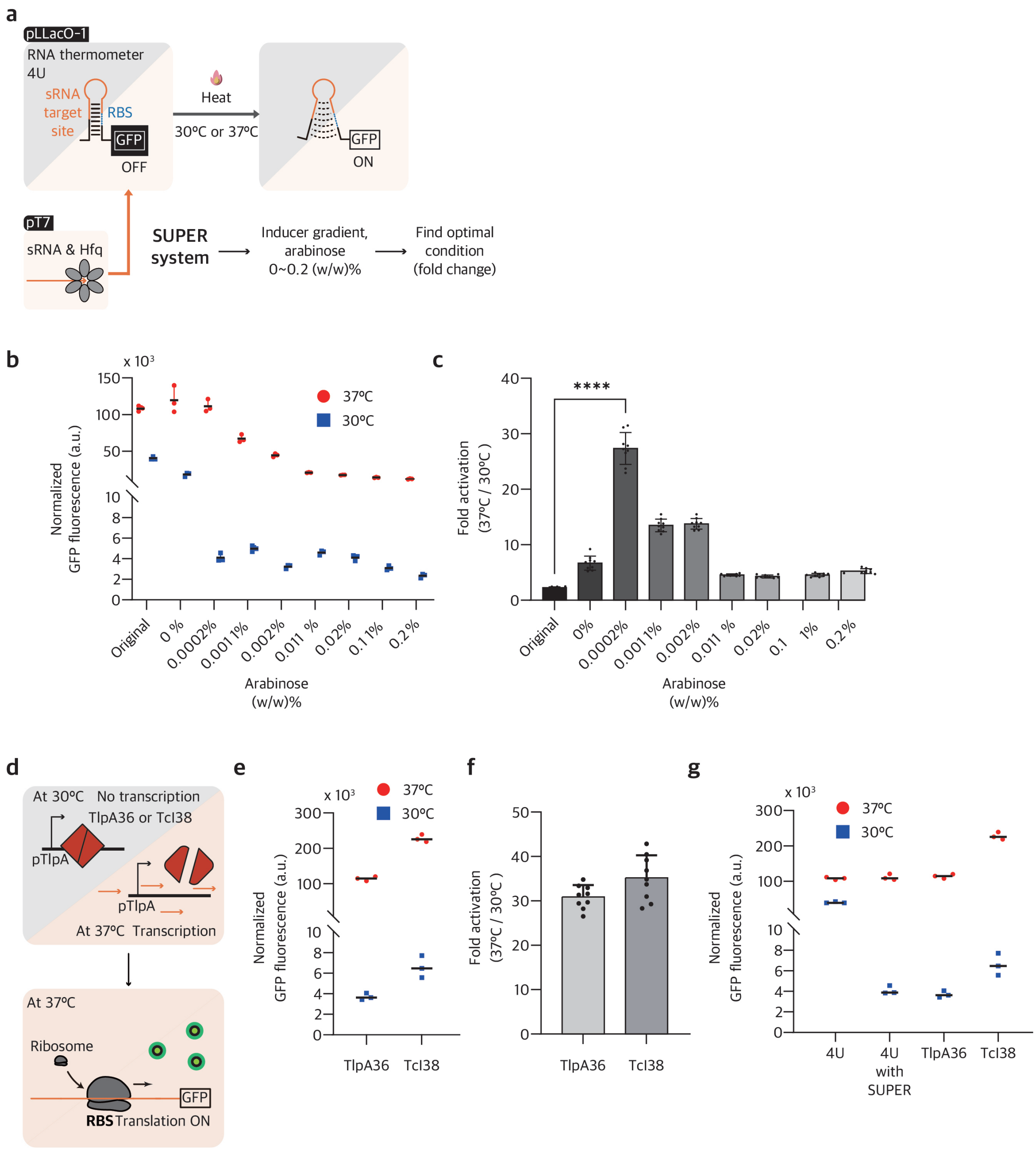


**Supplementary Figure S12.** The leakage reduction and performance enhancement of the *Salmonella* 4U RNA thermometer (RNAT) using SUPER

(**a**) Operational schematic of the *Salmonella* 4U RNAT (7). In its initial state, a unique hairpin structure sequesters the RBS. However, when the temperature increases, it undergoes structural change. As a result, RBS is exposed and allowed for translation initiation. The *Salmonella* 4U RNAT remained unchanged from the reported sequence, and a synthetic sRNA was designed to target the 15 nt region immediately upstream of the RBS. (**b**) Screening of arabinose concentrations to identify the optimal sRNA balance. (**c**) Enhancement of fold activation following the application of SUPER. **(d)** Schematic of TlpA36 protein-based thermometer. At 30°C, TlpA36 (or Tcl38) represses transcription from pTlpA

promoter, preventing downstream gene expression. At 37°C, the repressor dissociates, allowing transcription and expression of downstream GFP reporter. The transcript is subsequently translated, enabling ribosome binding to the ribosome binding site (RBS) and GFP production. (**e**) Temperature dependent changes in GFP fluorescence levels using well-known temperature-responsive transcription factors (9). (**f**) Fold activation of well-known temperature-responsive transcription factors. (**g**) Comparison of SUPER-applied 4U RNA thermometer with well-known temperature-responsive transcription factors. RNA and protein thermometer experiments and measurements were performed as described in Figure 6. The number of biological replicates was three. Error bars indicate standard deviation. Fold activation was calculated for all possible combinations between groups, resulting in nine cases. Based on Welch's *t*-tests, ****$P < 0.0001$. Temperature-dependent differences in GFP production were normalized using GFP positive control.

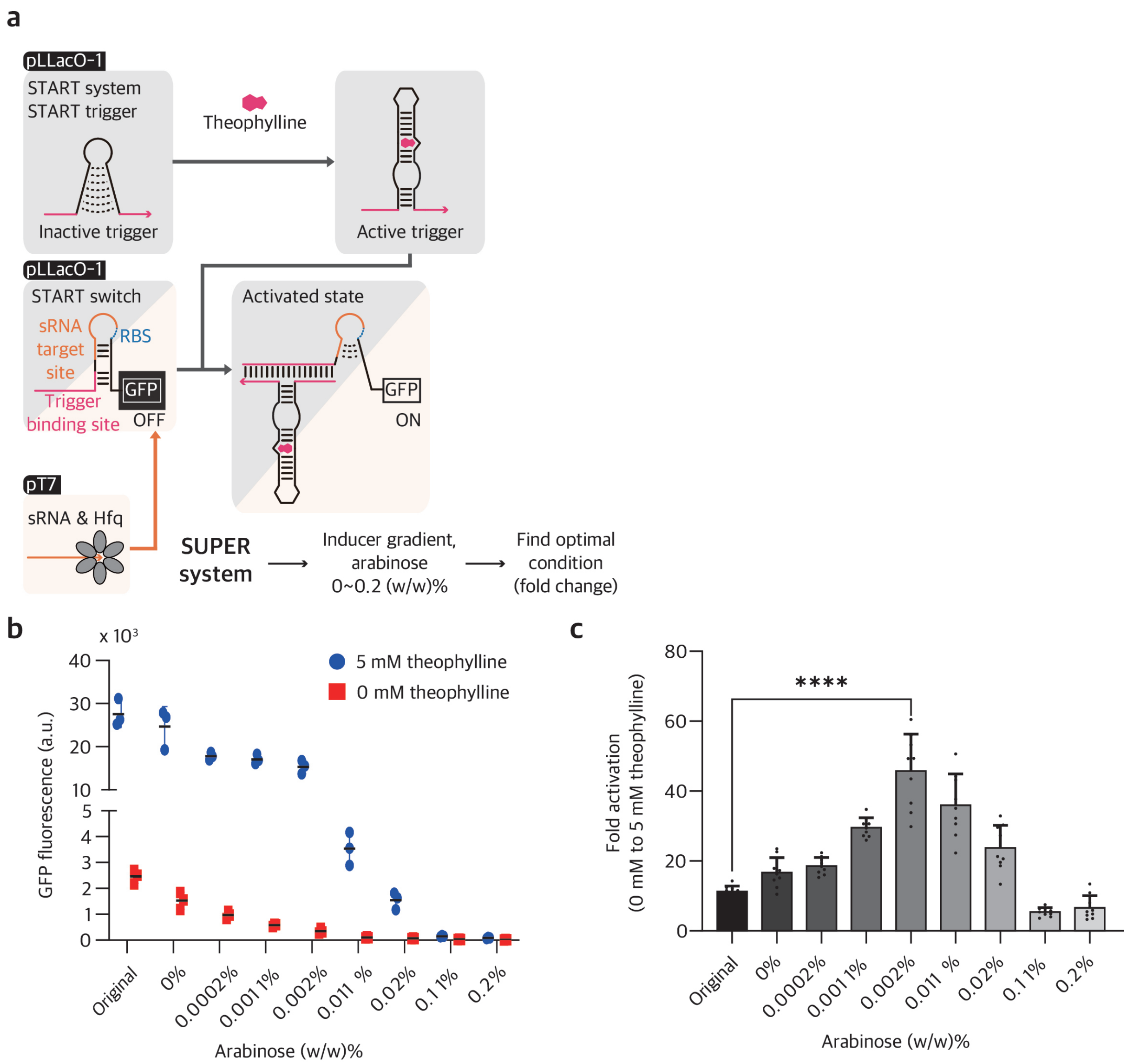


**Supplementary Figure S13.** The leakage reduction and performance enhancement of theophylline responsive START (Synthetic Trans-Acting Riboswitch with Triggering RNA) system using SUPER.

(**a**) Operational schematic of the theophylline dependent START system (8). Unlike conventional riboswitches, the START system consists of two components. First, the trigger RNA contains an aptamer sequence, and thereby, it undergoes a structural change in the presence of a theophylline molecule. Second, the START-switch interacts with a ligand-bound START-trigger, resulting in its own structural change, which leads to the exposure of the sequestered RBS. (**b**) Screening of arabinose concentrations to identify the optimal sRNA balance for theophylline responsive START system. (**c**) Enhancement of fold activation in the theophylline START system following the application of SUPER. Experiments and measurements were performed as described in Figure 6. The number of biological replicates was three. Error bars indicate standard deviation. Fold activation was calculated for all possible combinations between groups, resulting in nine cases. Based on Welch's *t*-tests, ****$P < 0.0001$.

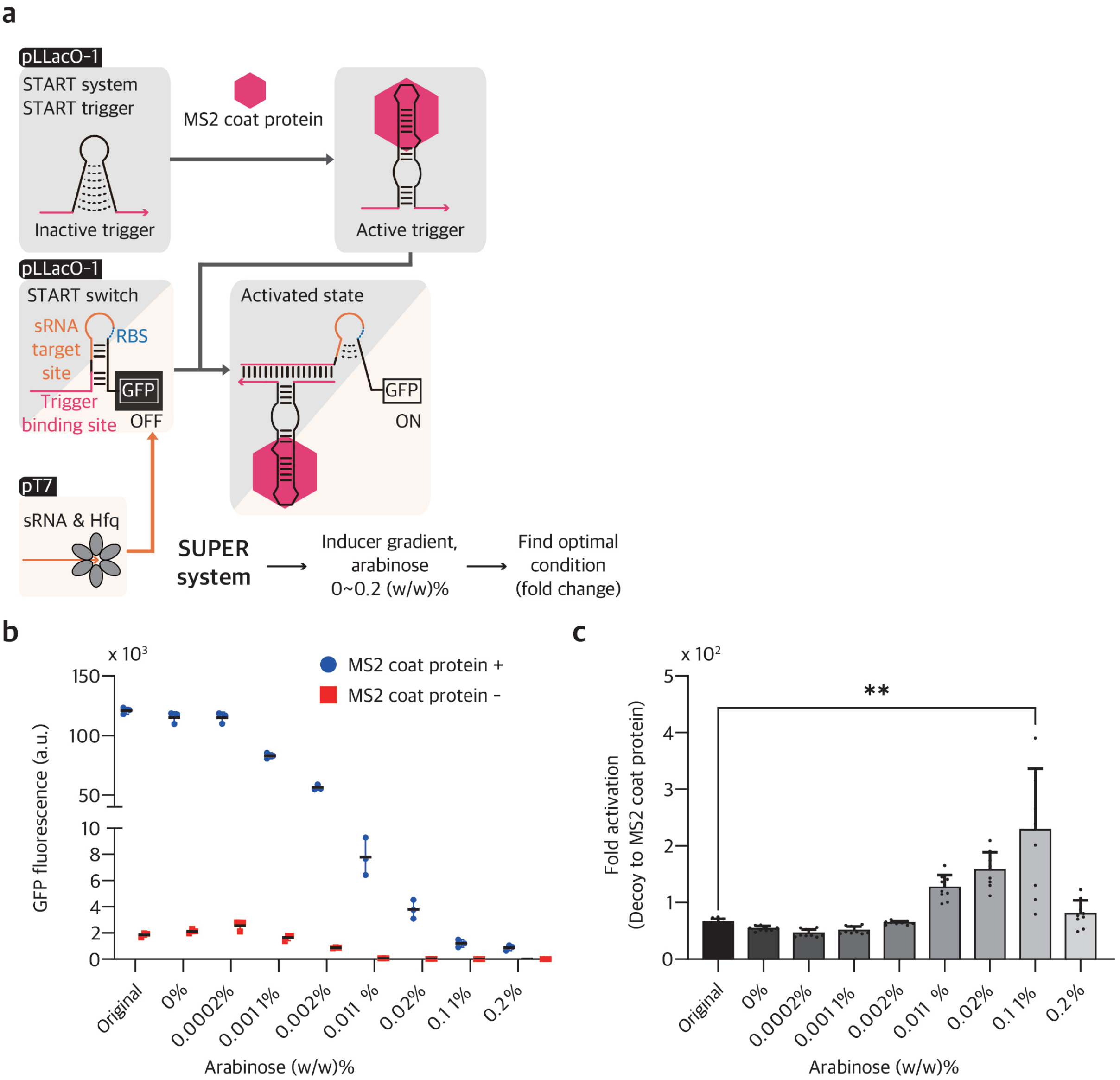
a
pLLacO-1
START system
START trigger
Inactive trigger
MS2 coat protein
Active trigger
pLLacO-1
START switch
sRNA
target
site
RBS
GFP
Trigger
binding site
OFF
Activated state
GFP
ON
pT7
sRNA & Hfq
SUPER
system
Inducer gradient,
arabinose
0~0.2 (w/w)%
Find optimal
condition
(fold change)
b
x 10³
MS2 coat protein +
MS2 coat protein -
GFP fluorescence (a.u.)
150
100
50
10
8
6
4
2
0
Original
0%
0.0002%
0.001%
0.002%
0.011 %
0.02%
0.1%
0.2%
Arabinose (w/w)%
c
x 10²
**
Fold activation
(Decoy to MS2 coat protein)
5
4
3
2
1
0
Original
0%
0.0002%
0.001%
0.002%
0.011 %
0.02%
0.1%
0.2%
Arabinose (w/w)%

**Supplementary Figure S14.** The leakage reduction and performance enhancement of MS2 START (Synthetic Trans-Acting Riboswitch with Triggering RNA) system using SUPER.

(**a**) Operational schematic of the MS2 coat protein dependent START system (8). Unlike conventional riboswitches, the START system consists of two components. First, the trigger RNA contains an aptamer sequence, and thereby, it undergoes a structural change in the presence of a MS2 coat protein. Second, the START-switch interacts with a ligand-bound START-trigger, resulting in its own structural change, which leads to the exposure of the sequestered RBS. (**b**) Screening of arabinose concentrations to identify the optimal sRNA balance for MS2 START system. (**c**) Enhancement of fold activation in the MS2 START system following the application of SUPER. Experiments and measurements were performed as described in Figure 6. The number of biological replicates was three. Error bars indicate standard deviation. Fold activation was calculated for all possible combinations between groups, resulting in nine cases. Based on Welch's *t*-tests, $^{**}P < 0.01$.

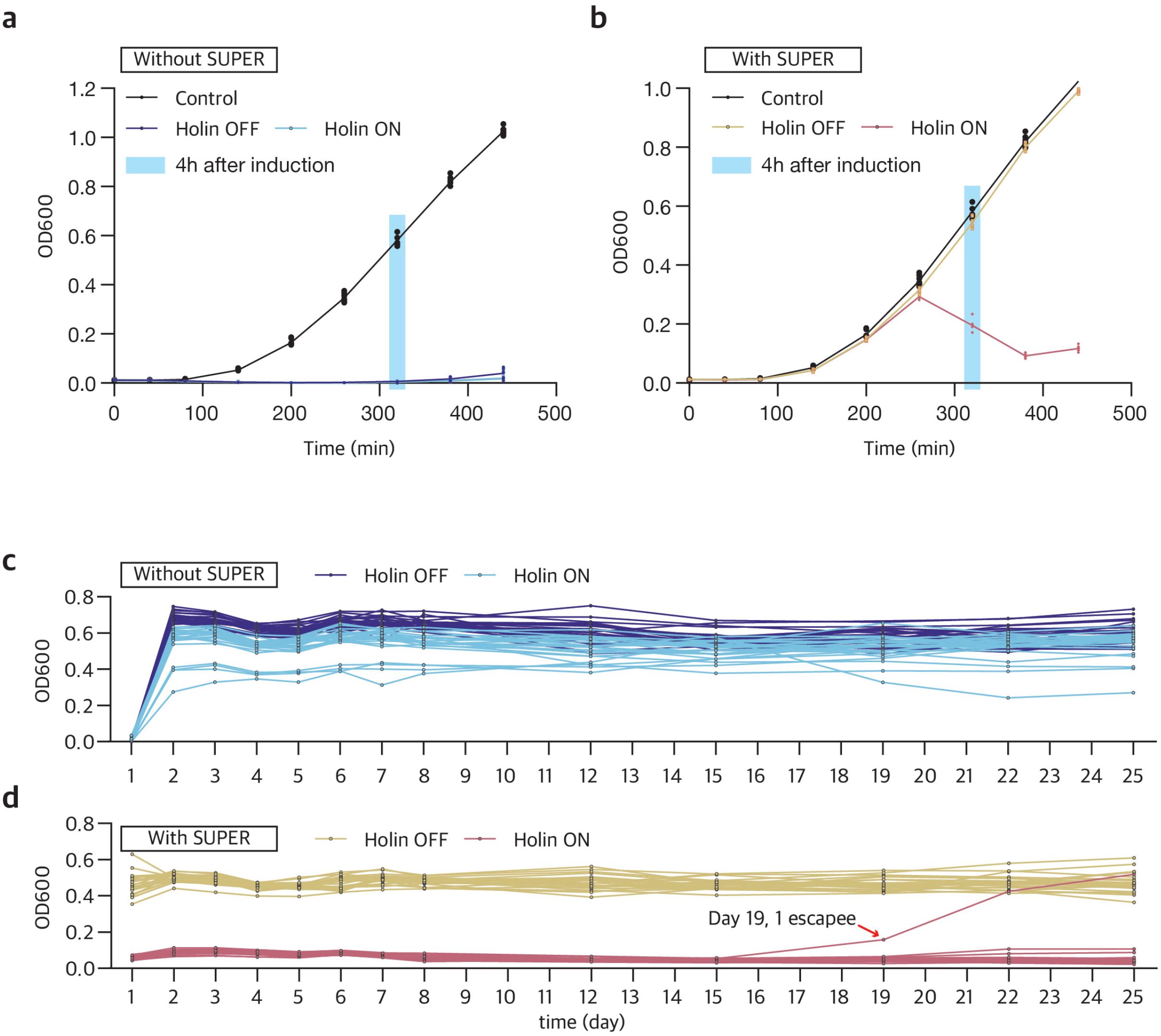


**Supplementary Figure S15.** Characteristics of the IPTG-inducible kill switch with or without SUPER.

(**a** and **b**) Cell growth of bacteria harboring an IPTG-inducible kill switch. The kill switch is constructed as described in **Figure 7**, either (**a**) without SUPER or (**b**) with SUPER. IPTG was added at 80 min, at concentrations of either 0 mM (Holin OFF) or 1 mM (Holin ON). As a control, bacteria lacking the kill switch were used. Eight biological replicates were performed. The dashed line represents the mean value across replicates. (**c**, **d**) OD600 analysis after 4 h induction for long-term genetic stability of the IPTG-inducible kill switch system (**c**) without SUPER and (**d**) with SUPER, measured over 25 days. A total of 24 biological replicates were conducted.

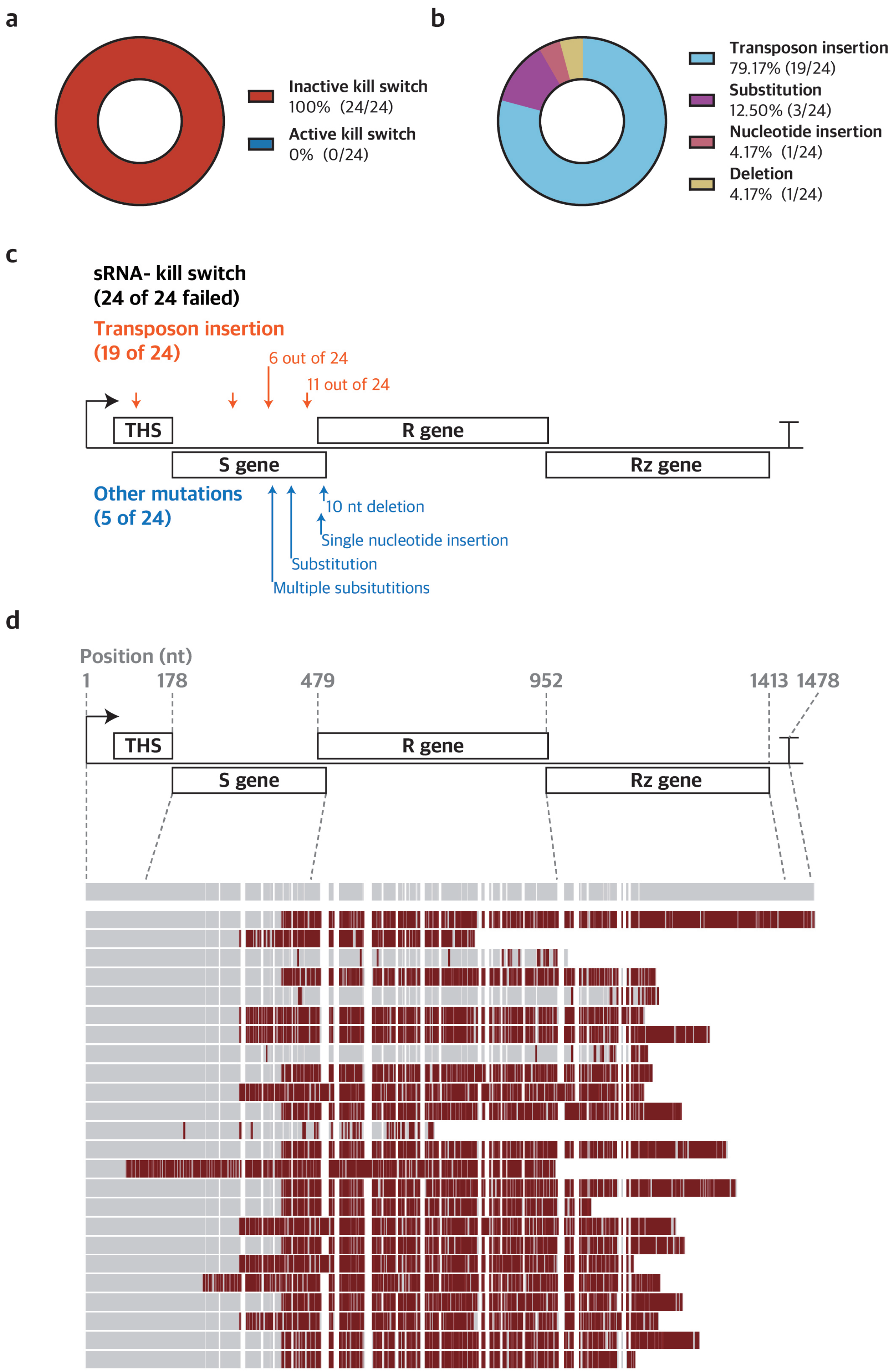
a
Inactive kill switch
100% (24/24)
Active kill switch
0% (0/24)
b
Transposon insertion
79.17% (19/24)
Substitution
12.50% (3/24)
Nucleotide insertion
4.17% (1/24)
Deletion
4.17% (1/24)
c
sRNA- kill switch
(24 of 24 failed)
Transposon insertion
(19 of 24)
6 out of 24
11 out of 24
THS
R gene
S gene
Rz gene
Other mutations
(5 of 24)
10 nt deletion
Single nucleotide insertion
Substitution
Multiple subsitutitions
d
Position (nt)
1
178
479
952
1413
1478
THS
R gene
S gene
Rz gene

**Supplementary Figure S16.** Summary of sanger sequencing results for the IPTG-inducible kill switch without SUPER after 25 days of passaging in **Supplementary Figure S15c**.

(**a**) The ratio of inactive to active kill switches on day 25. (**b**) Proportional distribution of mutation categories based on sequencing analysis of inactive kill switch samples. (**c**) Summary of Sanger sequencing results across all biological replicates. (**d**) Sanger sequencing results across all biological replicates. Red color indicates un-matched nucleotide. The number of biological replicates was twenty-four.

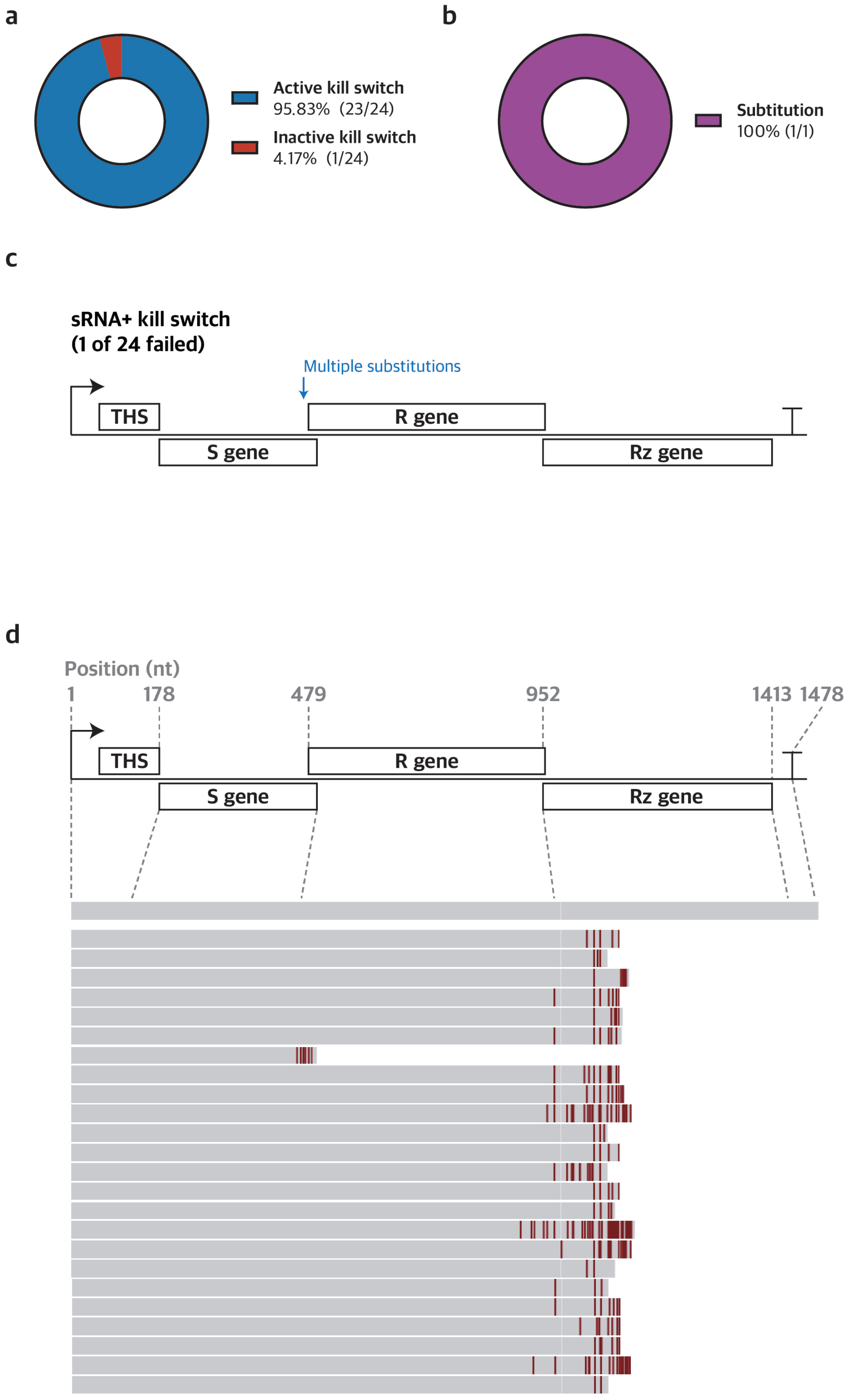
a
Active kill switch
95.83% (23/24)
Inactive kill switch
4.17% (1/24)
b
Subtitution
100% (1/1)
c
sRNA+ kill switch
(1 of 24 failed)
Multiple substitutions
THS
R gene
S gene
Rz gene
d
Position (nt)
1
178
479
952
1413
1478
THS
R gene
S gene
Rz gene

**Supplementary Figure S17.** Summary of sanger sequencing results for the IPTG-inducible kill switch with SUPER after 25 days of passaging in **Supplementary Figure S15d**.

(**a**) The ratio of inactive to active kill switches on day 25. (**b**) Proportional distribution of mutation categories based on sequencing analysis of inactive kill switch samples. (**c**) Summary of Sanger sequencing results across all biological replicates. (**d**) Sanger sequencing results across all biological replicates. Red color indicates un-matched nucleotide. The number of biological replicates was twenty-four.

a

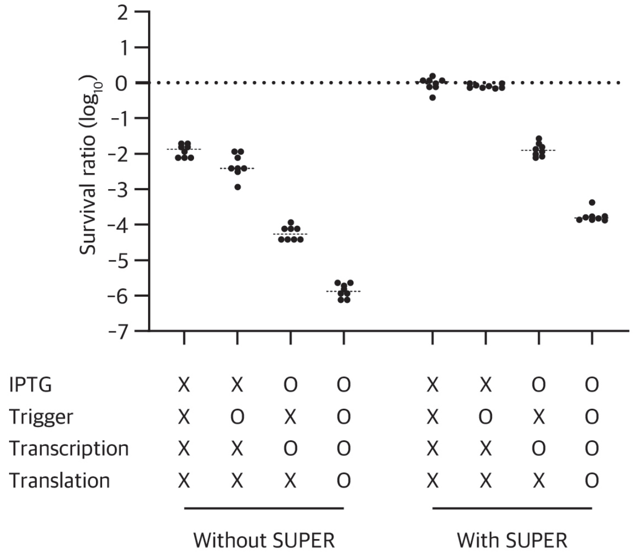


b

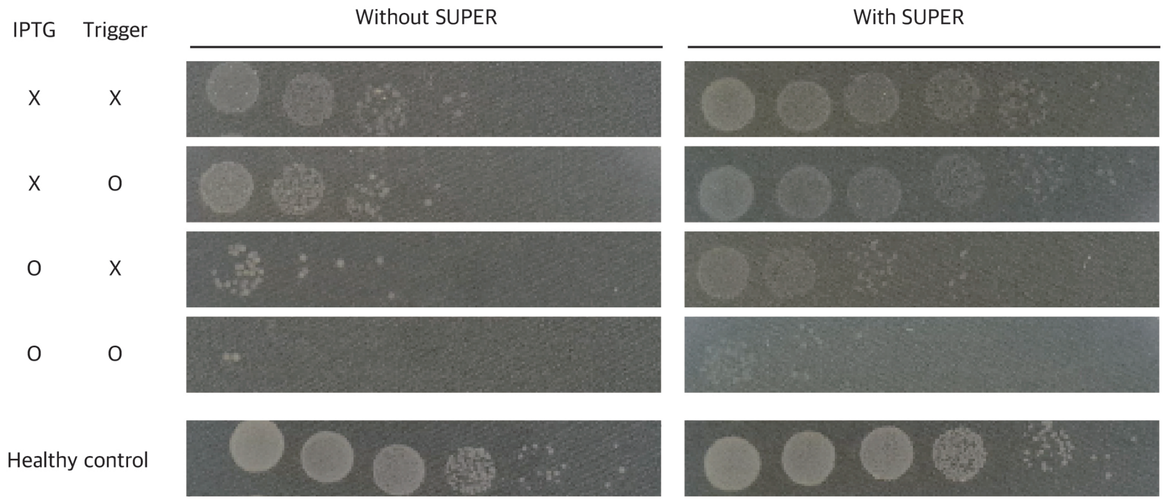


**Supplementary Figure S18.** Characterization of transcriptional and translational leakage in the kill switch. (**a**) Survival ratios of cells harboring the THS-based kill switch without or with SUPER under four regulatory input combinations: cognate trigger RNA or decoy RNA, each tested in the absence (0 mM) or presence (1 mM) of IPTG for 4 h. Error bars represent the standard deviation of eight biological replicates. (**b**) Representative CFU images from serial dilutions ($10^{-1}$ to $10^{-6}$, left to right) corresponding to the conditions shown in (**a**), illustrating the severe toxicity observed in the absence of SUPER.

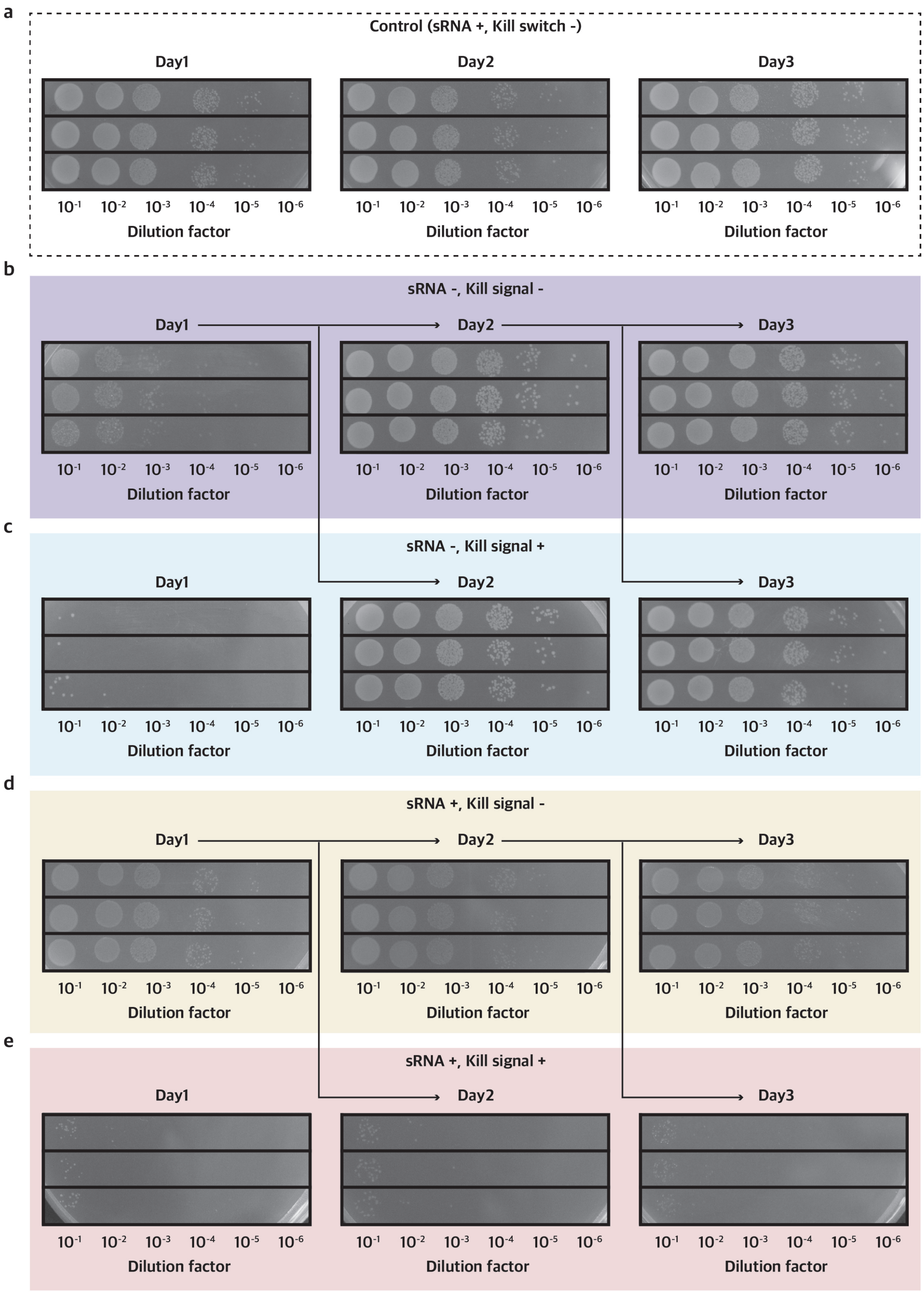
a
Control (sRNA +, Kill switch -)
Day1
Day2
Day3
10⁻¹ 10⁻² 10⁻³ 10⁻⁴ 10⁻⁵ 10⁻⁶
Dilution factor
b
sRNA -, Kill signal -
c
sRNA -, Kill signal +
d
sRNA +, Kill signal -
e
sRNA +, Kill signal +

**Supplementary Figure S19.** Representative image of cell viability assay through colony forming unit (CFU) measurement in Figure 7.

(**a**) CFU over 3 days in the kill switch non-harboring control *E. coli* DH5α. (**b-e**) CFU over 3 days in the kill switch harboring *E. coli* DH5α. Samples were under (**b**) sRNA- and kill signal- condition, (**c**) sRNA- and kill signal+ condition, (**d**) sRNA+ and kill signal- condition, or (**e**) sRNA+ and kill signal+ condition. Experiments and measurements were performed as described in Figure 7. This figure shows three representative replicates for each condition among the eight biological replicates. The CFU measurement images were not subjected to any post-processing, including adjustments to contrast or color.

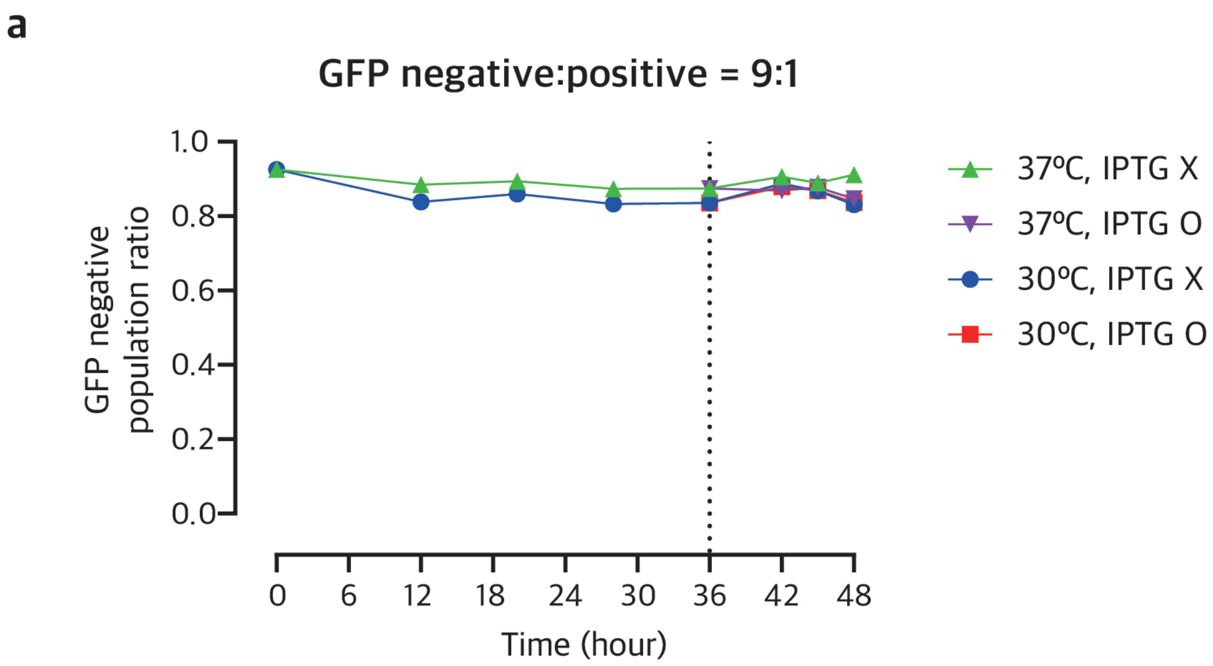


**Supplementary Figure S20.** Validation of population stability in microbial co-culture with GFP-labeled and unlabeled control strains. (**a**) *E. coli* control strains with (GFP+) or without (GFP-) constitutive GFP expression were mixed at an initial 9:1 ratio based on the GFP$^{-}$ population. The mixed cultures were incubated for 48 h at 30 °C and 37 °C. At 36 h, cultures were subdivided into parallel groups containing 0 mM or 1 mM IPTG. The kill switch-harboring strain (kill switch +) and a healthy control without a kill switch circuit (kill switch -) were mixed at a 9:1 ratio initially. Sampling was conducted every 6 h up to 48 h. The population ratio was measured via flow cytometry.

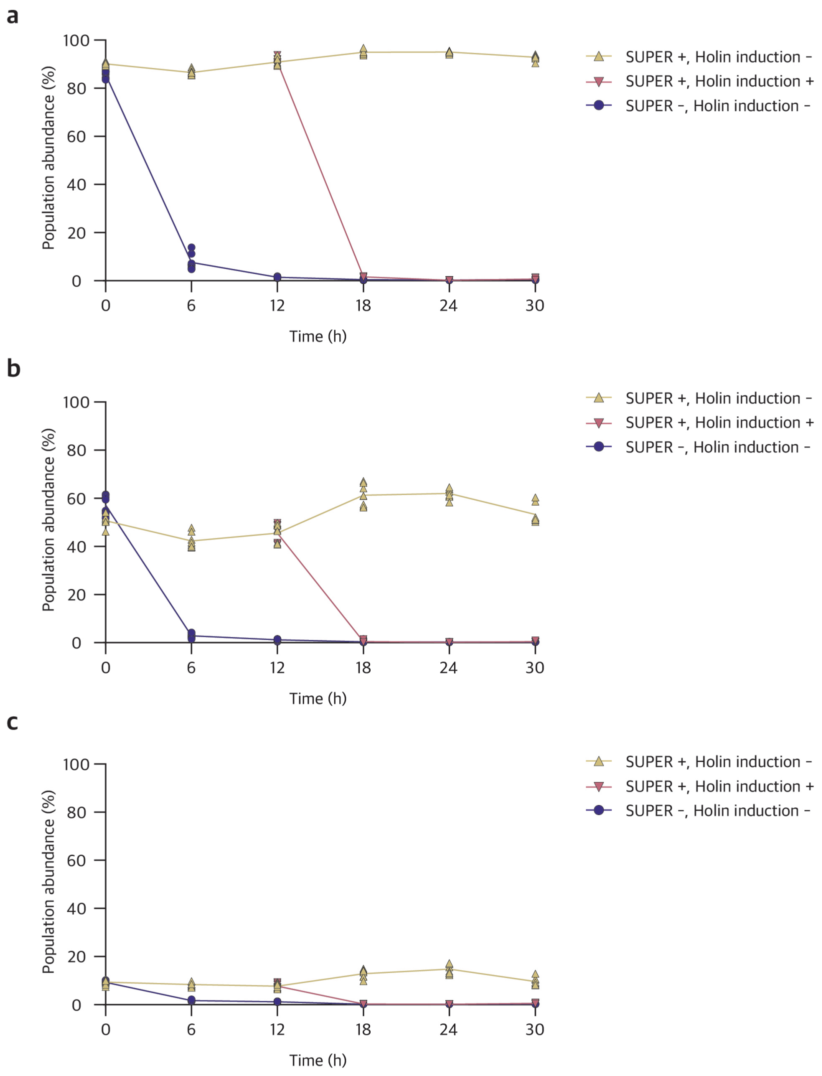


**Supplementary Figure S21.** Effect of initial population ratio on the stability of single-input kill switch in microbial co-culture. (**a-c**) Microbial co-culture experiments were conducted with varying initial population ratios of kill switch–harboring cells (GFP$^-$) to control cells (GFP$^+$): (a) 9:1, (b) 5:5, and (c) 1:9. Population dynamics were monitored by flow cytometry under the same experimental conditions described in **Figure 7j**.

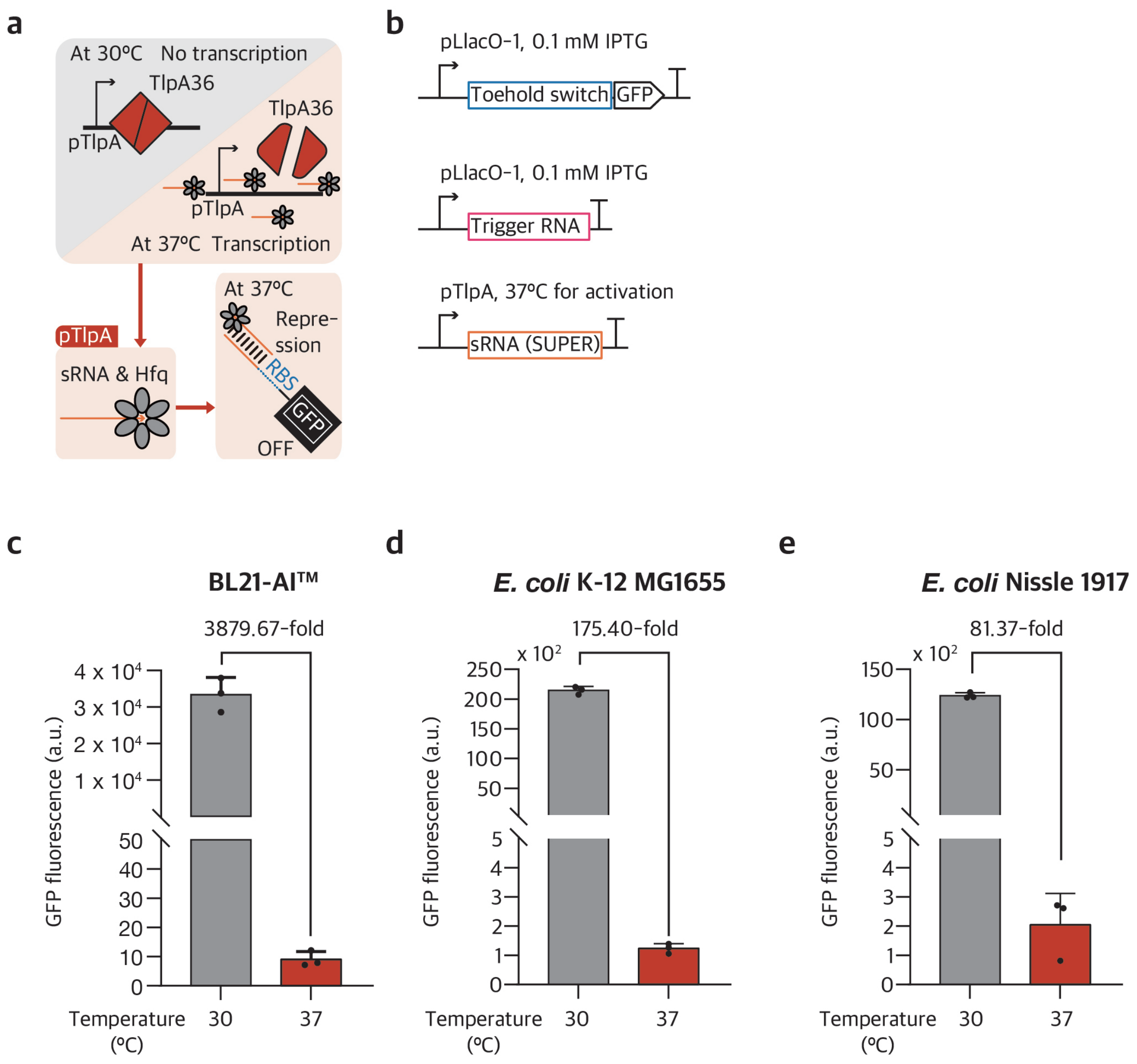


**Supplementary Figure S22.** Temperature-responsive sRNA expression system. (**a**) Schematics representation of temperature-responsive sRNA expression system. At 30°C, a TlpA36 dimer inhibits the transcription of sRNA form the pTlpA promoter. On the other hand, at 37°C, TlpA36 undergoes a structural change that prevents dimer formation, allowing active sRNA transcription. Since sRNA suppresses GFP expressions, high GFP expressions are expected only at 30°C. (**b**) Expression cassettes of temperature-responsive sRNA expression system. (**c-e**) *In vivo* characterization of the temperature-responsive sRNA expression system in *E. coli* BL21-AI$^{TM}$ (c), *E. coli* K-12 MG1655 (d), and *E. coli* Nissle 1917 (e). The number of biological replicates was three. Error bars indicate standard deviation.

**a**

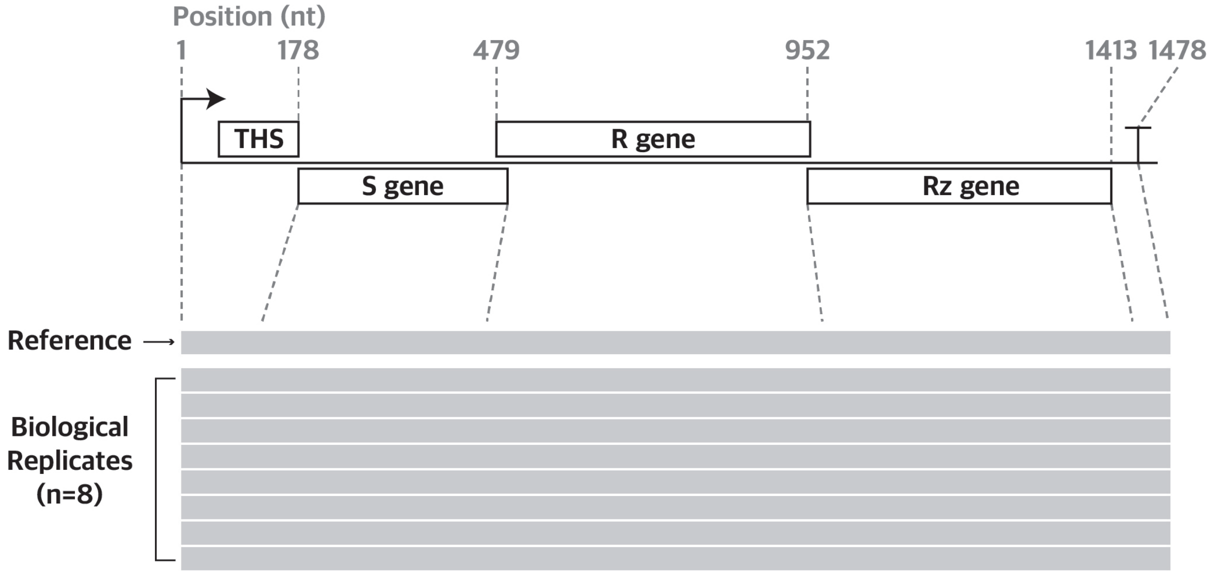


**Supplementary Figure S23.** Low-depth NGS analysis of the 2-input kill switch after prolonged cultivation. (**a**) Samples collected after 8 days of continuous culture under permissive conditions (37 °C, without IPTG) were subjected to low-depth next-generation sequencing (BITseq, Bionics, Seoul, Korea). No genetic mutations were detected within the promoter, UTR, or CDS regions of the kill switch circuit, confirming its evolutionary robustness during extended cultivation. The number of biological replicates is eight.

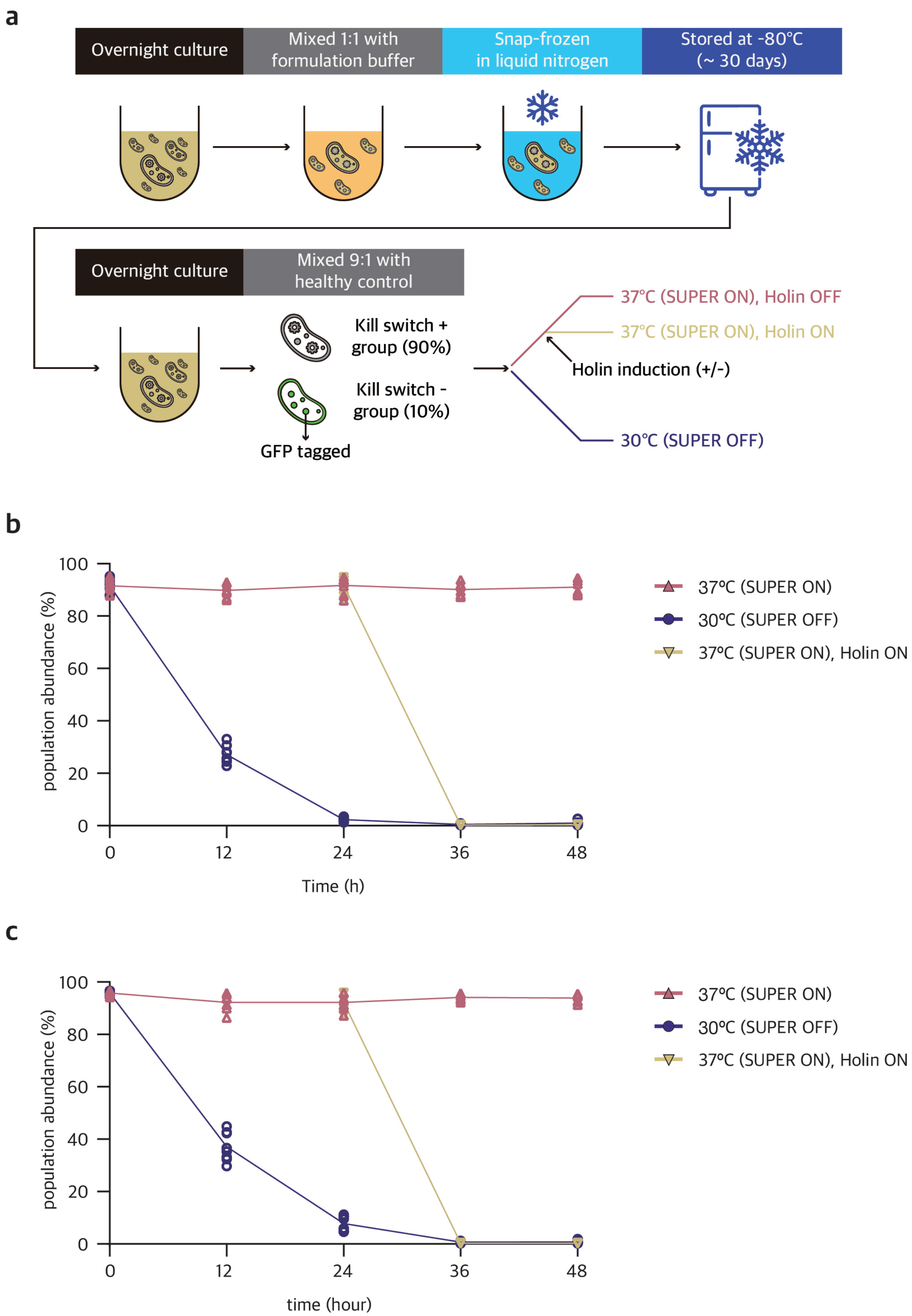
a
Overnight culture
Mixed 1:1 with formulation buffer
Snap-frozen in liquid nitrogen
Stored at -80°C (~ 30 days)
Overnight culture
Mixed 9:1 with healthy control
Kill switch + group (90%)
Kill switch - group (10%)
GFP tagged
37°C (SUPER ON), Holin OFF
37°C (SUPER ON), Holin ON
Holin induction (+/-)
30°C (SUPER OFF)
b
population abundance (%)
Time (h)
37°C (SUPER ON)
30°C (SUPER OFF)
37°C (SUPER ON), Holin ON
c
population abundance (%)
time (hour)
37°C (SUPER ON)
30°C (SUPER OFF)
37°C (SUPER ON), Holin ON

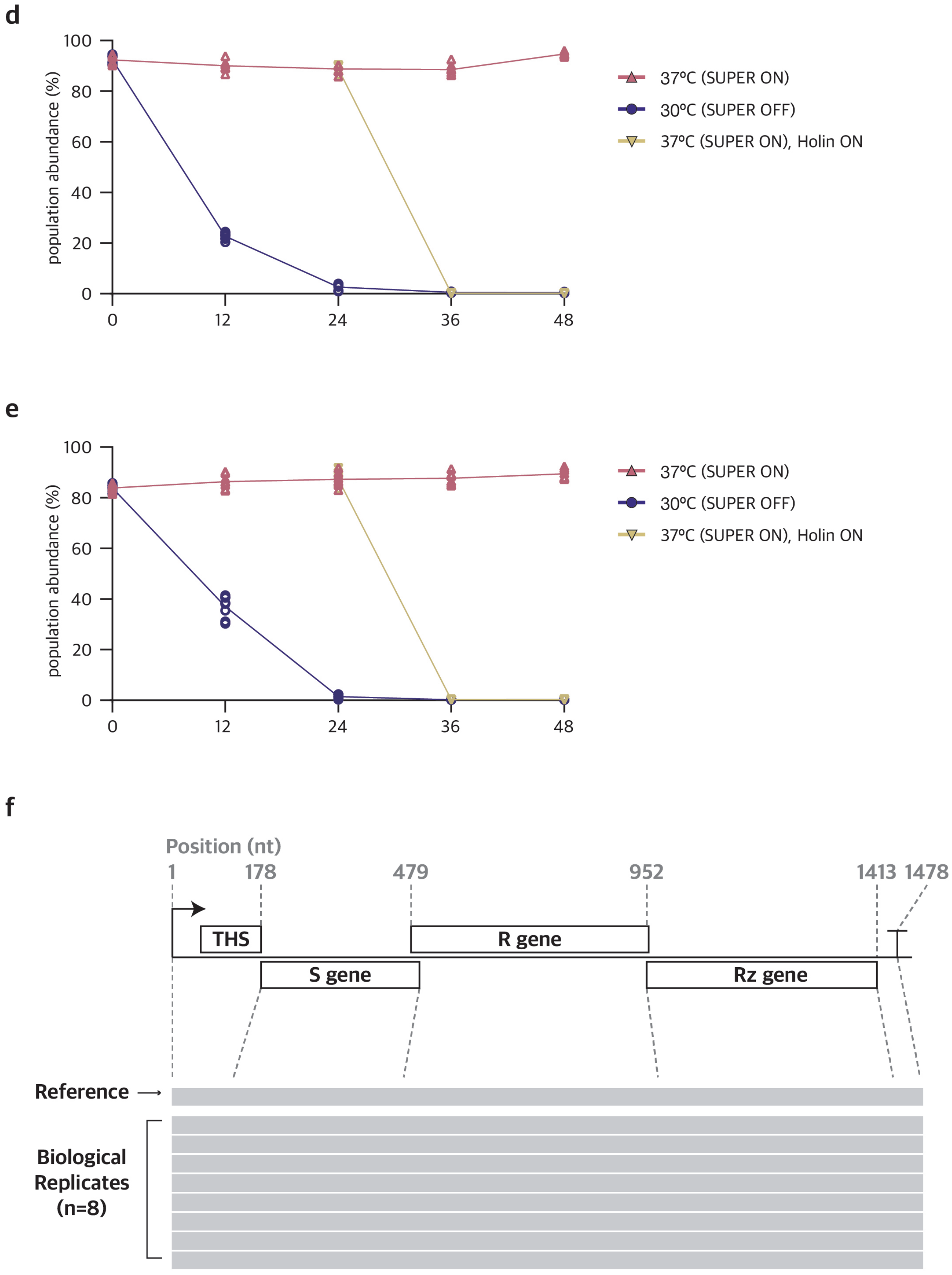


**Supplementary Figure S24.** Preservation and functional assessment of the 2-input kill switch under frozen storage conditions. (**a**) Schematic of the frozen stock preparation procedure, where overnight-

cultured cells harboring the 2-input kill switch were mixed 1:1 (v/v) with a formulation buffer ($KH_2PO_4$ 4.56 g/L, $K_2HPO_4$ 25 g/L, 30% glycerol, pH 7.5) and snap-frozen in liquid nitrogen before storage at –80 °C. (**b–e**) Functional evaluation of samples after 1 (b), 7 (c), 15 (d), and 30 days (E) of storage, respectively, under reconstituted microbial co-culture conditions to confirm retention of kill-switch functionality. (**f**) Low-depth next-generation sequencing (BITseq, Bionics, Seoul, Korea) of the 30-day samples revealed no detectable mutations in the promoter, UTR, or CDS regions of the kill switch circuit, demonstrating genetic stability after long-term frozen preservation. The number of biological replicates is eight.

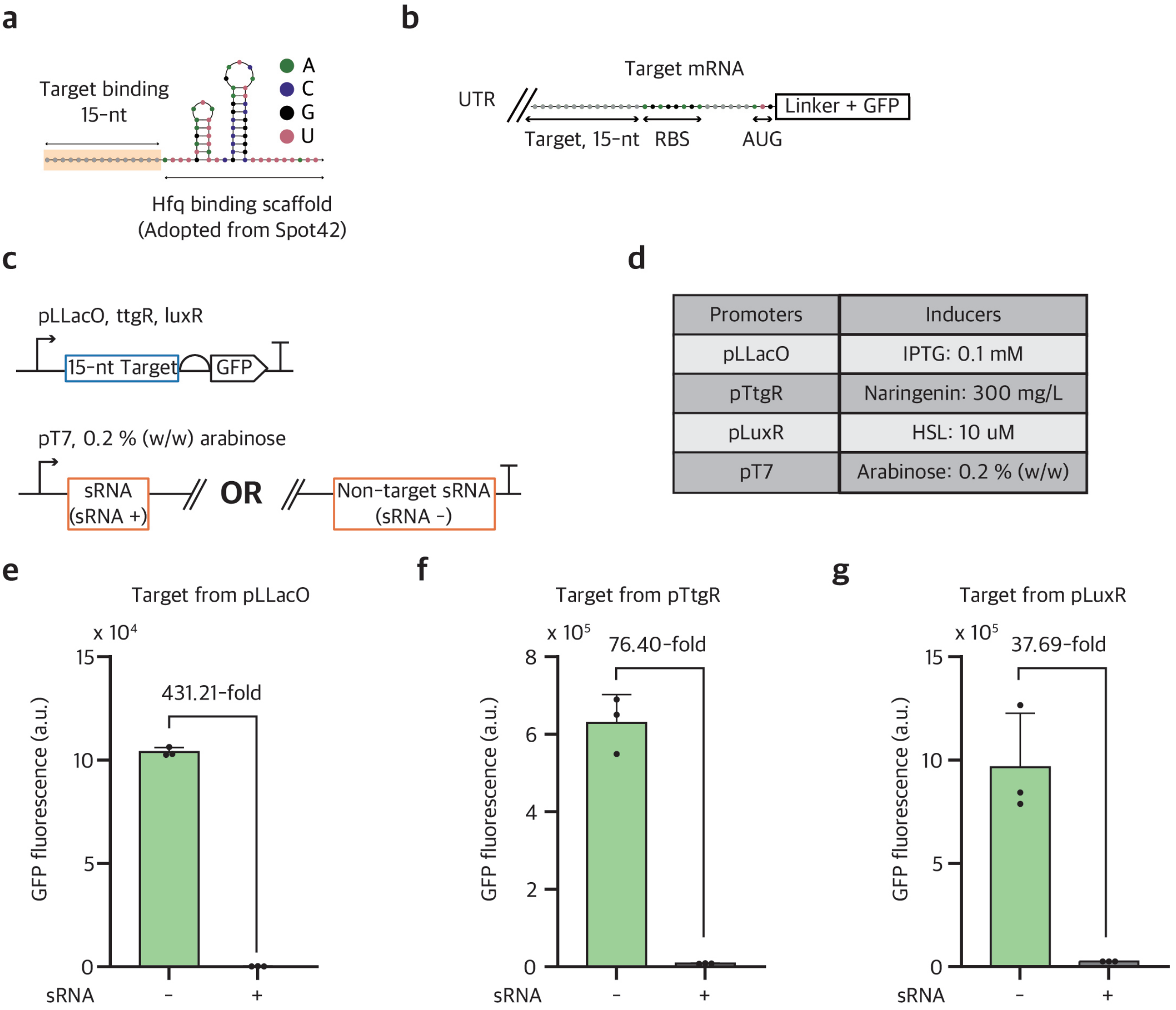


| Promoters | Inducers |
|---|---|
| pLLacO | IPTG: 0.1 mM |
| pTtgR | Naringenin: 300 mg/L |
| pLuxR | HSL: 10 uM |
| pT7 | Arabinose: 0.2 % (w/w) |

**Supplementary Figure S25.** Applicability of synthetic sRNA across different inducible promoter systems. (**a**) Secondary structure of the synthetic sRNA. (**b**) Secondary structure of its cognate target mRNA. (**c**) Expression cassettes showing the arrangement of the sRNA and target mRNA modules. (**d**) Induction conditions for each promoter (pLlacO, pTtgR, and pLuxR). (**e-g**) GFP fluorescence outputs in the presence or absence of the sRNA when the target mRNA was transcribed from pLlacO (**e**), pTtgR (**f**), and pLuxR (**g**). The same synthetic sRNA (variant 1) achieved robust repression across all promoter contexts without requiring sequence optimization or redesign. Error bars represent the standard deviation of three biological replicates.

**Reference**


1. Zadeh, J.N., Steenberg, C.D., Bois, J.S., Wolfe, B.R., Pierce, M.B., Khan, A.R., Dirks, R.M. and Pierce, N.A. (2011) NUPACK: Analysis and design of nucleic acid systems. *J Comput Chem*, **32**, 170-173.
2. Wolfe, B.R., Porubsky, N.J., Zadeh, J.N., Dirks, R.M. and Pierce, N.A. (2017) Constrained Multistate Sequence Design for Nucleic Acid Reaction Pathway Engineering. *J Am Chem Soc*, **139**, 3134-3144.
3. Fornace, M.E., Porubsky, N.J. and Pierce, N.A. (2020) A Unified Dynamic Programming Framework for the Analysis of Interacting Nucleic Acid Strands: Enhanced Models, Scalability, and Speed. *ACS Synth Biol*, **9**, 2665-2678.
4. Goh, H., Choi, S. and Kim, J. (2024) Synthetic translational coupling element for multiplexed signal processing and cellular control. *Nucleic Acids Res*, **52**, 13469-13483.
5. Green, A.A., Silver, P.A., Collins, J.J. and Yin, P. (2014) Toehold switches: de-novo-designed regulators of gene expression. *Cell*, **159**, 925-939.
6. Nakahira, Y., Ogawa, A., Asano, H., Oyama, T. and Tozawa, Y. (2013) Theophylline-dependent riboswitch as a novel genetic tool for strict regulation of protein expression in Cyanobacterium Synechococcus elongatus PCC 7942. *Plant Cell Physiol*, **54**, 1724-1735.
7. Rinnenthal, J., Klinkert, B., Narberhaus, F. and Schwalbe, H. (2011) Modulation of the stability of the Salmonella fourU-type RNA thermometer. *Nucleic Acids Res*, **39**, 8258-8270.
8. Kim, J., Seo, M., Lim, Y. and Kim, J. (2024) START: A Versatile Platform for Bacterial Ligand Sensing with Programmable Performances. *Adv Sci (Weinh)*, **11**, e2402029.
9. Piraner, D.I., Abedi, M.H., Moser, B.A., Lee-Gosselin, A. and Shapiro, M.G. (2017) Tunable thermal bioswitches for in vivo control of microbial therapeutics. *Nat Chem Biol*, **13**, 75-80.
10. Kim, J., Zhou, Y., Carlson, P.D., Teichmann, M., Chaudhary, S., Simmel, F.C., Silver, P.A., Collins, J.J., Lucks, J.B., Yin, P. *et al.* (2019) De novo-designed translation-repressing riboregulators for multi-input cellular logic. *Nat Chem Biol*, **15**, 1173-1182.
11. Chappell, J., Takahashi, M.K. and Lucks, J.B. (2015) Creating small transcription activating RNAs. *Nat Chem Biol*, **11**, 214-220.
12. Hong, S., Jeong, D., Ryan, J., Foo, M., Tang, X. and Kim, J. (2021) Design and Evaluation of Synthetic RNA-Based Incoherent Feed-Forward Loop Circuits. *Biomolecules*, **11**.
13. Ghodasara, A. and Voigt, C.A. (2017) Balancing gene expression without library construction via a reusable sRNA pool. *Nucleic Acids Res*, **45**, 8116-8127.
